# The growth diversity of preterm infants at 0–36 months corrected age in China: a real-world observational study

**DOI:** 10.3389/fped.2025.1506244

**Published:** 2025-01-31

**Authors:** Xia Wang, Shuwen Feng, Pu Yang, Yuxin Wang, Cong Wei, Junwen Zheng, Pin Liu, Lihong Liao, Xiao Yang, Peibin Xu, Junmei Bian, Xiaoping Luo, Yuanzhen Zhang, Dongchi Zhao

**Affiliations:** ^1^ Department of Pediatrics, Women and Children’s Hospital, Zhongnan Hospital, Wuhan University, Wuhan, China; ^2^ Children’s Digital Health and Data Center, Wuhan University, Wuhan, China; ^3^ Women and Children’s Hospital, Qingdao University, Wuhan, China; ^4^ Wuhan Tongren Hospital, Wuhan University, Wuhan, China; ^5^ Tongji Hospital, Huazhong University of Science and Technology, Wuhan, China; ^6^ Prenatal Diagnosis Center, Women and Children’s Hospital, Zhongnan Hospital, Wuhan University, Wuhan, China

**Keywords:** preterm infant, real-world, growth curve, body weight, length

## Abstract

**Background:**

Premature delivery interrupts the natural growth of the fetus. The postnatal healthy management of preterm infants still follows term standards after a postmenstrual age (PMA) of 40 weeks and there is a lack of research on the longitudinal dynamic postnatal growth tracks of preterm infants.

**Methods:**

Based on the database established by the Wuhan University Internet+ Early Childhood Development Alliance in China, information on preterm infants, including birth registration and health follow-ups from 2016 to 2022, was incorporated into the health management system. Standardized anthropometric measurements of preterm infants were recorded from birth to a corrected age (CA) of 36 months. A generalized additive model based on location, scale, and shape was used to establish the percentile values and growth curves.

**Results:**

In total, 79,514 preterm infants were included in this study, and the birth weights at each gestational age (GA) were similar to Chinese standards. When evaluated by term birth weight, we found that the proportions of extrauterine growth retardation at a PMA of 40 weeks were all above 10% in the GA ≤34-week groups and reached between 17.19% and 55.56% in very preterm infants (VPIs). There was a high incidence of preterm infants with a weight below the third percentile in VPIs when referring to term standards at CAs of 0, 6, 12, 24, and 36 months (*p* < 0.001). We established natural growth curves of the preterm population with different GAs between CAs of 0 and 36 months, which indicated that the weight/length of late preterm infants was close to term standards while the growth trajectory of VPIs consistently lagged behind (*p* < 0.001).

**Conclusion:**

Our study revealed the different growth trajectories of preterm infants with different GAs. A set of growth curves and percentile values for preterm infants of different GAs between CAs of 0 and 36 months were established, offering an optional method for growth assessment of this special population.

## Background

The postnatal growth assessment of preterm infants has been of great concern. Currently, the Fenton and INTERGROWTH-21st (Inter-21) growth curves are widely used in preterm infants to evaluate early postnatal growth from birth to 50 weeks old or older. After a postmenstrual age (PMA) of 40 weeks (term equivalent age of 0 months), normal-term infants’ physical growth standards are considered as a reference to define the physiological range of preterm infant growth by corrected age (CA) and growth failure (1–3). Reference ranges for childhood growth, released by the World Health Organization (WHO) and China in 2006 and 2022, respectively, are also currently extensively accepted assessment tools for measuring the physical growth of preterm infants in China (4, 5), and provide a basis for judging the early development status and identifying growth retardation.

Premature delivery interrupts the natural growth of the fetus. Currently, the postnatal healthy management of preterm infants still follows the fetal growth charts by ultrasound before a PMA of 40 weeks and the standards of normal-term infants after a PMA of 40 weeks (6, 7). However, preterm birth is an abnormal phenomenon, and it is widely accepted that the intrauterine and extrauterine growth rates are discordant (8). In particular, parenteral nutrition and high-intensity invasive treatments should be conducted in extremely and very preterm infants [VPI, gestational age (GA) of 28–31^+6^ weeks] for a relatively long period after birth and their growth is less likely to reach the ideal pattern of a healthy fetus or term infants in the early postnatal stage (9). A considerable proportion of preterm infants need to undergo a period of catch-up growth to reach the level of term infants at the same corrected age. It is still debated whether this pattern is optimal for all preterm infants and what their optimal growth should be (10–12).

Up to now, research on the physical growth of preterm infants has been mostly based on cross-sectional data, as there is a lack of research on the longitudinal dynamic postnatal growth tracks of preterm infants of different GAs (13). Extremely preterm infants (EPI, GA < 28 weeks) have a high proportion of extrauterine growth retardation (EUGR) when evaluated using the postnatal growth standards of term infants. However, this may represent a natural growth trajectory when compared to their own baseline development (14). Natural growth curves based on the actual postnatal growth of preterm infants could be more objective and appropriate for their growth evaluation, especially for those considered likely to deviate from normal trajectory (15–17).

Supported by the Wuhan University’s Children's Digital Health and Data Center (CDHDC), our study collected standardized anthropometric measurements of preterm infants from the 194 medical institutions of the Internet+ Early Childhood Development Alliance (I-ECDA) in China and focused on the characteristics of early physical growth in preterm infants during the period of 0 to 36 months corrected age, and established a set of growth curves of preterm infants at different GAs from real-world observations.

## Methods

### Study design and population

In this study, we used routine clinical data from the database of the Wuhan University’s I-ECDA. The alliance was under the guidance of a number of neonatologists, with the participation of 194 medical institutions from 11 provinces in China. This database holds a set of real-world data concerning the anthropometric measurements and general information of the registered children. Data were collected and recorded on specially designed forms in the children's health management system (0–6 Child Care Solutions Co. Ltd., Beijing, China) that were applied in all the institutions. Methods of anthropometric measurements were performed following the World Health Organization's protocols (18, 19). The Ethics Committee of Zhongnan Hospital of Wuhan University (Kelun 2022042K) approved the study.

The data of infants registered in the database between 1 January 2016 and 31 Dec 2022 from birth to a CA of 36 months at follow-up was used in our study. Examination data of preterm infants, singletons or multiple births, born between 25^+0^ and 36^+6^ weeks of gestation and survived to PMA 40 weeks without congenital malformations and severe postnatal morbidity (surgical necrotizing enterocolitis, severe hypoxic-ischemic encephalopathy, grade 3 or 4 periventricular hemorrhage, or hydrocephalus) were included. A PMA of 40 weeks was considered the original point, percentile values of growth index between CAs of 0 and 36 months were calculated, and growth curves of different sexes and GAs were fitted.

A total of 10,000 boys and 10,000 girls (unique IDs) who were healthy term infants born at 40^+0^ weeks of gestation during the same period were randomly selected as the control group. Data from their follow-ups at CAs of 0 to 36 months were exported from the database. Thus, data from follow-up visits of 27,393 male and 27,452 female infants were obtained. Their anthropometric measurement data were collected to establish the physical growth percentile curves of term infants and served as the control for this study. We also selected Inter-21 and Chinese newborns standard (CNS, 2020) as controls for preterm infants at birth (20, 21), and the child growth references released by the WHO and National Health Commission of China (NHCC, 2022) were used as controls for preterm infants between the CAs of 0 and 36 months (4, 5).

### Growth curve fitting

Generalized additive models for location, scale, and shape (GAMLSS) were applied to construct the percentile values and growth curves of length, weight, head circumference (HC), and body mass index (BMI) stratified by sex and GAs of preterm infants between CA 0–36 months (22–24). Based on the global deviation, Akaike information criterion (AIC), Bayesian information criterion (BIC), and Q-Q plot, models were comprehensively evaluated for fitting quality to choose the optimal models. After repeated comparison, Box–Cox T (BCT) distribution transformation was selected as the final choice.

### Statistical analysis

We extracted the data of preterm infants from 06 Growth Collection software for data organization and analysis. To exclude the influence of abnormal distortion and filling errors, data on length, weight, HC, and BMI exceeding the mean ± 5 SD of the same sex and age were removed (21, 25). SPSS 25.0 was used for the normal distribution test and basic statistical analysis (Mann–Whitney *U*-test). Values were represented by median and interquartile range (IQR). Percentile values and fitting curves of the length, weight, HC, and BMI of preterm infants at different GAs were obtained using the GAMLSS5.4–12 package on R4.2.3. Scatter plots, growth curves, or combination plots were created via Originpro 2023 with the obtained percentile values and original data.

### Ethics considerations

This study was approved by the Ethics Committee of Zhongnan Hospital of Wuhan University (Kelun 2022042K) and exempted from the requirement of signing the informed consent forms.

### Role of the funding source

The funders of the study had no role in study design, data collection, data analysis, data interpretation, or writing of the report. All the authors confirmed that they accepted the responsibility to submit for publication.

## Results

### General information

During the study period, a total of 1,034,217 newborns were registered in the database, of which preterm infants comprised 8.3% (85,945). Of the latter, 6,431 cases were excluded due to missing data, deviation over mean ± 5 SD, congenital malformation, and severe postnatal morbidity. Thus, 79,514 individuals were eligible for our study, with male and female preterm infants accounting for 56.7% (45,115) and 43.3% (34,399), respectively. Between the CAs of 0 and 36 months, there were 195,462 follow-up visits among the preterm infants. After excluding 15,096 due to unqualified data, 180,366 effective follow-up visits were obtained. Due to the small number of infants born before 28 weeks of gestation, they were merged into the EPI group for statistical purposes. The distribution of birth and follow-up visits for preterm infants at different GAs is shown in Table 1.

**Table 1:** Characteristics of the preterm infants born at a gestational age of 25–36 weeks, *n* = 79,512.

Gestational age (weeks)	Boys (*N* of preterm boys = 45,114)							Girls (*N* of preterm girls = 34,398)						
	*N* at birth (%)	*N* of follow-up (average times)	Birth weight (kg)			IUGR% (birth)	EUGR% (PMA40W)	*N* at birth (%)	*N* of follow-up (average times)	Birth weight (kg)			IUGR% (birth)	EUGR% (PMA40W)
			This study (IQR)	CNS 2020 (median)	21-Inter (median)					This study (IQR)	CNS 2020 (median)	21-Inter (median)		
25	35 (0.09)	121 (3.46)	0.83 (0.75,0.89)	0.82	–	5.71%	50.00%	32 (0.08)	120 (3.75)	0.79 (0.74,0.85)	0.72	–	3.13%	55.56%
26	94 (0.19)	291 (3.10)	0.95 (0.86,1.00)	0.92	–	3.19%	42.86%	64 (0.21)	160 (2.50)	0.90 (0.86,0.98)	0.83	–	1.56%	37.50%
27	275 (0.51)	710 (3.58)	1.08 (0.95,1.20)	1.03	0.67	3.27%	42.42%	176 (0.61)	463 (2.63)	1.00 (0.90,1.10)	0.94	0.61	7.39%	35.29%
28	611 (1.15)	1,510 (2.47)	1.20 (1.10,1.35)	1.15	0.83	6.71%	30.43%	397 (1.35)	1,137 (2.86)	1.12 (1.01,1.24)	1.07	0.76	7.05%	20.83%
29	797 (1.76)	2,110 (2.65)	1.33 (1.20,1.49)	1.29	1.00	7.90%	20.22%	606 (1.77)	1,642 (2.71)	1.25 (1.10,1.40)	1.20	0.91	9.24%	23.61%
30	1,158 (2.42)	2,912 (2.52)	1.50 (1.31,1.65)	1.45	1.19	7.51%	25.74%	834 (2.57)	2,283 (2.74)	1.40 (1.25,1.55)	1.35	1.09	11.63%	19.13%
31	1,569 (3.34)	4,004 (2.55)	1.70 (1.50,1.86)	1.62	1.39	8.60%	18.31%	1,149 (3.48)	3,132 (2.73)	1.57 (1.40,1.75)	1.52	1.27	9.83%	17.19%
32	2,613 (5.24)	6,022 (2.31)	1.90 (1.67,1.90)	1.81	1.60	9.18%	15.91%	1,802 (5.79)	4,481 (2.49)	1.78 (1.57,1.98)	1.69	1.46	9.38%	10.78%
33	3,573 (7.62)	8,314 (2.33)	2.10 (1.88,2.10)	2.01	1.81	8.20%	13.89%	2,621 (7.92)	6,206 (2.37)	1.97 (1.75,2.19)	1.89	1.65	10.26%	13.35%
34	6,328 (13.59)	14,795 (2.34)	2.30 (2.08,2.50)	2.23	2.04	9.47%	10.46%	4,673 (14.03)	10,491 (2.25)	2.19 (1.95,2.40)	2.11	1.86	8.41%	10.87%
35	9,707 (21.70)	21,753 (2.24)	2.54 (2.30,2.80)	2.47	2.26	9.26%	9.28%	7,465 (21.52)	16,092 (2.16)	2.40 (2.20,2.65)	2.34	2.07	8.44%	10.01%
36	18,354 (42.38)	39,564 (2.16)	2.80 (2.50,3.05)	2.71	2.50	9.67%	7.18%	14,579 (40.68)	30,682 (2.10)	2.65 (2.40,2.91)	2.58	2.28	8.24%	8.42%

Inter-21, INTERGROWTH-21st standard.

The number and distribution of different parameters among the preterm infants and average follow-up times at corrected ages between 0 and 36 months are presented. The proportion of IUGR at birth and EUGR at a PMA of 40 weeks in preterm infants for each GA group is demonstrated by referring to the CNS (2020).

First, we compared the birth weight of preterm infants with the CNS and Inter-21 reference ranges. The results showed that the weight of both the male and female preterm infants born at different GAs was slightly higher than the CNS and far exceeded the reference range of Inter-21 (*P* < 0.001). This was especially so for VPIs, as their birth weight was 20%–60% higher than the Inter-21 reference range. Next, according to the CNS (2020), we calculated the proportion of intrauterine growth retardation (IUGR) at birth and EUGR at PMA 40 weeks in preterm infants for each GA group and found that the proportion of IUGR was under 10% in all GA groups, while the proportion of EUGR in the GA ≤34-week groups was above 10% and reached between 17.19% and 55.56% in VPIs. Thus, a significant proportion of VPIs did not actually achieve the ideal growth status of term infants at a PMA of 40 weeks. Given the differences in physical growth level of preterm infants at the same corrected age to term from that of term infants, it is necessary to describe the real trajectory of their early physical growth.

### Growth characteristics of preterm infants at CAs of 0–36 months

All the preterm infants of the same sex were included as a whole population, and percentile values and predicted growth curves (PGC) for length, weight, HC, and BMI in male and female infants at CAs of 0 to 36 months were achieved. We analyzed their growth trajectory and compared it to the PGC of the term controls.

**Figure 1:**
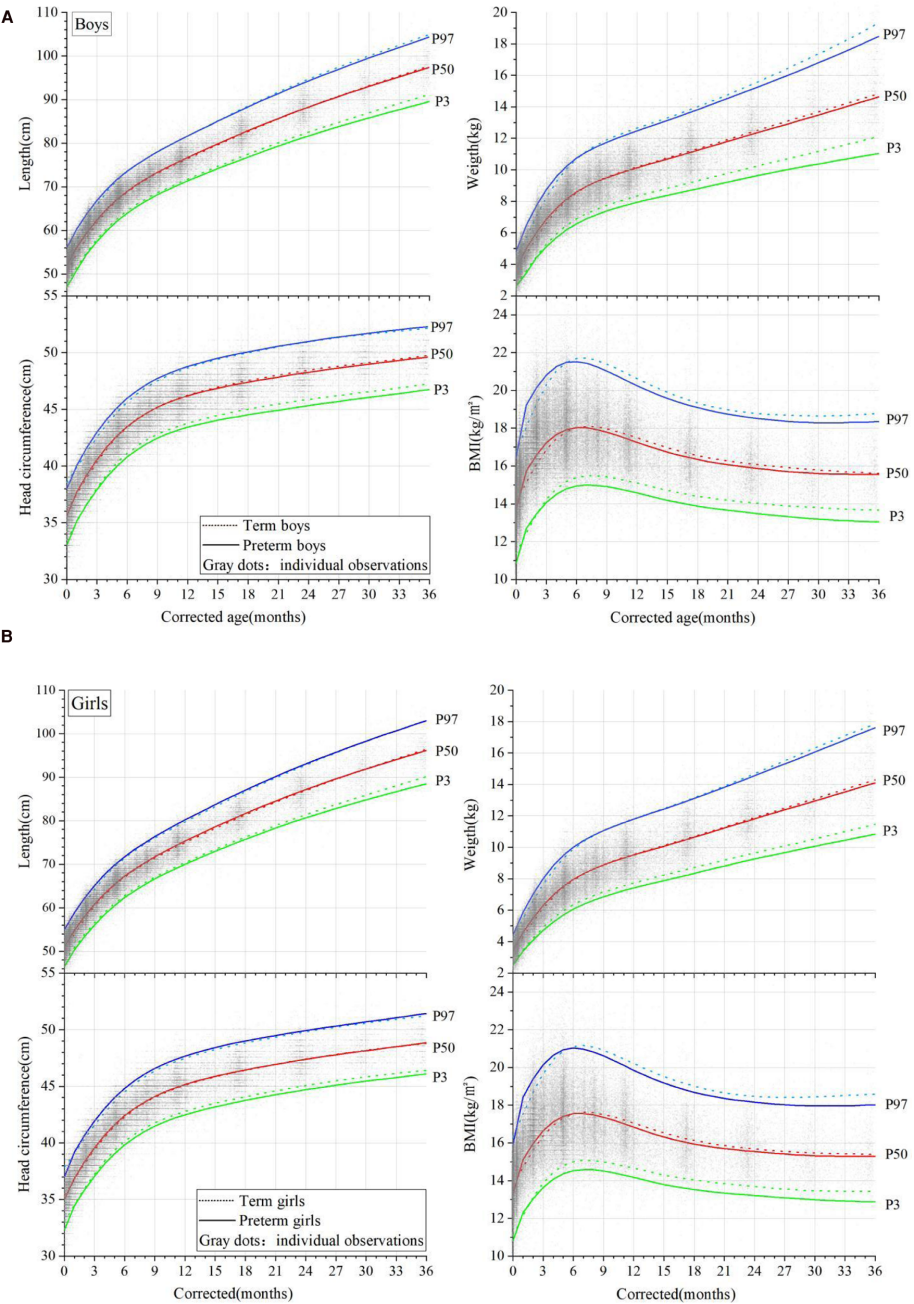
Comparison of the physical growth curves of preterm and term infants (corrected ages of between 0 and 36 months); **(A)** male infants and **(B)** female infants. Percentile curves (P3, P50, P97) of the length, weight, head circumference, and BMI are shown in the figures. The curves of the preterm infants are denoted by solid lines, and the term infants by dotted lines. Gray dots denote the individual observations.

At a CA of 0 months, the median length, weight, HC, and BMI of the male preterm infants were 52.0 cm, 3.7 kg, 35.6 cm, and 13.67 kg/m^2^, respectively (Supplementary Appendix Table 1). The length and weight at a CA of 0 months were both higher than those of term infants at the corresponding age (Supplementary Appendix Table 2) (*p* < 0.001) and exceeded the CNS and Inter-21 standards (*P* < 0.001). At CAs of between 1 and 6 months, the length increased by 1.86–4.18 cm per month and gradually slowed down with age. At a CA of 36 months, the length was slightly lower than that of the term infants (*p* = 0.478). The male preterm infants continued to have a higher weight than term infants at CAs of between 1 and 6 months, with a monthly increase of 850–1,300 g in the first 3 months. The weight gain slowed down from a CA of 7 months, with a monthly increase of 180–360 g, and was slightly lower than that of term infants at a CA of 36 months (*p* = 0.849). At a CA of 0 months, both the HC and BMI of the male preterm infants were smaller than those of term infants (*p* < 0.001). The HC exceeded that of term infants at CAs of between 1 and 8 months, with a monthly increase of 0.56–2.13 cm, and the rate of HC growth slowed down with age. The HC slightly lagged behind the term level at a CA of 36 months (*p* = 0.481). The BMI of the male preterm infants was almost the same as that of term infants at a CA of 0 months (*p* = 0.133) but was higher at CAs of between 1 and 6 months (*p* < 0.001). It gradually decreased after 7 months and became consistent with term infants at a CA of 36 months (Figure 1A). The growth trajectory of the female preterm infants was similar to that of male infants (Figure 1B), but their physical growth at all stages was lower than that of the male preterm infants (*p* < 0.001).

At CAs of between 1 and 5 months, the length and weight of the whole group of preterm infants reached or even exceeded that of term infants, and the rate of weight catch-up was faster than for length. At this stage, the BMI of both the male and female preterm infants was higher than that of the term infants, presenting with a relatively obese body shape. A comparison of the length/weight percentile distribution of the preterm infants showed that the median length and weight of the preterm infants at CAs of between 1 and 3 months were equivalent to P50-P75 in the term infant references.

### Assessment of preterm infant growth with term standards

We divided the preterm infants into four groups according to GA at birth, EPI (<28 weeks), VPI (28–31^+6^ weeks), moderate preterm infant (MPI, 32 to 33^+6^ weeks), and late preterm infant (LPI, 34 to 36^+6^ weeks). We then calculated the proportion of those with a weight and length that deviated from the 3% and 97% percentiles (P3, P97) at different CAs based on the PGC of the preterm infants, the PGC of the term infants, the WHO standards, and the NHCC standards.

**Figure 2:**
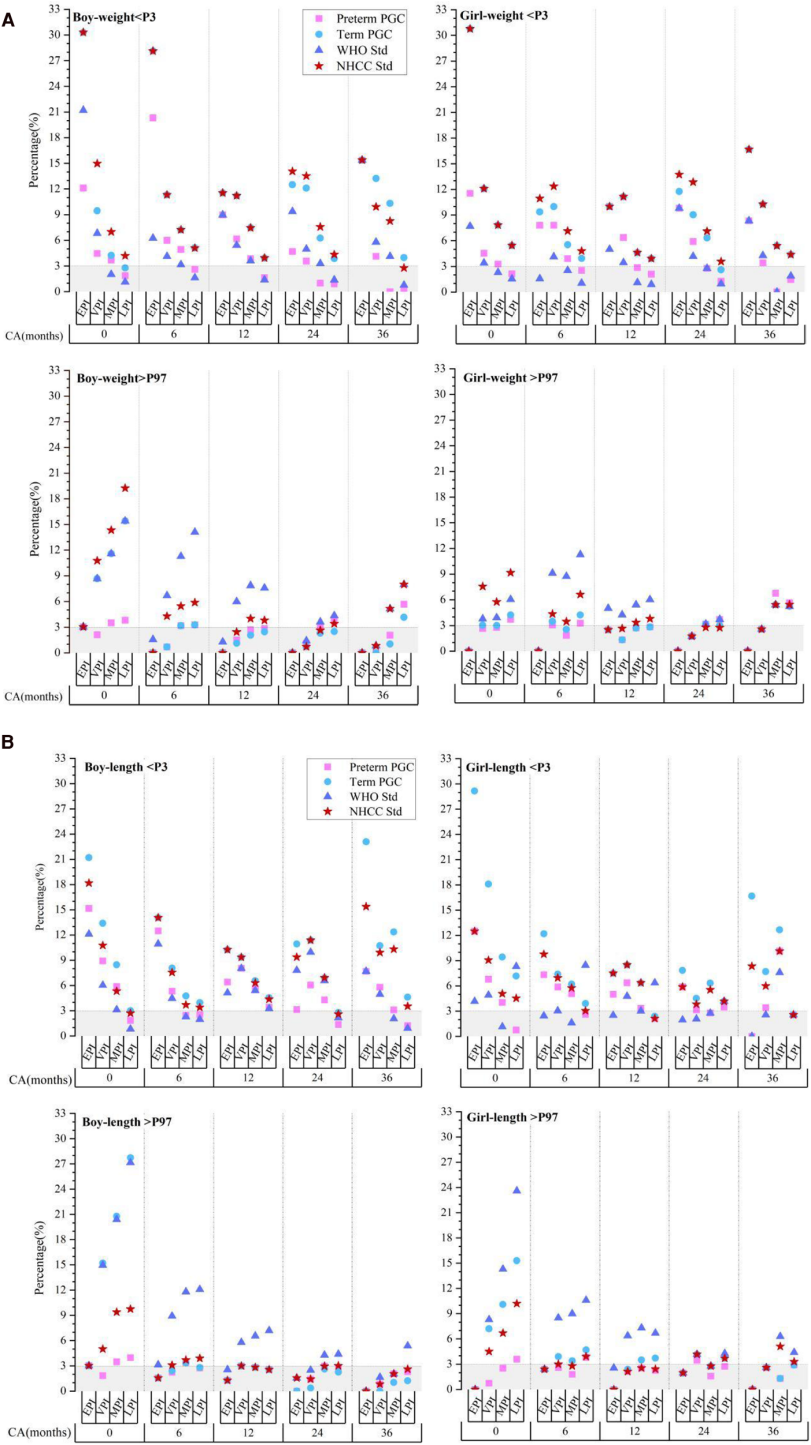
Evaluation of postnatal extreme growth deviation (below P3 or above P97) of weight **(A)** and length **(B)** in preterm infants evaluated using different standards. CA, corrected age; preterm PGC, predicted growth curve of preterm infants; term PGC, predicted growth curve of term infant; WHO Std., World Health Organization standards; NHCC Std., National Health Commission of China standards; EPI, extremely preterm infant; VPI, very preterm infant; MPI, moderate preterm infant; LPI, late preterm infant.

The results showed that although there were certain differences between each standard, the trend of growth deviation in preterm infants at CAs of 0–36 months was similar (Figures 2A,B). When assessed using the NHCC standard at CAs of 0, 6, 12, 24, and 36 months, the proportion of those with a weight less than P3 in male EPIs was 30.3%, 28.13%, 11.54%, 14.06%, and 15.38%, respectively. At CAs of 0 and 6 months, the incidence of growth retardation in EPIs was approximately 10 times that of the term infants and five times at a CA of 36 months. The proportion of VPIs in all stages was slightly lower than that of the EPIs but still well above 3%. That of LPIs was 4.19%, 5.1%, 3.93%, 4.34%, and 2.76% at CAs of 0, 6, 12, 24, and 36 months, respectively, and the overall trend was close to the term standards as age increased. At the same CA, the lower the GA at birth, the greater the proportion of those that had a weight less than the P3 (*p* < 0.001). For the proportion of those with a weight above the P97, it was next to nil in male EPIs at each CA stage, while that of LPIs was more than six times (19.24%) greater compared to term infants at a CA of 0 months. As age increased, this proportion gradually approached 3% at CAs of 6, 12, and 24 months (5.87%, 3.79%, and 3.4%), yet it slightly increased at a CA of 36 months (7.99%). At the same CA, the proportion of those with a weight above the P97 was positively associated with GA grade (*P* < 0.001). Similar trends of length deviation were observed.

The results of the female preterm infants were in accordance with those of the male preterm infants.

Compared to the term standards, the degree of growth deviation screened by the preterm PGC was slightly smaller, but the proportion of growth retardation in EPIs and VPIs was still much higher than 3% (*P* < 0.001). These results suggested that growth evaluation of preterm infants after a PMA of 40 weeks, either by term standards or preterm PGC, may exaggerate the incidence of growth retardation; however, the overestimation varied significantly between the different GA groups.

### Growth characteristics of preterm infants born at different GAs

**Figure 3:**
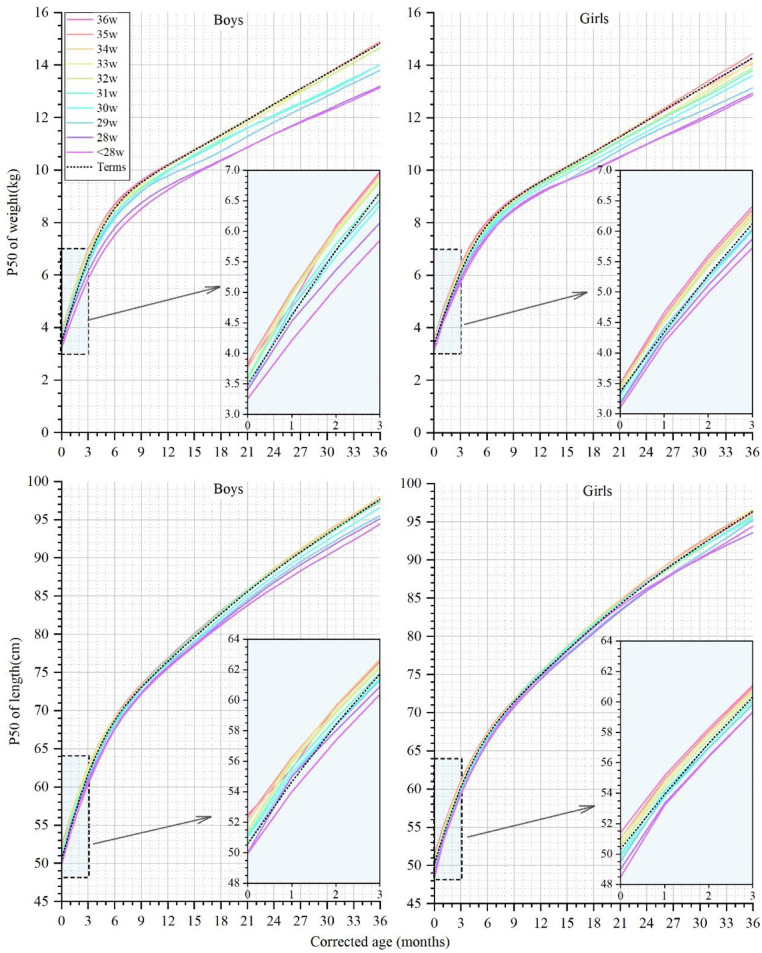
The 50th percentile curves for weight and length in male and female infants with different gestational ages at corrected ages between 0 and 36 months. The curves of preterm infants are denoted by colorful solid lines, and black dotted lines denote the term infant controls.

GA at birth is considered the most correlated factor with early growth. Meanwhile, significant differences in growth deviation degree by the same standard were found in our results above among different GA groups. Therefore, we observed the physical growth of preterm infants with different GAs, and the weight and length curves of the P50 at CAs between 0 and 36 months are shown in Figure 3.

At a CA of 0 months, the weight of the male preterm infants with a GA ≥30 weeks reached or exceeded the term infants, ranging from 3.50 to 3.84 kg (Supplementary Appendix Table 3). The weight of the male infants in the GA ≤33 weeks groups gradually fell behind term infants at CAs of 1 and 6 months and did not catch up to term infant level at a CA of 36 months. The weight of the female preterm infants with different GAs ranged from 3.10 to 3.50 kg at a CA of 0 months (Supplementary Appendix Table 4). The weight of the GA <32 weeks groups did not reach the level of newborns born at a CA of 0 months and consistently remained below the term infant weight up to a CA of 36 months. At a CA of 36 months, the median weight of the EPIs, male or female, lay in the P10-P25 of the term infants reference range.

At a CA of 0 months, the length of the male preterm infants ranged from 50.0 to 52.5 cm, and the length of all the GA groups reached or even surpassed the term infants (50.6 cm), except for the EPIs. At a CA of 36 months, the male preterm infants with a GA ≤31 weeks lagged behind, and the median length of the EPIs was equivalent to only the P10-P25 of the term infants. The length of the female preterm infants ranged from 48.5 to 51.4 cm at a CA of 0 months. The length of the female preterm infants with a GA ≥32 weeks exceeded that of the term infants at a CA of 0 months and gradually synchronized with the growth of the term infants. At a CA of 36 months, the median length of the infants with a GA <32 weeks did not reach the level of the term infants.

**Figure 4:**
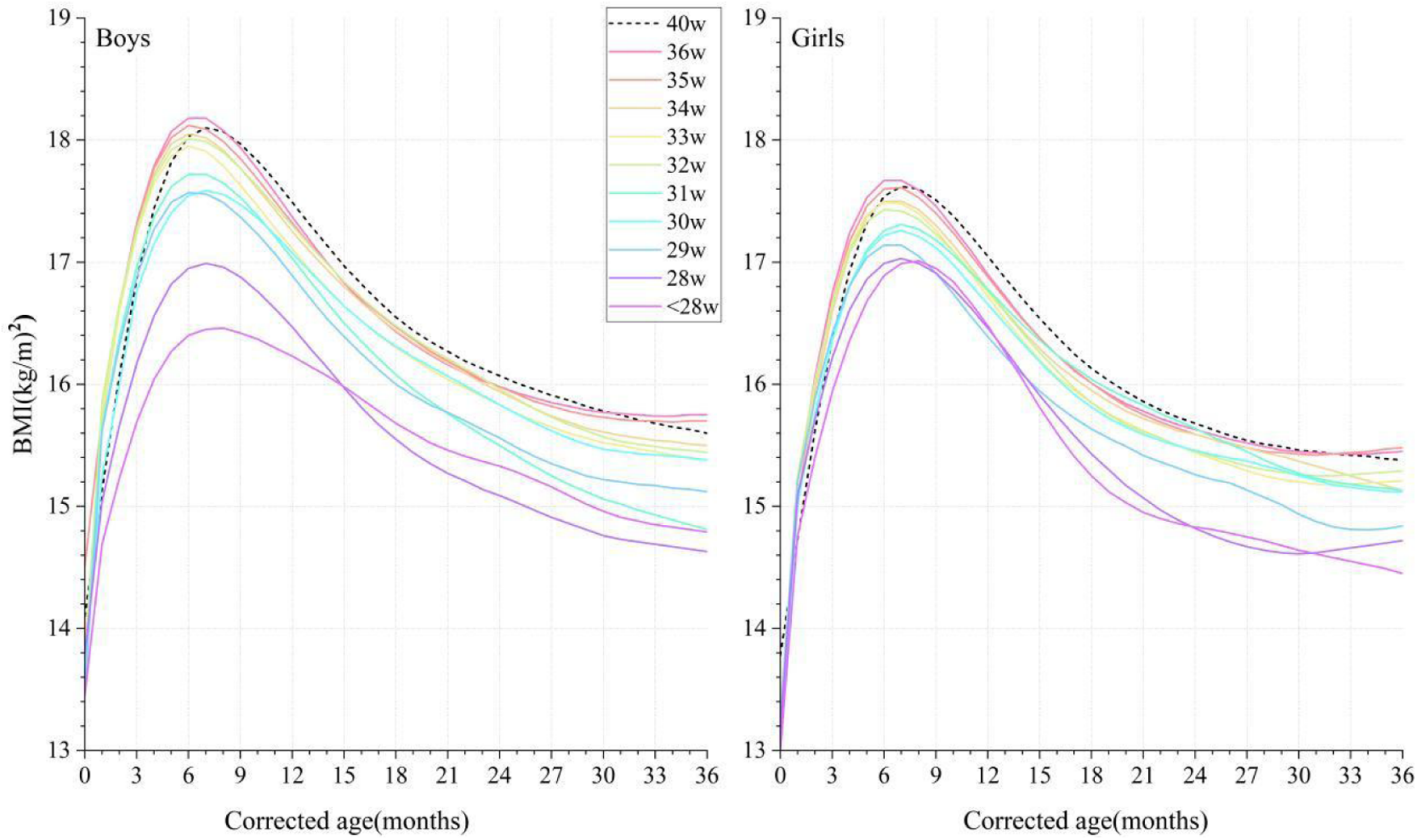
Comparison of the P50 curves for BMI in male and female infants with different gestational ages at corrected ages between 0 and 36 months. The BMI curves are denoted by colorful solid lines, and black dotted lines denote the term infant controls. BMI, body mass index.

At a CA of 0 months, the BMI of both the male and female preterm infants lagged behind the term infants but surpassed them between CAs of between 1 and 6 months in GA ≥32 weeks preterm groups. At CAs of 7–36 months, the BMI of the infants in the ≤34 weeks group was always smaller than that of the term infants, but the BMI of the infants in the GA ≥35 weeks group gradually exceeded that of term infants after a CA of 30 months (Figure 4).

## Discussion

In this observational study, we described the natural growth characteristics of preterm infants of different sexes and GAs in the real world between the CAs of 0 and 36 months. Different growth patterns were shown in preterm infants with different gestational ages and sexes, especially for VPIs. Considering these differences, we established a set of percentile curves for the physical growth of preterm infants and expect to offer a possible reference range conforming to their real physical postnatal growth patterns for this special group.

Despite this not being a rigorous longitudinal study, it systematically analyzed large-sample-size data obtained from at least one physical measurement at different time points in preterm infants at CAs between 0 and 36 months. GAMLSS, an emerging and widely applied method for constructing child growth reference curves, has the advantage that all data in the model can be utilized (22–24). Even in the GA<28 weeks group with a small sample size in our study, the accuracy and smoothness of the fitting growth curves by this method were guaranteed.

Currently, growth monitoring of infants is usually evaluated using anthropometric measurements such as weight, length, and HC. After analyzing large samples of preterm infants in recent years in China, we found that the weight of preterm infants born at different GAs was slightly higher than the CNS and far exceeded the reference range of Inter-21. This finding was also confirmed against the latest child growth standards in China, which may be related to multiple factors, such as improvement in socioeconomic levels, enhanced awareness of antenatal care among pregnant women, the increased level for preterm treatments, and the optimized nutrition for mothers and infants (5, 26). Growth retardation is defined as length divided by weight for age under the P3 or a *Z*-score less than 2 SD. For the healthy management of preterm infants, their ideal growth pattern should match that of a healthy fetus or term infant. However, this concept has not been substantiated by data and is seldom attained in practice (27–29). According to our statistics, the proportion of EUGR at a PMA of 40 weeks was above 10% in the GA ≤34-week groups when referenced to the CNS, and this proportion in VPIs reached 26.67%, significantly higher than the Fenton curves (21.37%) and Inter-21 curves (18.41%). Similar to our results, previous studies have shown that the prevalence of small for gestational age (SGA) or EUGR varies remarkably when assessed with different charts, and a high proportion of preterm infants could be defined as EUGR when referencing current standard curves (30–33). Kakatsaki et al. reported that 6.3% and 9.3% of 462 EPIs and VPIs were classified as SGA based on the Fenton/Inter-21 growth curves at birth, while 45.9% and 29.2% were classified as EUGR at discharge (31). Furthermore, when using term standards to evaluate the growth of preterm infants at CAs of between 0 and 36 months, the proportion of growth retardation in EPIs and VPIs was much higher than 3%. Thus, the growth trajectory of preterm infants did not exactly match the term infant curves or intrauterine growth standards. Monitoring the growth of preterm infants using term infant criteria according to CA may exaggerate the proportion of growth retardation (34). Previous methods of preterm growth assessment did not adequately take into account factors such as GA at birth, discordant growth rates between intrauterine and extrauterine development, and the inherent dynamic postnatal growth characteristics of preterm infants.

EPIs and VPIs account for only 10% of all preterm births, and they present with an obviously higher risk of growth deviation compared to MPIs and LPIs (35). They face greater survival challenges due to multiple factors such as neonatal care quality and invasive treatments, and nutritional support and neonatal complications could alter their growth trajectories. Considering all preterm infants as a whole to establish growth curves could not appropriately describe their natural growth. More individualized growth assessments of preterm infants with different GAs are needed.

Our comparison of the P50 growth curves between preterm infants and term control indicated that there were variances in growth trajectories of preterm infants with different GAs. The length and weight of LPIs surpassed that of term infants at CAs of between 0 and 6 months and then gradually joined the curves of the term infants. The trajectory of the MPI curves was basically in accordance with that of the term infants. The growth of the VPIs consistently lagged behind that of the term infants, and the weight of the EPIs was only equivalent to the 10% to 25% percentile range of the term infants at a CA of 36 months. During our observation period, GA was positively associated with weight and length, which was in line with findings from a Danish longitudinal study on 96,822 children (25).

Some studies have shown that the BMI of preterm infants is consistently lower than term infants before 7 years of age, and this gap decreased with age, which was not completely accordant with our findings (25, 36). Our result demonstrated a quicker increase and temporary precedence of BMI in preterm infants with a GA ≥32 weeks at CAs between 1 and 6 months compared to term infants. This specific phenomenon was consistent with the results of previous studies (24). It was reported that preterm infants had a higher fat mass and percent-fat at PMAs of 40–52 weeks than term infants (37–41). Hamatschek et al. supposed that rapid fat mass accrual in preterm infants may be an adaptive response to the *ex utero* environment (39). Therefore, it is reasonable to consider that this temporary difference in BMI may represent a natural growth trajectory when compared to their own baseline development. Nevertheless, some experts are concerned that excessive catch-up growth and overnutrition may increase their susceptibility to being overweight and long-term metabolic syndrome (42–47). O'Shea et al. observed that very high weight gain in the first 12–48 months after NICU discharge was associated with a higher risk of obesity at follow-up (48). Embleton et al. found that individuals born prematurely may have higher fat mass percentage, fat mass index, waist circumference, higher fasting insulin, blood pressure, and lower insulin sensitivity during adolescence (45). Even so, given the importance of brain growth in the neonatal period and infancy, catch-up growth should not be discouraged (48). Thus, for the healthy management of preterm infants during the early postnatal period, developing strategies to achieve reasonable catch-up growth patterns and reduce the risk of long-term metabolic syndrome should be further explored.

There were some limitations in our study. First, due to insufficient follow-up data for infants born with a GA <28 weeks, a subgroup analysis between EPIs could not be conducted. Second, multiple factors such as maternal health, mother's age, delivery mode, genetic factors, infant feeding, neonatal complications, medical intervention, special infant groups (twins/multiple births), geographical distribution, and socioeconomic factors were not explored in the present study. The article mainly focused on the growth characteristics of preterm infants with different gestational ages via a recent large sample in China. We will conduct continuous follow-ups of the preterm infants and expand the information on relevant factors in the database to conduct further studies.

In summary, our real-world observational study described the natural physical growth characteristics of preterm infants, explained their differences when compared with term infants, and revealed the different growth trajectories of preterm infants with different GAs. A set of growth curves and percentile values for preterm infants with different GAs at CAs of between 0 and 36 months were established, offering an optional method for growth assessment of this special population.

## Data Availability

The datasets presented in this study can be found in online repositories. The names of the repository/repositories and accession number(s) can be found in the article/Supplementary Material.
