# Supplementary material for: The growth diversity of preterm infants at 0–36 months corrected age in China: a real-world observational study
Source: Front Pediatr. 2025 Jan 31;13:1506244. doi: 10.3389/fped.2025.1506244 (PMC11825782; doi:10.3389/fped.2025.1506244)
Supplement: Supplementary file 1 [file Datasheet1.pdf]

The Postnatal Growth Reference for Preterm Infants

Length (boys)

Corrected age(months)	Centiles						
	3rd	10rd	25rd	50rd	75rd	90rd	97rd
0	47.00	49.00	50.50	52.00	53.50	54.70	56.00
1	51.00	53.12	54.63	56.18	57.68	59.04	60.44
2	54.59	56.31	57.86	59.46	60.99	62.39	63.83
3	57.45	59.20	60.78	62.40	63.97	65.39	66.86
4	59.96	61.73	63.32	64.96	66.54	67.98	69.48
5	62.12	63.89	65.49	67.14	68.74	70.19	71.70
6	63.96	65.73	67.34	69.00	70.60	72.06	73.58
7	65.54	67.32	68.93	70.60	72.21	73.68	75.21
8	66.92	68.71	70.34	72.01	73.64	75.12	76.66
9	68.16	69.97	71.61	73.30	74.94	76.44	77.99
10	69.29	71.13	72.79	74.50	76.16	77.68	79.25
11	70.36	72.22	73.90	75.64	77.32	78.86	80.46
12	71.36	73.25	74.97	76.73	78.45	80.01	81.64
13	72.33	74.25	76.00	77.80	79.55	81.14	82.79
14	73.26	75.23	77.01	78.84	80.62	82.25	83.93
15	74.18	76.18	78.00	79.87	81.68	83.34	85.06
16	75.08	77.12	78.97	80.88	82.73	84.42	86.17
17	75.96	78.04	79.93	81.88	83.76	85.48	87.26
18	76.82	78.95	80.87	82.85	84.76	86.51	88.33
19	77.67	79.83	81.78	83.80	85.75	87.53	89.37
20	78.50	80.69	82.68	84.73	86.71	88.52	90.39
21	79.31	81.54	83.56	85.64	87.66	89.49	91.39
22	80.11	82.38	84.43	86.54	88.58	90.44	92.37
23	80.89	83.19	85.27	87.41	89.49	91.38	93.33
24	81.65	83.98	86.09	88.27	90.37	92.29	94.27
25	82.38	84.75	86.89	89.10	91.23	93.18	95.19
26	83.10	85.50	87.67	89.91	92.07	94.04	96.09
27	83.79	86.23	88.44	90.70	92.90	94.90	96.97
28	84.47	86.95	89.18	91.48	93.71	95.73	97.83
29	85.14	87.65	89.92	92.25	94.50	96.55	98.68
30	85.79	88.34	90.64	93.00	95.29	97.37	99.52
31	86.43	89.01	91.34	93.74	96.06	98.16	100.35
32	87.06	89.68	92.04	94.47	96.82	98.95	101.16
33	87.69	90.34	92.73	95.19	97.57	99.73	101.97
34	88.31	91.00	93.42	95.91	98.32	100.51	102.78
35	88.93	91.65	94.11	96.63	99.07	101.29	103.58
36	89.55	92.31	94.80	97.35	99.82	102.06	104.38

By Wuhan University Children’s Digital Health and Data Center (CDHDC)

The Postnatal Growth Reference for Preterm Infants

WUHAN UNIVERSITY

Weight (boys)

Corrected age(months)	Centiles						
	3rd	10rd	25rd	50rd	75rd	90rd	97rd
0	2.60	2.99	3.30	3.70	4.10	4.45	4.80
1	3.44	4.09	4.52	5.00	5.48	5.95	6.46
2	4.43	4.96	5.46	6.01	6.57	7.11	7.71
3	5.13	5.71	6.27	6.88	7.50	8.10	8.75
4	5.71	6.33	6.93	7.58	8.25	8.88	9.58
5	6.19	6.84	7.47	8.14	8.84	9.51	10.23
6	6.57	7.25	7.89	8.59	9.31	9.99	10.74
7	6.89	7.58	8.24	8.95	9.68	10.38	11.14
8	7.16	7.86	8.52	9.25	9.99	10.70	11.47
9	7.39	8.09	8.77	9.50	10.25	10.97	11.75
10	7.59	8.30	8.99	9.73	10.49	11.21	12.01
11	7.77	8.50	9.19	9.94	10.71	11.44	12.24
12	7.93	8.67	9.37	10.13	10.91	11.65	12.47
13	8.09	8.83	9.55	10.32	11.11	11.86	12.69
14	8.23	8.99	9.72	10.50	11.30	12.07	12.91
15	8.37	9.15	9.88	10.68	11.50	12.28	13.13
16	8.52	9.30	10.05	10.87	11.70	12.49	13.36
17	8.66	9.46	10.22	11.05	11.90	12.70	13.59
18	8.80	9.62	10.39	11.24	12.10	12.92	13.82
19	8.94	9.77	10.56	11.42	12.30	13.14	14.05
20	9.08	9.93	10.74	11.61	12.50	13.36	14.29
21	9.22	10.09	10.91	11.80	12.71	13.58	14.53
22	9.36	10.24	11.08	11.99	12.91	13.80	14.77
23	9.50	10.40	11.25	12.18	13.12	14.03	15.02
24	9.64	10.55	11.42	12.37	13.33	14.25	15.26
25	9.77	10.70	11.59	12.55	13.54	14.48	15.51
26	9.90	10.85	11.75	12.74	13.74	14.71	15.76
27	10.02	10.99	11.92	12.92	13.95	14.94	16.01
28	10.14	11.13	12.08	13.11	14.16	15.17	16.27
29	10.26	11.27	12.24	13.30	14.37	15.40	16.53
30	10.37	11.41	12.41	13.48	14.59	15.64	16.80
31	10.49	11.55	12.57	13.67	14.80	15.88	17.07
32	10.60	11.69	12.73	13.86	15.02	16.13	17.34
33	10.71	11.83	12.90	14.05	15.24	16.38	17.62
34	10.82	11.97	13.06	14.25	15.46	16.63	17.90
35	10.93	12.10	13.22	14.44	15.68	16.88	18.19
36	11.04	12.24	13.39	14.63	15.91	17.14	18.48

By Wuhan University Children's Digital Health and Data Center (CDHDC)

The Postnatal Growth Reference for Preterm Infants

WUHAN UNIVERSITY

Head circumference(boys)

Corrected age(months)	Centiles						
	3rd	10rd	25rd	50rd	75rd	90rd	97rd
0	33.00	34.00	35.00	35.60	36.50	37.00	38.00
1	35.18	36.08	36.89	37.73	38.55	39.30	40.08
2	36.62	37.53	38.36	39.22	40.05	40.81	41.61
3	37.92	38.84	39.68	40.55	41.40	42.17	42.98
4	39.05	39.98	40.83	41.70	42.56	43.34	44.16
5	40.01	40.94	41.80	42.68	43.54	44.33	45.16
6	40.80	41.74	42.60	43.49	44.36	45.15	45.98
7	41.46	42.40	43.27	44.16	45.04	45.84	46.67
8	42.00	42.95	43.82	44.72	45.60	46.40	47.24
9	42.45	43.41	44.28	45.19	46.07	46.88	47.72
10	42.83	43.79	44.67	45.58	46.46	47.28	48.12
11	43.15	44.12	45.00	45.91	46.80	47.62	48.47
12	43.42	44.39	45.28	46.19	47.09	47.91	48.77
13	43.65	44.63	45.52	46.44	47.34	48.17	49.03
14	43.85	44.83	45.73	46.66	47.57	48.40	49.27
15	44.03	45.02	45.92	46.86	47.77	48.61	49.49
16	44.20	45.19	46.10	47.05	47.96	48.81	49.69
17	44.35	45.35	46.27	47.22	48.14	48.99	49.88
18	44.50	45.51	46.43	47.38	48.31	49.17	50.06
19	44.64	45.65	46.58	47.54	48.47	49.33	50.23
20	44.78	45.80	46.73	47.69	48.63	49.49	50.39
21	44.91	45.94	46.87	47.84	48.78	49.65	50.55
22	45.05	46.08	47.02	47.99	48.93	49.80	50.70
23	45.19	46.22	47.15	48.13	49.08	49.94	50.85
24	45.32	46.35	47.29	48.26	49.21	50.08	50.99
25	45.45	46.48	47.42	48.40	49.35	50.22	51.13
26	45.57	46.61	47.55	48.52	49.47	50.34	51.25
27	45.69	46.73	47.67	48.64	49.59	50.47	51.38
28	45.81	46.84	47.78	48.76	49.71	50.58	51.49
29	45.93	46.96	47.90	48.87	49.82	50.69	51.60
30	46.04	47.07	48.01	48.98	49.93	50.80	51.71
31	46.16	47.19	48.12	49.09	50.04	50.91	51.82
32	46.27	47.30	48.23	49.20	50.14	51.01	51.92
33	46.38	47.41	48.34	49.30	50.25	51.11	52.01
34	46.50	47.52	48.44	49.41	50.35	51.21	52.11
35	46.62	47.63	48.55	49.51	50.45	51.31	52.21
36	46.73	47.74	48.66	49.62	50.55	51.41	52.30

By Wuhan University Children’s Digital Health and Data Center (CDHDC)

The Postnatal Growth Reference for Preterm Infants

WUHAN UNIVERSITY

BMI (boys)

Corrected age(months)	Centiles						
	3rd	10rd	25rd	50rd	75rd	90rd	97rd
0	10.85	11.84	12.70	13.67	14.67	15.54	16.45
1	12.68	13.66	14.63	15.73	16.87	17.99	19.21
2	13.45	14.45	15.45	16.57	17.74	18.88	20.13
3	14.07	15.08	16.09	17.23	18.41	19.56	20.82
4	14.51	15.53	16.54	17.67	18.86	20.00	21.26
5	14.80	15.81	16.81	17.93	19.10	20.24	21.48
6	14.95	15.94	16.93	18.03	19.19	20.30	21.52
7	14.99	15.97	16.94	18.02	19.15	20.24	21.43
8	14.97	15.93	16.88	17.93	19.03	20.09	21.25
9	14.91	15.84	16.76	17.79	18.86	19.89	21.02
10	14.81	15.72	16.62	17.62	18.66	19.67	20.77
11	14.70	15.59	16.46	17.44	18.46	19.44	20.50
12	14.57	15.44	16.30	17.25	18.25	19.21	20.25
13	14.44	15.29	16.13	17.07	18.04	18.98	20.01
14	14.31	15.15	15.97	16.90	17.85	18.78	19.78
15	14.18	15.01	15.83	16.74	17.68	18.59	19.58
16	14.07	14.89	15.70	16.59	17.52	18.42	19.40
17	13.97	14.78	15.58	16.46	17.38	18.27	19.23
18	13.88	14.68	15.47	16.35	17.26	18.13	19.09
19	13.80	14.59	15.37	16.24	17.15	18.02	18.96
20	13.72	14.51	15.29	16.15	17.05	17.91	18.85
21	13.66	14.44	15.21	16.07	16.96	17.82	18.75
22	13.60	14.38	15.14	16.00	16.88	17.73	18.66
23	13.54	14.32	15.08	15.93	16.81	17.66	18.58
24	13.48	14.26	15.02	15.87	16.75	17.59	18.51
25	13.43	14.20	14.96	15.81	16.69	17.53	18.45
26	13.37	14.15	14.91	15.75	16.63	17.48	18.40
27	13.32	14.09	14.86	15.70	16.58	17.43	18.35
28	13.27	14.05	14.81	15.66	16.54	17.39	18.32
29	13.23	14.00	14.77	15.63	16.51	17.37	18.30
30	13.19	13.97	14.74	15.60	16.49	17.35	18.28
31	13.16	13.94	14.72	15.58	16.47	17.34	18.28
32	13.13	13.92	14.70	15.56	16.47	17.34	18.28
33	13.10	13.90	14.68	15.56	16.46	17.34	18.30
34	13.08	13.88	14.67	15.55	16.47	17.35	18.31
35	13.06	13.87	14.66	15.55	16.47	17.36	18.33
36	13.04	13.85	14.65	15.55	16.48	17.38	18.36

By Wuhan University Children's Digital Health and Data Center (CDHDC)

The Postnatal Growth Reference for Preterm Infants

Length (girls)

Corrected age(months)	Centiles						
	3rd	10rd	25rd	50rd	75rd	90rd	97rd
0	46.5	48.0	49.5	51.0	52.5	53.7	55.0
1	50.33	51.90	53.36	54.86	56.32	57.61	58.92
2	53.30	54.92	56.42	57.97	59.47	60.81	62.16
3	56.02	57.67	59.20	60.79	62.32	63.69	65.08
4	58.43	60.10	61.65	63.27	64.82	66.22	67.63
5	60.52	62.21	63.78	65.42	67.00	68.41	69.84
6	62.34	64.05	65.63	67.28	68.87	70.31	71.75
7	63.93	65.65	67.25	68.91	70.52	71.97	73.43
8	65.34	67.08	68.69	70.37	72.00	73.46	74.94
9	66.62	68.38	70.01	71.72	73.37	74.85	76.34
10	67.81	69.59	71.25	72.97	74.65	76.15	77.67
11	68.92	70.73	72.42	74.17	75.87	77.40	78.94
12	69.98	71.82	73.53	75.32	77.05	78.60	80.17
13	71.00	72.87	74.61	76.43	78.19	79.77	81.37
14	71.98	73.89	75.67	77.52	79.31	80.92	82.54
15	72.95	74.89	76.70	78.58	80.41	82.04	83.69
16	73.90	75.87	77.71	79.63	81.48	83.15	84.83
17	74.82	76.84	78.70	80.66	82.54	84.23	85.94
18	75.73	77.77	79.67	81.65	83.57	85.29	87.03
19	76.60	78.68	80.61	82.63	84.58	86.32	88.09
20	77.46	79.57	81.53	83.58	85.55	87.33	89.12
21	78.29	80.43	82.43	84.50	86.51	88.31	90.13
22	79.10	81.28	83.30	85.41	87.45	89.27	91.12
23	79.88	82.10	84.15	86.29	88.36	90.21	92.08
24	80.64	82.89	84.98	87.15	89.25	91.13	93.03
25	81.38	83.66	85.78	87.98	90.11	92.02	93.95
26	82.09	84.41	86.56	88.80	90.96	92.90	94.85
27	82.79	85.14	87.32	89.59	91.79	93.75	95.73
28	83.47	85.85	88.07	90.37	92.59	94.59	96.60
29	84.13	86.55	88.79	91.13	93.39	95.41	97.44
30	84.78	87.23	89.51	91.88	94.16	96.21	98.27
31	85.41	87.90	90.21	92.61	94.92	96.99	99.08
32	86.04	88.56	90.89	93.32	95.67	97.77	99.88
33	86.66	89.21	91.57	94.03	96.41	98.53	100.67
34	87.27	89.85	92.25	94.74	97.14	99.29	101.45
35	87.88	90.50	92.92	95.44	97.87	100.04	102.23
36	88.49	91.14	93.59	96.14	98.60	100.80	103.01

By Wuhan University Children’s Digital Health and Data Center (CDHDC)

The Postnatal Growth Reference for Preterm Infants

WUHAN UNIVERSITY

Weight (girls)

Corrected age(months)	Centiles						
	3rd	10rd	25rd	50rd	75rd	90rd	97rd
0	2.50	2.80	3.10	3.45	3.80	4.10	4.40
1	3.40	3.78	4.16	4.58	5.02	5.43	5.87
2	4.11	4.56	5.01	5.51	6.02	6.51	7.03
3	4.73	5.24	5.74	6.30	6.88	7.43	8.02
4	5.25	5.81	6.36	6.97	7.60	8.19	8.83
5	5.69	6.28	6.86	7.51	8.18	8.81	9.48
6	6.06	6.67	7.28	7.95	8.65	9.30	10.00
7	6.37	7.00	7.62	8.32	9.03	9.70	10.42
8	6.63	7.27	7.91	8.62	9.34	10.03	10.76
9	6.86	7.51	8.16	8.88	9.62	10.32	11.06
10	7.06	7.73	8.38	9.11	9.86	10.57	11.32
11	7.25	7.92	8.59	9.32	10.08	10.80	11.56
12	7.42	8.10	8.77	9.52	10.29	11.01	11.78
13	7.57	8.26	8.95	9.71	10.48	11.22	12.00
14	7.73	8.43	9.12	9.89	10.68	11.42	12.22
15	7.88	8.59	9.29	10.07	10.87	11.63	12.43
16	8.03	8.75	9.47	10.26	11.07	11.84	12.65
17	8.18	8.92	9.64	10.45	11.27	12.05	12.88
18	8.34	9.08	9.82	10.64	11.48	12.27	13.11
19	8.49	9.25	10.00	10.83	11.68	12.49	13.34
20	8.65	9.42	10.18	11.02	11.89	12.71	13.58
21	8.80	9.59	10.36	11.22	12.10	12.93	13.82
22	8.96	9.75	10.54	11.42	12.31	13.16	14.06
23	9.11	9.92	10.72	11.61	12.52	13.39	14.30
24	9.25	10.08	10.90	11.81	12.74	13.62	14.55
25	9.40	10.24	11.08	12.00	12.95	13.85	14.80
26	9.53	10.40	11.25	12.19	13.16	14.08	15.05
27	9.67	10.55	11.42	12.38	13.37	14.31	15.30
28	9.80	10.70	11.59	12.57	13.58	14.54	15.55
29	9.94	10.85	11.76	12.76	13.79	14.77	15.80
30	10.07	11.00	11.93	12.95	14.00	15.00	16.06
31	10.20	11.15	12.10	13.14	14.22	15.24	16.32
32	10.32	11.30	12.26	13.33	14.43	15.47	16.57
33	10.45	11.45	12.43	13.52	14.64	15.71	16.83
34	10.58	11.60	12.60	13.72	14.86	15.94	17.10
35	10.71	11.75	12.77	13.91	15.08	16.18	17.36
36	10.84	11.90	12.94	14.10	15.29	16.42	17.62

By Wuhan University Children's Digital Health and Data Center (CDHDC)

The Postnatal Growth Reference for Preterm Infants

WUHAN UNIVERSITY

Head circumference (girls)

Corrected age(months)	Centiles						
	3rd	10rd	25rd	50rd	75rd	90rd	97rd
0	32.3	33.1	34.0	35.0	35.8	36.5	37.0
1	34.44	35.30	36.08	36.89	37.69	38.42	39.19
2	35.81	36.68	37.47	38.29	39.10	39.85	40.63
3	37.04	37.92	38.73	39.56	40.38	41.13	41.92
4	38.12	39.01	39.82	40.66	41.49	42.25	43.05
5	39.05	39.94	40.76	41.61	42.44	43.21	44.02
6	39.83	40.73	41.55	42.41	43.24	44.02	44.83
7	40.48	41.38	42.21	43.07	43.91	44.69	45.52
8	41.02	41.93	42.76	43.63	44.48	45.26	46.09
9	41.48	42.40	43.23	44.10	44.96	45.75	46.58
10	41.87	42.79	43.63	44.51	45.36	46.16	46.99
11	42.20	43.13	43.97	44.85	45.71	46.51	47.35
12	42.49	43.42	44.27	45.15	46.02	46.82	47.67
13	42.74	43.68	44.53	45.42	46.29	47.10	47.94
14	42.97	43.91	44.77	45.66	46.53	47.34	48.19
15	43.18	44.12	44.98	45.88	46.75	47.56	48.42
16	43.38	44.32	45.18	46.08	46.96	47.77	48.63
17	43.57	44.51	45.37	46.27	47.15	47.96	48.82
18	43.75	44.69	45.55	46.45	47.33	48.14	49.00
19	43.92	44.86	45.72	46.62	47.50	48.31	49.17
20	44.09	45.03	45.89	46.78	47.66	48.47	49.33
21	44.25	45.19	46.04	46.94	47.82	48.63	49.48
22	44.40	45.34	46.20	47.09	47.97	48.78	49.63
23	44.55	45.49	46.35	47.24	48.11	48.92	49.78
24	44.70	45.63	46.49	47.38	48.25	49.06	49.92
25	44.83	45.77	46.63	47.52	48.39	49.20	50.05
26	44.97	45.90	46.76	47.65	48.52	49.33	50.19
27	45.10	46.03	46.89	47.78	48.65	49.46	50.31
28	45.22	46.15	47.01	47.90	48.78	49.59	50.44
29	45.34	46.27	47.13	48.02	48.90	49.71	50.56
30	45.45	46.39	47.25	48.14	49.02	49.83	50.69
31	45.56	46.50	47.36	48.26	49.14	49.95	50.81
32	45.67	46.62	47.48	48.38	49.26	50.07	50.93
33	45.78	46.73	47.59	48.49	49.38	50.19	51.06
34	45.89	46.84	47.71	48.61	49.50	50.32	51.18
35	46.00	46.95	47.82	48.73	49.62	50.44	51.31
36	46.11	47.07	47.94	48.85	49.74	50.56	51.43

By Wuhan University Children’s Digital Health and Data Center (CDHDC)

The Postnatal Growth Reference for Preterm Infants

WUHAN UNIVERSITY

BMI (girls)

Corrected age(months)	Centiles						
	3rd	10rd	25rd	50rd	75rd	90rd	97rd
0	10.8	11.6	12.4	13.3	14.1	15.0	15.9
1	12.30	13.20	14.10	15.12	16.20	17.25	18.42
2	13.04	13.98	14.92	15.97	17.09	18.18	19.39
3	13.64	14.60	15.56	16.65	17.79	18.91	20.14
4	14.07	15.05	16.03	17.12	18.28	19.40	20.65
5	14.36	15.34	16.32	17.41	18.57	19.69	20.93
6	14.52	15.49	16.46	17.55	18.69	19.80	21.03
7	14.58	15.54	16.49	17.56	18.68	19.77	20.97
8	14.57	15.51	16.44	17.49	18.58	19.65	20.82
9	14.51	15.43	16.34	17.36	18.43	19.47	20.61
10	14.41	15.31	16.20	17.20	18.24	19.25	20.37
11	14.30	15.18	16.04	17.02	18.03	19.02	20.11
12	14.17	15.03	15.88	16.83	17.83	18.79	19.85
13	14.04	14.88	15.72	16.65	17.62	18.57	19.61
14	13.91	14.74	15.56	16.47	17.43	18.36	19.38
15	13.80	14.61	15.42	16.32	17.26	18.17	19.17
16	13.69	14.49	15.29	16.17	17.10	18.00	18.99
17	13.60	14.39	15.17	16.05	16.96	17.85	18.82
18	13.52	14.30	15.07	15.94	16.84	17.72	18.68
19	13.45	14.22	14.99	15.85	16.74	17.61	18.56
20	13.39	14.16	14.92	15.76	16.65	17.51	18.45
21	13.34	14.10	14.85	15.70	16.57	17.43	18.36
22	13.29	14.05	14.80	15.64	16.51	17.35	18.28
23	13.25	14.01	14.75	15.58	16.45	17.29	18.22
24	13.21	13.96	14.71	15.54	16.40	17.24	18.16
25	13.17	13.92	14.66	15.49	16.35	17.19	18.11
26	13.13	13.88	14.62	15.45	16.31	17.15	18.06
27	13.09	13.84	14.58	15.41	16.27	17.11	18.03
28	13.05	13.80	14.55	15.37	16.24	17.08	17.99
29	13.02	13.77	14.51	15.34	16.21	17.05	17.97
30	12.99	13.74	14.49	15.32	16.19	17.03	17.96
31	12.96	13.72	14.47	15.30	16.18	17.02	17.95
32	12.94	13.70	14.45	15.29	16.17	17.02	17.95
33	12.92	13.68	14.44	15.28	16.17	17.02	17.96
34	12.90	13.67	14.43	15.28	16.17	17.03	17.98
35	12.88	13.66	14.42	15.28	16.18	17.05	18.00
36	12.87	13.65	14.42	15.28	16.19	17.06	18.03

By Wuhan University Children’s Digital Health and Data Center (CDHDC)
