# Supplementary material for: The growth diversity of preterm infants at 0–36 months corrected age in China: a real-world observational study
Source: Front Pediatr. 2025 Jan 31;13:1506244. doi: 10.3389/fped.2025.1506244 (PMC11825782; doi:10.3389/fped.2025.1506244)
Supplement: Supplementary file 2 [file Datasheet2.pdf]

The Postnatal Growth Reference for Term Infants

Length (boys)

Age (months)	Centiles						
	**3rd**	**10rd**	**25rd**	**50rd**	**75rd**	**90rd**	**97rd**
0	48.05	48.91	49.72	50.59	51.48	52.36	53.35
1	51.64	52.66	53.61	54.64	55.70	56.74	57.92
2	54.98	56.14	57.23	58.41	59.63	60.83	62.19
3	57.96	59.24	60.43	61.73	63.07	64.39	65.90
4	60.52	61.88	63.15	64.52	65.95	67.35	68.96
5	62.69	64.10	65.41	66.84	68.32	69.78	71.44
6	64.52	65.96	67.31	68.77	70.28	71.77	73.48
7	66.07	67.53	68.91	70.39	71.93	73.45	75.18
8	67.41	68.90	70.29	71.80	73.36	74.91	76.67
9	68.61	70.12	71.53	73.07	74.65	76.22	78.00
10	69.72	71.25	72.69	74.24	75.85	77.44	79.26
11	70.77	72.33	73.79	75.37	77.01	78.62	80.46
12	71.78	73.37	74.85	76.46	78.13	79.77	81.65
13	72.77	74.38	75.89	77.53	79.23	80.90	82.82
14	73.73	75.37	76.92	78.59	80.32	82.03	83.97
15	74.68	76.36	77.93	79.63	81.39	83.14	85.12
16	75.62	77.33	78.93	80.67	82.46	84.24	86.26
17	76.55	78.29	79.92	81.69	83.52	85.32	87.39
18	77.47	79.23	80.89	82.69	84.55	86.39	88.50
19	78.36	80.16	81.84	83.67	85.57	87.44	89.58
20	79.23	81.05	82.77	84.63	86.56	88.47	90.65
21	80.08	81.93	83.68	85.57	87.53	89.47	91.69
22	80.91	82.79	84.57	86.49	88.48	90.46	92.71
23	81.72	83.63	85.44	87.39	89.41	91.41	93.70
24	82.52	84.46	86.28	88.26	90.32	92.35	94.68
25	83.29	85.26	87.11	89.12	91.20	93.27	95.63
26	84.05	86.04	87.92	89.96	92.07	94.16	96.55
27	84.79	86.81	88.71	90.78	92.92	95.04	97.46
28	85.52	87.57	89.49	91.58	93.74	95.89	98.34
29	86.25	88.31	90.26	92.37	94.56	96.73	99.21
30	86.97	89.05	91.02	93.15	95.36	97.55	100.05
31	87.68	89.79	91.77	93.92	96.14	98.35	100.88
32	88.39	90.51	92.51	94.68	96.93	99.16	101.71
33	89.10	91.24	93.25	95.44	97.70	99.95	102.52
34	89.79	91.95	93.99	96.19	98.48	100.75	103.34
35	90.49	92.67	94.72	96.94	99.25	101.54	104.16
36	91.18	93.38	95.45	97.69	100.02	102.33	104.98

By Wuhan University Children's Digital Health and Data Center (CDHDC)

The Postnatal Growth Reference for Term Infants

Weight (boys)

Age (months)	Centiles						
	3rd	10rd	25rd	50rd	75rd	90rd	97rd
0	2.69	2.96	3.22	3.49	3.77	4.03	4.30
1	3.60	3.94	4.27	4.62	4.99	5.34	5.71
2	4.49	4.87	5.27	5.69	6.14	6.58	7.05
3	5.28	5.71	6.15	6.64	7.16	7.68	8.25
4	5.93	6.40	6.88	7.42	8.00	8.60	9.25
5	6.46	6.95	7.47	8.05	8.68	9.33	10.05
6	6.89	7.40	7.94	8.55	9.22	9.91	10.69
7	7.23	7.76	8.31	8.95	9.64	10.37	11.19
8	7.51	8.05	8.61	9.27	9.98	10.73	11.58
9	7.75	8.29	8.87	9.53	10.26	11.03	11.90
10	7.96	8.51	9.10	9.77	10.51	11.29	12.17
11	8.15	8.71	9.31	9.98	10.73	11.52	12.42
12	8.33	8.90	9.50	10.19	10.94	11.74	12.65
13	8.50	9.08	9.68	10.38	11.14	11.95	12.87
14	8.66	9.25	9.86	10.57	11.34	12.15	13.08
15	8.82	9.42	10.04	10.76	11.54	12.36	13.30
16	8.98	9.59	10.22	10.95	11.74	12.58	13.52
17	9.14	9.75	10.40	11.14	11.95	12.80	13.76
18	9.30	9.92	10.58	11.33	12.16	13.02	14.00
19	9.46	10.09	10.76	11.53	12.37	13.25	14.25
20	9.62	10.26	10.94	11.72	12.58	13.48	14.50
21	9.78	10.43	11.13	11.92	12.80	13.72	14.76
22	9.94	10.61	11.31	12.12	13.02	13.96	15.03
23	10.10	10.78	11.50	12.32	13.23	14.20	15.31
24	10.25	10.95	11.68	12.52	13.45	14.44	15.59
25	10.41	11.12	11.86	12.72	13.67	14.69	15.88
26	10.56	11.28	12.04	12.92	13.89	14.94	16.16
27	10.71	11.44	12.22	13.11	14.11	15.18	16.46
28	10.86	11.61	12.39	13.30	14.32	15.43	16.75
29	11.01	11.77	12.57	13.49	14.54	15.68	17.05
30	11.17	11.93	12.74	13.68	14.75	15.93	17.36
31	11.32	12.09	12.91	13.87	14.97	16.19	17.67
32	11.48	12.25	13.08	14.06	15.19	16.45	17.98
33	11.64	12.41	13.26	14.26	15.41	16.71	18.31
34	11.79	12.57	13.43	14.45	15.63	16.98	18.64
35	11.95	12.74	13.60	14.64	15.86	17.25	18.99
36	12.11	12.90	13.77	14.83	16.08	17.52	19.35

By Wuhan University Children's Digital Health and Data Center (CDHDC)

The Postnatal Growth Reference for Term Infants

Head circumference (boys)

Age (months)	Centiles						
	3rd	10rd	25rd	50rd	75rd	90rd	97rd
0	33.45	34.33	35.16	36.05	36.92	37.72	38.54
1	35.10	35.93	36.71	37.54	38.36	39.12	39.90
2	36.66	37.45	38.20	38.99	39.77	40.50	41.25
3	38.07	38.84	39.56	40.34	41.10	41.81	42.54
4	39.28	40.04	40.76	41.53	42.29	42.99	43.72
5	40.28	41.05	41.77	42.55	43.31	44.02	44.75
6	41.11	41.89	42.62	43.40	44.18	44.89	45.63
7	41.78	42.57	43.32	44.11	44.90	45.63	46.38
8	42.33	43.13	43.89	44.70	45.50	46.23	47.00
9	42.79	43.60	44.37	45.18	45.99	46.74	47.51
10	43.17	43.99	44.76	45.59	46.41	47.16	47.95
11	43.49	44.32	45.10	45.94	46.77	47.53	48.32
12	43.77	44.61	45.40	46.24	47.07	47.84	48.64
13	44.02	44.86	45.66	46.51	47.35	48.12	48.93
14	44.24	45.09	45.89	46.74	47.59	48.37	49.18
15	44.45	45.30	46.10	46.96	47.81	48.60	49.41
16	44.64	45.50	46.30	47.16	48.02	48.80	49.62
17	44.83	45.68	46.49	47.35	48.21	48.99	49.81
18	45.00	45.86	46.67	47.53	48.38	49.17	49.99
19	45.16	46.02	46.83	47.70	48.55	49.34	50.16
20	45.32	46.18	46.99	47.86	48.72	49.51	50.33
21	45.47	46.33	47.15	48.02	48.88	49.67	50.50
22	45.61	46.47	47.29	48.16	49.03	49.83	50.66
23	45.74	46.61	47.43	48.31	49.18	49.98	50.81
24	45.87	46.74	47.56	48.44	49.31	50.12	50.95
25	45.99	46.86	47.69	48.57	49.44	50.25	51.08
26	46.10	46.98	47.81	48.69	49.56	50.37	51.21
27	46.21	47.09	47.92	48.80	49.68	50.48	51.32
28	46.32	47.20	48.03	48.91	49.78	50.59	51.43
29	46.43	47.31	48.13	49.01	49.89	50.69	51.53
30	46.54	47.41	48.24	49.11	49.98	50.79	51.62
31	46.66	47.52	48.34	49.22	50.08	50.88	51.71
32	46.77	47.64	48.45	49.32	50.18	50.97	51.80
33	46.89	47.75	48.56	49.42	50.28	51.06	51.88
34	47.01	47.86	48.67	49.52	50.37	51.16	51.97
35	47.13	47.97	48.77	49.63	50.47	51.25	52.06
36	47.25	48.09	48.88	49.73	50.57	51.35	52.15

By Wuhan University Children's Digital Health and Data Center (CDHDC)

The Postnatal Growth Reference for Term Infants

BMI (boys)

Age (months)	Centiles						
	3rd	10rd	25rd	50rd	75rd	90rd	97rd
0	11.37	12.27	13.15	14.10	15.04	15.89	16.73
1	12.45	13.30	14.17	15.13	16.12	17.06	18.04
2	13.43	14.24	15.09	16.07	17.13	18.16	19.26
3	14.22	15.01	15.86	16.86	17.96	19.07	20.29
4	14.80	15.58	16.43	17.44	18.58	19.74	21.04
5	15.18	15.95	16.80	17.82	18.97	20.15	21.49
6	15.39	16.16	17.01	18.03	19.18	20.36	21.69
7	15.48	16.25	17.09	18.10	19.23	20.40	21.71
8	15.48	16.25	17.08	18.07	19.18	20.31	21.59
9	15.43	16.18	17.00	17.97	19.05	20.15	21.39
10	15.33	16.08	16.88	17.83	18.88	19.95	21.15
11	15.22	15.96	16.74	17.67	18.68	19.72	20.89
12	15.10	15.82	16.59	17.49	18.48	19.49	20.62
13	14.97	15.68	16.43	17.31	18.28	19.27	20.37
14	14.83	15.53	16.28	17.14	18.08	19.05	20.13
15	14.71	15.40	16.13	16.97	17.90	18.84	19.90
16	14.60	15.27	15.99	16.82	17.73	18.66	19.69
17	14.49	15.16	15.86	16.68	17.57	18.49	19.51
18	14.40	15.05	15.75	16.55	17.44	18.34	19.34
19	14.32	14.96	15.65	16.44	17.32	18.21	19.20
20	14.24	14.88	15.56	16.35	17.21	18.09	19.07
21	14.18	14.81	15.48	16.27	17.12	17.99	18.97
22	14.12	14.75	15.42	16.19	17.04	17.91	18.89
23	14.07	14.69	15.36	16.13	16.98	17.85	18.82
24	14.02	14.64	15.30	16.07	16.92	17.79	18.77
25	13.98	14.59	15.25	16.01	16.86	17.74	18.73
26	13.93	14.54	15.20	15.96	16.81	17.70	18.70
27	13.89	14.50	15.15	15.91	16.77	17.66	18.68
28	13.86	14.46	15.10	15.87	16.72	17.62	18.66
29	13.82	14.42	15.06	15.82	16.68	17.59	18.65
30	13.79	14.38	15.02	15.78	16.65	17.57	18.64
31	13.76	14.34	14.98	15.75	16.62	17.55	18.65
32	13.73	14.31	14.94	15.71	16.59	17.54	18.66
33	13.71	14.28	14.91	15.68	16.57	17.53	18.68
34	13.69	14.25	14.88	15.65	16.56	17.54	18.71
35	13.67	14.22	14.84	15.63	16.54	17.54	18.75
36	13.65	14.19	14.81	15.60	16.53	17.55	18.79

By Wuhan University Children’s Digital Health and Data Center (CDHDC)

The Postnatal Growth Reference for Term Infants

Length (girls)

Age (months)	Centiles						
	3rd	10rd	25rd	50rd	75rd	90rd	97rd
0	47.69	48.52	49.38	50.33	51.30	52.19	53.07
1	50.86	51.82	52.80	53.91	55.03	56.06	57.08
2	53.85	54.93	56.04	57.28	58.55	59.71	60.86
3	56.56	57.73	58.94	60.31	61.69	62.96	64.22
4	58.94	60.19	61.47	62.92	64.38	65.72	67.06
5	61.02	62.31	63.64	65.14	66.66	68.04	69.43
6	62.83	64.15	65.50	67.03	68.59	70.00	71.42
7	64.40	65.74	67.12	68.67	70.25	71.69	73.13
8	65.79	67.15	68.55	70.12	71.72	73.17	74.63
9	67.06	68.43	69.84	71.44	73.05	74.52	76.00
10	68.23	69.62	71.05	72.66	74.30	75.79	77.28
11	69.34	70.75	72.20	73.83	75.49	77.00	78.51
12	70.40	71.83	73.30	74.96	76.64	78.18	79.71
13	71.42	72.88	74.38	76.06	77.77	79.33	80.89
14	72.42	73.91	75.43	77.15	78.89	80.48	82.07
15	73.40	74.92	76.47	78.21	79.99	81.61	83.23
16	74.37	75.91	77.49	79.27	81.08	82.73	84.38
17	75.32	76.88	78.49	80.31	82.15	83.83	85.51
18	76.24	77.84	79.48	81.32	83.20	84.91	86.62
19	77.15	78.77	80.44	82.31	84.22	85.96	87.70
20	78.02	79.67	81.37	83.28	85.22	86.99	88.77
21	78.88	80.56	82.28	84.23	86.20	88.00	89.81
22	79.72	81.43	83.18	85.15	87.16	88.99	90.82
23	80.55	82.28	84.06	86.06	88.10	89.96	91.82
24	81.36	83.12	84.92	86.95	89.02	90.90	92.79
25	82.16	83.94	85.76	87.82	89.92	91.83	93.74
26	82.93	84.73	86.59	88.67	90.79	92.73	94.67
27	83.69	85.51	87.39	89.50	91.65	93.61	95.58
28	84.43	86.28	88.18	90.32	92.50	94.48	96.47
29	85.17	87.04	88.96	91.12	93.32	95.33	97.34
30	85.89	87.78	89.72	91.91	94.13	96.16	98.20
31	86.61	88.51	90.47	92.68	94.93	96.98	99.03
32	87.31	89.24	91.21	93.44	95.71	97.77	99.84
33	88.02	89.96	91.95	94.19	96.48	98.56	100.64
34	88.72	90.67	92.68	94.94	97.24	99.33	101.44
35	89.41	91.38	93.40	95.68	97.99	100.10	102.22
36	90.11	92.09	94.12	96.41	98.74	100.87	103.00

By Wuhan University Children’s Digital Health and Data Center (CDHDC)

The Postnatal Growth Reference for Term Infants

Weight (girls)

Age (months)	Centiles						
	3rd	10rd	25rd	50rd	75rd	90rd	97rd
0	2.66	2.88	3.10	3.36	3.64	3.93	4.25
1	3.46	3.73	4.01	4.34	4.70	5.06	5.47
2	4.22	4.55	4.89	5.28	5.70	6.13	6.62
3	4.91	5.29	5.67	6.12	6.61	7.10	7.65
4	5.49	5.91	6.34	6.84	7.38	7.92	8.54
5	5.96	6.42	6.89	7.43	8.01	8.61	9.28
6	6.35	6.84	7.33	7.91	8.53	9.16	9.88
7	6.67	7.17	7.70	8.30	8.96	9.61	10.36
8	6.93	7.46	8.00	8.62	9.30	9.98	10.75
9	7.16	7.70	8.25	8.89	9.59	10.28	11.08
10	7.36	7.92	8.48	9.13	9.83	10.54	11.35
11	7.55	8.11	8.69	9.35	10.06	10.78	11.59
12	7.73	8.30	8.88	9.55	10.27	10.99	11.81
13	7.90	8.48	9.07	9.74	10.47	11.20	12.03
14	8.07	8.65	9.25	9.93	10.67	11.41	12.25
15	8.23	8.82	9.42	10.12	10.87	11.62	12.47
16	8.39	8.99	9.60	10.31	11.07	11.83	12.69
17	8.55	9.16	9.78	10.50	11.27	12.04	12.92
18	8.71	9.33	9.96	10.69	11.48	12.26	13.15
19	8.87	9.50	10.14	10.89	11.69	12.49	13.39
20	9.03	9.67	10.33	11.08	11.90	12.71	13.64
21	9.18	9.84	10.51	11.28	12.11	12.95	13.89
22	9.34	10.01	10.69	11.48	12.33	13.18	14.15
23	9.49	10.18	10.88	11.68	12.55	13.42	14.41
24	9.64	10.35	11.06	11.89	12.78	13.67	14.68
25	9.79	10.51	11.24	12.09	13.00	13.91	14.95
26	9.94	10.67	11.42	12.29	13.22	14.16	15.22
27	10.09	10.84	11.60	12.49	13.45	14.41	15.50
28	10.23	11.00	11.78	12.69	13.67	14.65	15.77
29	10.38	11.16	11.97	12.89	13.89	14.90	16.05
30	10.53	11.33	12.15	13.09	14.12	15.15	16.32
31	10.68	11.50	12.33	13.29	14.34	15.39	16.58
32	10.84	11.67	12.51	13.49	14.56	15.63	16.84
33	10.99	11.84	12.70	13.69	14.77	15.86	17.10
34	11.15	12.01	12.88	13.89	14.99	16.09	17.35
35	11.31	12.18	13.07	14.09	15.20	16.33	17.60
36	11.47	12.35	13.25	14.29	15.42	16.55	17.85

By Wuhan University Children’s Digital Health and Data Center (CDHDC)

The Postnatal Growth Reference for Term Infants

WUHAN UNIVERSITY

Head circumference (girls)

Age (months)	Centiles						
	3rd	10rd	25rd	50rd	75rd	90rd	97rd
0	33.01	33.80	34.56	35.38	36.21	36.99	37.83
1	34.52	35.28	35.99	36.77	37.55	38.29	39.09
2	35.96	36.68	37.37	38.12	38.87	39.58	40.34
3	37.25	37.97	38.64	39.37	40.11	40.80	41.55
4	38.38	39.09	39.77	40.49	41.23	41.92	42.66
5	39.33	40.05	40.73	41.46	42.20	42.90	43.65
6	40.13	40.86	41.55	42.29	43.04	43.75	44.51
7	40.78	41.52	42.22	42.98	43.74	44.46	45.23
8	41.32	42.07	42.79	43.55	44.33	45.06	45.84
9	41.77	42.53	43.26	44.03	44.82	45.56	46.36
10	42.16	42.93	43.66	44.44	45.23	45.98	46.79
11	42.49	43.26	44.00	44.79	45.59	46.35	47.16
12	42.78	43.56	44.30	45.10	45.90	46.66	47.47
13	43.04	43.82	44.57	45.37	46.17	46.94	47.75
14	43.28	44.06	44.81	45.61	46.42	47.19	48.01
15	43.49	44.28	45.03	45.83	46.65	47.41	48.24
16	43.70	44.49	45.24	46.04	46.86	47.62	48.45
17	43.89	44.68	45.43	46.24	47.05	47.82	48.65
18	44.08	44.87	45.62	46.42	47.24	48.01	48.83
19	44.25	45.05	45.80	46.60	47.42	48.19	49.01
20	44.43	45.22	45.97	46.77	47.59	48.36	49.18
21	44.59	45.38	46.13	46.94	47.75	48.52	49.34
22	44.75	45.54	46.29	47.10	47.91	48.68	49.50
23	44.91	45.70	46.45	47.25	48.07	48.83	49.66
24	45.06	45.85	46.60	47.40	48.22	48.98	49.80
25	45.21	45.99	46.74	47.55	48.36	49.12	49.95
26	45.34	46.13	46.88	47.68	48.49	49.26	50.08
27	45.48	46.26	47.01	47.81	48.62	49.39	50.21
28	45.60	46.39	47.14	47.94	48.75	49.52	50.34
29	45.72	46.51	47.26	48.06	48.87	49.64	50.46
30	45.83	46.62	47.37	48.18	48.99	49.75	50.58
31	45.94	46.73	47.48	48.29	49.10	49.87	50.69
32	46.04	46.83	47.58	48.39	49.21	49.98	50.80
33	46.14	46.93	47.68	48.49	49.31	50.08	50.91
34	46.23	47.03	47.78	48.59	49.41	50.19	51.02
35	46.32	47.12	47.88	48.69	49.51	50.29	51.12
36	46.41	47.21	47.97	48.79	49.61	50.39	51.22

By Wuhan University Children’s Digital Health and Data Center (CDHDC)

The Postnatal Growth Reference for Term Infants

BMI (girls)

Age (months)	Centiles						
	3rd	10rd	25rd	50rd	75rd	90rd	97rd
0	11.09	11.98	12.85	13.78	14.68	15.50	16.32
1	12.16	12.98	13.81	14.73	15.69	16.59	17.53
2	13.11	13.88	14.69	15.62	16.62	17.61	18.68
3	13.87	14.62	15.42	16.37	17.42	18.49	19.67
4	14.43	15.16	15.97	16.93	18.02	19.14	20.41
5	14.80	15.53	16.33	17.32	18.43	19.57	20.88
6	15.00	15.74	16.55	17.54	18.65	19.81	21.11
7	15.08	15.82	16.63	17.62	18.73	19.87	21.16
8	15.06	15.81	16.62	17.60	18.70	19.82	21.08
9	14.99	15.74	16.55	17.51	18.58	19.68	20.91
10	14.89	15.64	16.43	17.38	18.42	19.49	20.69
11	14.78	15.51	16.30	17.22	18.24	19.28	20.45
12	14.66	15.38	16.15	17.05	18.04	19.06	20.21
13	14.54	15.24	15.99	16.87	17.84	18.84	19.97
14	14.42	15.10	15.84	16.70	17.65	18.63	19.74
15	14.30	14.97	15.69	16.54	17.47	18.44	19.52
16	14.20	14.85	15.56	16.39	17.31	18.26	19.33
17	14.11	14.75	15.44	16.25	17.16	18.10	19.16
18	14.03	14.66	15.33	16.13	17.03	17.95	19.01
19	13.96	14.58	15.24	16.03	16.91	17.83	18.87
20	13.90	14.51	15.16	15.94	16.81	17.72	18.75
21	13.84	14.44	15.09	15.86	16.72	17.62	18.65
22	13.79	14.38	15.03	15.79	16.65	17.54	18.57
23	13.74	14.33	14.97	15.73	16.58	17.48	18.51
24	13.69	14.28	14.92	15.68	16.53	17.42	18.46
25	13.64	14.23	14.87	15.63	16.48	17.38	18.43
26	13.59	14.19	14.83	15.58	16.43	17.34	18.41
27	13.55	14.15	14.79	15.54	16.40	17.32	18.40
28	13.51	14.11	14.76	15.51	16.37	17.30	18.40
29	13.48	14.09	14.73	15.49	16.34	17.28	18.42
30	13.46	14.06	14.71	15.46	16.32	17.28	18.44
31	13.45	14.05	14.69	15.45	16.31	17.27	18.46
32	13.44	14.04	14.68	15.43	16.29	17.27	18.48
33	13.43	14.03	14.67	15.42	16.28	17.27	18.51
34	13.42	14.02	14.66	15.41	16.27	17.27	18.53
35	13.42	14.01	14.65	15.39	16.26	17.26	18.55
36	13.41	14.01	14.64	15.38	16.25	17.26	18.58

By Wuhan University Children's Digital Health and Data Center (CDHDC)
