# Supplementary material for: The growth diversity of preterm infants at 0–36 months corrected age in China: a real-world observational study
Source: Front Pediatr. 2025 Jan 31;13:1506244. doi: 10.3389/fped.2025.1506244 (PMC11825782; doi:10.3389/fped.2025.1506244)
Supplement: Supplementary file 3 [file Datasheet3.pdf]

The Postnatal Growth Reference for Preterm Infants

WUHAN UNIVERSITY

Length (36w boys)

Corrected age(months)	Centiles						
	3rd	10rd	25rd	50rd	75rd	90rd	97rd
0	48.10	49.80	51.00	52.50	54.00	55.00	56.00
1	52.15	53.60	54.97	55.50	57.79	59.08	60.44
2	55.21	56.78	58.21	59.69	61.13	62.47	63.88
3	58.04	59.65	61.12	62.64	64.12	65.49	66.95
4	60.51	62.15	63.64	65.18	66.70	68.10	69.58
5	62.61	64.27	65.79	67.35	68.89	70.31	71.81
6	64.40	66.08	67.61	69.19	70.75	72.19	73.71
7	65.93	67.63	69.17	70.78	72.35	73.80	75.34
8	67.28	68.99	70.55	72.18	73.77	75.24	76.79
9	68.49	70.23	71.81	73.45	75.06	76.55	78.13
10	69.62	71.38	72.98	74.65	76.28	77.79	79.39
11	70.69	72.47	74.10	75.79	77.44	78.97	80.59
12	71.71	73.53	75.18	76.89	78.57	80.13	81.78
13	72.70	74.55	76.23	77.97	79.68	81.27	82.94
14	73.67	75.55	77.26	79.04	80.78	82.39	84.09
15	74.61	76.52	78.27	80.08	81.85	83.50	85.23
16	75.52	77.48	79.25	81.10	82.91	84.58	86.35
17	76.41	78.40	80.22	82.10	83.94	85.65	87.45
18	77.27	79.30	81.15	83.07	84.95	86.68	88.52
19	78.11	80.18	82.06	84.01	85.92	87.69	89.56
20	78.93	81.03	82.95	84.93	86.88	88.67	90.58
21	79.73	81.87	83.81	85.83	87.81	89.63	91.56
22	80.52	82.69	84.67	86.71	88.72	90.57	92.53
23	81.30	83.50	85.50	87.58	89.61	91.49	93.48
24	82.05	84.28	86.31	88.42	90.48	92.39	94.40
25	82.78	85.05	87.11	89.24	91.33	93.27	95.31
26	83.49	85.79	87.88	90.05	92.17	94.14	96.21
27	84.18	86.52	88.64	90.84	93.00	94.99	97.09
28	84.85	87.23	89.38	91.62	93.80	95.83	97.96
29	85.51	87.92	90.11	92.38	94.60	96.65	98.82
30	86.15	88.60	90.82	93.12	95.38	97.46	99.66
31	86.78	89.26	91.52	93.86	96.14	98.25	100.48
32	87.39	89.92	92.20	94.58	96.90	99.04	101.30
33	88.00	90.56	92.88	95.29	97.64	99.82	102.11
34	88.59	91.19	93.55	95.99	98.38	100.59	102.91
35	89.19	91.83	94.22	96.69	99.11	101.35	103.71
36	89.78	92.46	94.88	97.39	99.85	102.12	104.51

By Wuhan University Children’s Digital Health and Data Center (CDHDC)

The Postnatal Growth Reference for Preterm Infants

WUHAN UNIVERSITY

Weight (36w boys)

Corrected age(months)	Centiles						
	3rd	10rd	25rd	50rd	75rd	90rd	97rd
0	2.70	3.10	3.45	3.80	4.20	4.50	4.90
1	3.50	4.00	4.40	4.80	5.30	5.80	6.30
2	4.63	5.12	5.58	6.09	6.63	7.16	7.76
3	5.34	5.88	6.40	6.98	7.58	8.17	8.83
4	5.93	6.51	7.07	7.69	8.34	8.98	9.69
5	6.40	7.01	7.61	8.26	8.94	9.61	10.37
6	6.77	7.42	8.03	8.71	9.42	10.11	10.89
7	7.08	7.74	8.37	9.07	9.79	10.50	11.31
8	7.34	8.01	8.66	9.36	10.10	10.82	11.64
9	7.56	8.24	8.90	9.61	10.36	11.09	11.92
10	7.76	8.45	9.11	9.84	10.59	11.33	12.17
11	7.93	8.63	9.30	10.04	10.80	11.55	12.39
12	8.10	8.81	9.49	10.23	11.00	11.76	12.61
13	8.25	8.97	9.66	10.41	11.19	11.96	12.83
14	8.40	9.13	9.83	10.60	11.39	12.17	13.05
15	8.55	9.29	10.00	10.78	11.59	12.38	13.27
16	8.70	9.45	10.17	10.97	11.79	12.59	13.50
17	8.84	9.61	10.35	11.15	11.99	12.81	13.74
18	8.98	9.77	10.52	11.34	12.20	13.03	13.98
19	9.12	9.92	10.69	11.53	12.40	13.26	14.22
20	9.26	10.08	10.86	11.72	12.61	13.48	14.47
21	9.41	10.24	11.04	11.91	12.82	13.71	14.72
22	9.55	10.40	11.21	12.10	13.03	13.94	14.97
23	9.69	10.56	11.39	12.30	13.25	14.18	15.23
24	9.83	10.72	11.57	12.50	13.46	14.42	15.49
25	9.97	10.87	11.74	12.69	13.68	14.65	15.75
26	10.10	11.03	11.92	12.89	13.90	14.90	16.02
27	10.24	11.18	12.09	13.09	14.12	15.14	16.29
28	10.37	11.34	12.27	13.28	14.34	15.38	16.56
29	10.51	11.49	12.44	13.48	14.56	15.63	16.83
30	10.64	11.65	12.62	13.68	14.79	15.88	17.11
31	10.77	11.80	12.79	13.88	15.01	16.12	17.38
32	10.90	11.95	12.96	14.08	15.23	16.38	17.66
33	11.03	12.11	13.14	14.28	15.46	16.63	17.95
34	11.16	12.26	13.31	14.48	15.69	16.88	18.23
35	11.29	12.41	13.49	14.68	15.91	17.14	18.52
36	11.41	12.56	13.66	14.88	16.14	17.39	18.81

By Wuhan University Children's Digital Health and Data Center (CDHDC)

The Postnatal Growth Reference for Preterm Infants

WUHAN UNIVERSITY

Head circumference (36w boys)

Corrected age(months)	Centiles						
	3rd	10rd	25rd	50rd	75rd	90rd	97rd
0	33.38	34.20	35.00	36.00	36.70	37.40	38.00
1	35.50	36.31	37.06	37.84	38.62	39.34	40.11
2	36.92	37.76	38.52	39.32	40.12	40.85	41.64
3	38.21	39.06	39.84	40.66	41.46	42.22	43.02
4	39.32	40.19	40.98	41.81	42.63	43.39	44.21
5	40.27	41.14	41.95	42.78	43.61	44.39	45.22
6	41.05	41.93	42.75	43.59	44.43	45.22	46.05
7	41.70	42.59	43.41	44.26	45.11	45.90	46.75
8	42.23	43.13	43.96	44.82	45.67	46.47	47.32
9	42.68	43.58	44.41	45.28	46.14	46.95	47.80
10	43.05	43.96	44.80	45.67	46.54	47.35	48.21
11	43.37	44.29	45.13	46.01	46.88	47.69	48.56
12	43.64	44.56	45.41	46.30	47.18	48.00	48.87
13	43.88	44.81	45.66	46.55	47.44	48.26	49.14
14	44.09	45.02	45.88	46.78	47.67	48.50	49.39
15	44.27	45.22	46.08	46.99	47.88	48.72	49.61
16	44.45	45.40	46.27	47.18	48.08	48.92	49.82
17	44.61	45.56	46.44	47.35	48.26	49.10	50.00
18	44.76	45.72	46.60	47.52	48.42	49.27	50.18
19	44.91	45.87	46.75	47.67	48.59	49.44	50.35
20	45.05	46.02	46.90	47.83	48.74	49.60	50.51
21	45.20	46.17	47.05	47.98	48.89	49.75	50.66
22	45.34	46.31	47.20	48.12	49.04	49.90	50.81
23	45.49	46.45	47.34	48.27	49.18	50.04	50.95
24	45.62	46.59	47.48	48.40	49.32	50.17	51.09
25	45.76	46.73	47.61	48.53	49.45	50.31	51.22
26	45.89	46.86	47.74	48.66	49.58	50.43	51.34
27	46.02	46.98	47.86	48.78	49.70	50.55	51.46
28	46.14	47.10	47.98	48.90	49.81	50.67	51.57
29	46.27	47.22	48.10	49.02	49.93	50.78	51.68
30	46.39	47.34	48.21	49.13	50.03	50.88	51.78
31	46.50	47.45	48.32	49.23	50.14	50.98	51.88
32	46.61	47.56	48.43	49.34	50.23	51.07	51.97
33	46.73	47.67	48.53	49.44	50.33	51.17	52.06
34	46.84	47.78	48.63	49.53	50.42	51.26	52.15
35	46.95	47.88	48.74	49.63	50.52	51.35	52.23
36	47.06	47.99	48.84	49.73	50.61	51.43	52.32

By Wuhan University Children’s Digital Health and Data Center (CDHDC)

The Postnatal Growth Reference for Preterm Infants

WUHAN UNIVERSITY

BMI (36w boys)

Corrected age(months)	Centiles						
	3rd	10rd	25rd	50rd	75rd	90rd	97rd
0	11.21	12.00	12.88	13.81	14.78	15.60	16.42
1	12.89	13.81	14.72	15.76	16.86	17.95	19.17
2	13.66	14.61	15.56	16.64	17.77	18.90	20.16
3	14.28	15.25	16.22	17.32	18.48	19.63	20.92
4	14.72	15.70	16.68	17.79	18.96	20.12	21.41
5	15.00	15.98	16.96	18.07	19.24	20.39	21.68
6	15.14	16.12	17.09	18.18	19.34	20.48	21.75
7	15.18	16.14	17.10	18.18	19.31	20.43	21.67
8	15.15	16.10	17.03	18.08	19.19	20.28	21.50
9	15.08	16.00	16.91	17.94	19.02	20.08	21.26
10	14.97	15.87	16.76	17.76	18.81	19.85	21.00
11	14.85	15.72	16.59	17.57	18.59	19.60	20.71
12	14.71	15.57	16.42	17.37	18.37	19.35	20.44
13	14.58	15.42	16.25	17.18	18.15	19.11	20.17
14	14.45	15.27	16.08	17.00	17.95	18.89	19.93
15	14.32	15.13	15.93	16.83	17.77	18.69	19.72
16	14.21	15.01	15.80	16.68	17.61	18.52	19.52
17	14.11	14.90	15.68	16.55	17.47	18.36	19.35
18	14.02	14.80	15.57	16.43	17.34	18.22	19.21
19	13.94	14.71	15.47	16.33	17.23	18.11	19.08
20	13.86	14.63	15.39	16.24	17.13	18.00	18.97
21	13.80	14.56	15.32	16.16	17.05	17.91	18.88
22	13.74	14.50	15.25	16.09	16.98	17.84	18.80
23	13.68	14.44	15.19	16.03	16.91	17.78	18.73
24	13.63	14.39	15.14	15.98	16.86	17.72	18.68
25	13.58	14.34	15.09	15.93	16.81	17.68	18.63
26	13.54	14.30	15.05	15.89	16.77	17.63	18.59
27	13.50	14.26	15.01	15.85	16.74	17.60	18.56
28	13.46	14.22	14.98	15.82	16.71	17.57	18.54
29	13.43	14.19	14.95	15.79	16.68	17.55	18.52
30	13.40	14.17	14.93	15.77	16.66	17.54	18.50
31	13.38	14.15	14.91	15.76	16.65	17.53	18.50
32	13.37	14.14	14.90	15.75	16.65	17.52	18.50
33	13.35	14.13	14.89	15.74	16.64	17.52	18.50
34	13.35	14.12	14.89	15.74	16.65	17.53	18.51
35	13.34	14.12	14.88	15.75	16.65	17.54	18.52
36	13.34	14.11	14.88	15.75	16.66	17.55	18.54

By Wuhan University Children's Digital Health and Data Center (CDHDC)

The Postnatal Growth Reference for Preterm Infants

WUHAN UNIVERSITY

Length (35w boys)

Corrected age(months)	Centiles						
	3rd	10rd	25rd	50rd	75rd	90rd	97rd
0	47.70	49.40	50.90	52.30	53.80	55.00	56.50
1	51.67	53.31	54.77	56.26	57.71	59.04	60.46
2	54.90	56.57	58.06	59.58	61.06	62.43	63.87
3	57.81	59.50	61.00	62.54	64.04	65.43	66.90
4	60.33	62.03	63.54	65.10	66.61	68.01	69.49
5	62.48	64.18	65.70	67.26	68.78	70.19	71.68
6	64.30	66.00	67.53	69.09	70.62	72.03	73.53
7	65.85	67.56	69.09	70.67	72.20	73.63	75.14
8	67.21	68.94	70.48	72.07	73.61	75.05	76.57
9	68.44	70.18	71.74	73.35	74.91	76.37	77.91
10	69.57	71.34	72.92	74.55	76.14	77.62	79.18
11	70.62	72.43	74.04	75.70	77.32	78.82	80.42
12	71.63	73.47	75.12	76.82	78.47	80.00	81.63
13	72.60	74.49	76.17	77.90	79.59	81.16	82.82
14	73.55	75.48	77.20	78.97	80.70	82.30	83.99
15	74.48	76.45	78.21	80.02	81.79	83.42	85.15
16	75.39	77.40	79.20	81.05	82.86	84.52	86.30
17	76.28	78.34	80.18	82.06	83.90	85.61	87.41
18	77.16	79.25	81.13	83.05	84.93	86.66	88.50
19	78.01	80.15	82.06	84.02	85.93	87.69	89.57
20	78.85	81.03	82.97	84.96	86.90	88.70	90.61
21	79.68	81.89	83.86	85.89	87.86	89.68	91.62
22	80.50	82.74	84.74	86.80	88.79	90.64	92.60
23	81.30	83.57	85.60	87.68	89.70	91.58	93.57
24	82.08	84.38	86.44	88.54	90.59	92.49	94.50
25	82.85	85.17	87.25	89.38	91.45	93.37	95.41
26	83.60	85.95	88.05	90.20	92.29	94.23	96.29
27	84.33	86.70	88.82	91.00	93.11	95.07	97.14
28	85.06	87.45	89.59	91.78	93.91	95.88	97.97
29	85.79	88.19	90.34	92.55	94.69	96.68	98.78
30	86.51	88.93	91.09	93.31	95.47	97.46	99.58
31	87.23	89.66	91.83	94.06	96.23	98.23	100.36
32	87.96	90.39	92.57	94.80	96.98	98.99	101.12
33	88.68	91.12	93.30	95.54	97.72	99.73	101.87
34	89.41	91.85	94.03	96.27	98.45	100.47	102.62
35	90.14	92.58	94.76	97.00	99.19	101.21	103.35
36	90.86	93.31	95.49	97.73	99.92	101.94	104.09

By Wuhan University Children’s Digital Health and Data Center (CDHDC)

The Postnatal Growth Reference for Preterm Infants

WUHAN UNIVERSITY

Weight (35w boys)

Corrected age(months)	Centiles						
	3rd	10rd	25rd	50rd	75rd	90rd	97rd
0	2.82	3.15	3.48	3.84	4.22	4.58	4.98
1	3.73	4.15	4.57	5.03	5.51	5.97	6.48
2	4.55	5.05	5.53	6.07	6.62	7.16	7.75
3	5.26	5.81	6.35	6.94	7.55	8.14	8.79
4	5.86	6.45	7.02	7.65	8.29	8.92	9.61
5	6.34	6.95	7.55	8.20	8.88	9.53	10.25
6	6.73	7.36	7.97	8.64	9.34	10.00	10.74
7	7.04	7.69	8.31	8.99	9.70	10.38	11.12
8	7.31	7.96	8.59	9.29	10.00	10.69	11.44
9	7.54	8.20	8.84	9.54	10.26	10.96	11.72
10	7.75	8.42	9.07	9.77	10.50	11.20	11.97
11	7.94	8.62	9.27	9.99	10.72	11.43	12.20
12	8.12	8.81	9.47	10.19	10.93	11.65	12.43
13	8.29	8.99	9.65	10.39	11.14	11.86	12.66
14	8.46	9.16	9.84	10.58	11.35	12.08	12.89
15	8.62	9.33	10.02	10.78	11.55	12.30	13.12
16	8.77	9.50	10.20	10.97	11.76	12.52	13.36
17	8.93	9.67	10.39	11.17	11.97	12.75	13.60
18	9.08	9.84	10.57	11.36	12.18	12.97	13.84
19	9.23	10.00	10.74	11.56	12.39	13.20	14.08
20	9.38	10.17	10.92	11.75	12.60	13.42	14.32
21	9.53	10.33	11.10	11.95	12.82	13.65	14.57
22	9.68	10.49	11.28	12.14	13.03	13.88	14.82
23	9.82	10.65	11.46	12.34	13.24	14.11	15.07
24	9.96	10.81	11.63	12.53	13.45	14.34	15.32
25	10.10	10.97	11.80	12.72	13.67	14.58	15.57
26	10.23	11.12	11.97	12.91	13.88	14.81	15.83
27	10.35	11.26	12.14	13.11	14.10	15.05	16.10
28	10.48	11.41	12.31	13.30	14.31	15.30	16.37
29	10.60	11.55	12.48	13.49	14.54	15.54	16.65
30	10.72	11.70	12.65	13.69	14.76	15.80	16.94
31	10.83	11.84	12.82	13.89	14.99	16.06	17.23
32	10.94	11.98	12.99	14.09	15.23	16.33	17.53
33	11.06	12.12	13.16	14.29	15.46	16.60	17.84
34	11.16	12.26	13.33	14.50	15.70	16.87	18.15
35	11.27	12.40	13.50	14.70	15.94	17.15	18.47
36	11.38	12.54	13.67	14.90	16.19	17.42	18.79

By Wuhan University Children's Digital Health and Data Center (CDHDC)

The Postnatal Growth Reference for Preterm Infants

Head circumference (35w boys)

Corrected age(months)	Centiles						
	3rd	10rd	25rd	50rd	75rd	90rd	97rd
0	33.10	34.00	35.00	35.90	36.50	37.30	38.00
1	35.31	36.14	36.92	37.75	38.57	39.31	40.06
2	36.78	37.63	38.42	39.27	40.10	40.85	41.62
3	38.09	38.95	39.76	40.62	41.46	42.22	43.00
4	39.22	40.09	40.91	41.77	42.62	43.40	44.19
5	40.17	41.05	41.87	42.75	43.60	44.38	45.18
6	40.96	41.84	42.67	43.55	44.41	45.20	46.00
7	41.61	42.50	43.33	44.21	45.08	45.87	46.68
8	42.16	43.04	43.88	44.76	45.64	46.43	47.24
9	42.61	43.50	44.34	45.23	46.10	46.89	47.71
10	43.00	43.89	44.73	45.62	46.49	47.28	48.10
11	43.34	44.23	45.06	45.95	46.82	47.62	48.43
12	43.63	44.52	45.35	46.24	47.11	47.90	48.71
13	43.89	44.77	45.61	46.49	47.36	48.15	48.97
14	44.11	45.00	45.83	46.72	47.59	48.38	49.19
15	44.32	45.20	46.04	46.92	47.79	48.59	49.40
16	44.50	45.39	46.22	47.11	47.98	48.78	49.59
17	44.67	45.56	46.40	47.29	48.16	48.95	49.77
18	44.84	45.72	46.56	47.45	48.33	49.12	49.94
19	44.99	45.88	46.72	47.61	48.49	49.29	50.10
20	45.14	46.03	46.87	47.77	48.64	49.44	50.26
21	45.29	46.18	47.02	47.91	48.79	49.59	50.41
22	45.43	46.32	47.17	48.06	48.94	49.74	50.56
23	45.57	46.46	47.31	48.20	49.08	49.88	50.70
24	45.70	46.60	47.44	48.34	49.22	50.02	50.84
25	45.83	46.73	47.57	48.47	49.35	50.15	50.98
26	45.96	46.85	47.70	48.59	49.48	50.28	51.10
27	46.08	46.97	47.82	48.72	49.60	50.40	51.23
28	46.19	47.09	47.93	48.83	49.72	50.52	51.35
29	46.30	47.20	48.05	48.95	49.83	50.64	51.46
30	46.41	47.30	48.15	49.05	49.94	50.75	51.58
31	46.51	47.41	48.26	49.16	50.05	50.86	51.69
32	46.60	47.51	48.36	49.26	50.15	50.97	51.80
33	46.70	47.60	48.46	49.37	50.26	51.07	51.91
34	46.79	47.70	48.55	49.47	50.36	51.18	52.02
35	46.88	47.79	48.65	49.57	50.47	51.29	52.13
36	46.96	47.88	48.75	49.67	50.57	51.39	52.24

By Wuhan University Children's Digital Health and Data Center (CDHDC)

The Postnatal Growth Reference for Preterm Infants

WUHAN UNIVERSITY

BMI (35w boys)

Corrected age(months)	Centiles						
	3rd	10rd	25rd	50rd	75rd	90rd	97rd
0	11.77	12.64	13.52	14.51	15.55	16.57	17.69
1	12.78	13.72	14.67	15.74	16.87	17.97	19.18
2	13.60	14.56	15.53	16.63	17.78	18.90	20.13
3	14.25	15.22	16.20	17.31	18.47	19.60	20.83
4	14.71	15.68	16.66	17.77	18.92	20.04	21.27
5	14.99	15.96	16.93	18.02	19.17	20.27	21.48
6	15.14	16.09	17.04	18.12	19.24	20.32	21.50
7	15.17	16.11	17.04	18.09	19.19	20.25	21.40
8	15.14	16.06	16.97	17.99	19.06	20.10	21.22
9	15.06	15.96	16.85	17.85	18.89	19.90	20.99
10	14.96	15.83	16.70	17.68	18.70	19.68	20.74
11	14.84	15.70	16.55	17.50	18.49	19.45	20.49
12	14.72	15.56	16.39	17.32	18.29	19.23	20.24
13	14.60	15.42	16.24	17.15	18.10	19.01	20.01
14	14.49	15.29	16.09	16.99	17.92	18.82	19.79
15	14.38	15.17	15.96	16.84	17.76	18.64	19.59
16	14.28	15.06	15.84	16.70	17.61	18.47	19.41
17	14.19	14.96	15.73	16.58	17.47	18.32	19.25
18	14.11	14.87	15.62	16.47	17.35	18.19	19.10
19	14.03	14.78	15.53	16.37	17.23	18.07	18.97
20	13.95	14.70	15.44	16.27	17.13	17.96	18.86
21	13.89	14.63	15.36	16.19	17.04	17.87	18.75
22	13.82	14.56	15.29	16.11	16.96	17.78	18.67
23	13.76	14.49	15.22	16.04	16.89	17.70	18.59
24	13.69	14.43	15.16	15.98	16.82	17.64	18.52
25	13.63	14.37	15.10	15.92	16.77	17.58	18.47
26	13.57	14.31	15.04	15.86	16.72	17.54	18.43
27	13.51	14.25	14.99	15.82	16.68	17.50	18.40
28	13.46	14.21	14.95	15.78	16.64	17.48	18.38
29	13.41	14.16	14.91	15.75	16.62	17.46	18.37
30	13.36	14.12	14.88	15.73	16.61	17.46	18.38
31	13.32	14.09	14.85	15.71	16.60	17.46	18.39
32	13.29	14.06	14.83	15.70	16.60	17.47	18.42
33	13.25	14.04	14.82	15.70	16.61	17.49	18.45
34	13.22	14.01	14.81	15.69	16.62	17.52	18.49
35	13.19	13.99	14.79	15.70	16.64	17.55	18.54
36	13.15	13.97	14.79	15.70	16.66	17.58	18.59

By Wuhan University Children’s Digital Health and Data Center (CDHDC)

The Postnatal Growth Reference for Preterm Infants

WUHAN UNIVERSITY

Length(34w boys)

Corrected age(months)	Centiles						
	3rd	10rd	25rd	50rd	75rd	90rd	97rd
0	47.28	49.00	50.50	52.00	53.50	55.00	56.00
1	51.52	53.13	54.62	56.15	57.65	58.99	60.37
2	54.93	56.58	58.09	59.67	61.19	62.58	63.99
3	57.91	59.57	61.11	62.71	64.26	65.66	67.10
4	60.42	62.10	63.64	65.26	66.82	68.24	69.69
5	62.52	64.20	65.75	67.37	68.95	70.38	71.84
6	64.27	65.95	67.51	69.14	70.72	72.16	73.64
7	65.76	67.46	69.02	70.66	72.26	73.70	75.19
8	67.08	68.78	70.37	72.02	73.63	75.09	76.59
9	68.26	69.99	71.59	73.27	74.90	76.38	77.89
10	69.37	71.13	72.75	74.45	76.10	77.61	79.15
11	70.43	72.21	73.86	75.59	77.27	78.79	80.36
12	71.45	73.26	74.94	76.69	78.40	79.95	81.54
13	72.46	74.30	76.00	77.78	79.51	81.08	82.70
14	73.45	75.32	77.04	78.85	80.61	82.20	83.84
15	74.44	76.33	78.08	79.91	81.69	83.30	84.96
16	75.41	77.33	79.10	80.95	82.75	84.39	86.06
17	76.37	78.31	80.10	81.97	83.79	85.45	87.14
18	77.30	79.26	81.07	82.96	84.81	86.48	88.20
19	78.21	80.19	82.02	83.94	85.80	87.49	89.23
20	79.10	81.11	82.96	84.90	86.78	88.49	90.25
21	79.98	82.01	83.89	85.85	87.76	89.49	91.27
22	80.85	82.90	84.80	86.79	88.73	90.48	92.29
23	81.69	83.78	85.71	87.72	89.68	91.46	93.29
24	82.51	84.63	86.58	88.63	90.62	92.43	94.28
25	83.29	85.44	87.43	89.51	91.53	93.37	95.25
26	84.04	86.22	88.25	90.36	92.42	94.29	96.20
27	84.75	86.97	89.03	91.18	93.28	95.18	97.12
28	85.42	87.69	89.79	91.98	94.12	96.05	98.03
29	86.08	88.39	90.53	92.77	94.94	96.91	98.93
30	86.70	89.07	91.26	93.54	95.76	97.77	99.83
31	87.30	89.72	91.96	94.29	96.56	98.61	100.72
32	87.88	90.36	92.65	95.04	97.36	99.46	101.61
33	88.45	90.99	93.34	95.78	98.15	100.30	102.50
34	89.00	91.61	94.01	96.52	98.95	101.15	103.40
35	89.55	92.23	94.69	97.26	99.75	102.00	104.30
36	90.09	92.84	95.37	98.00	100.55	102.85	105.21

By Wuhan University Children's Digital Health and Data Center (CDHDC)

The Postnatal Growth Reference for Preterm Infants

WUHAN UNIVERSITY

Weight(34w boys)

Corrected age(months)	Centiles						
	3rd	10rd	25rd	50rd	75rd	90rd	97rd
0	2.60	2.98	3.32	3.75	4.15	4.50	4.91
1	3.66	4.10	4.53	5.00	5.49	5.95	6.44
2	4.49	5.00	5.49	6.04	6.60	7.13	7.70
3	5.21	5.77	6.32	6.92	7.53	8.12	8.73
4	5.81	6.40	6.99	7.63	8.28	8.90	9.55
5	6.29	6.91	7.52	8.18	8.86	9.50	10.18
6	6.68	7.31	7.94	8.62	9.32	9.97	10.67
7	6.99	7.64	8.27	8.97	9.68	10.35	11.06
8	7.25	7.91	8.55	9.26	9.98	10.66	11.37
9	7.47	8.14	8.79	9.51	10.24	10.92	11.65
10	7.67	8.35	9.01	9.73	10.47	11.17	11.91
11	7.85	8.54	9.21	9.95	10.70	11.40	12.15
12	8.01	8.71	9.40	10.15	10.91	11.63	12.39
13	8.17	8.89	9.58	10.35	11.12	11.86	12.63
14	8.33	9.05	9.76	10.54	11.33	12.08	12.87
15	8.48	9.22	9.95	10.74	11.55	12.31	13.11
16	8.63	9.39	10.13	10.94	11.76	12.53	13.35
17	8.79	9.56	10.31	11.13	11.97	12.76	13.60
18	8.94	9.73	10.49	11.33	12.18	12.99	13.84
19	9.09	9.89	10.67	11.53	12.40	13.22	14.09
20	9.24	10.06	10.85	11.73	12.61	13.45	14.34
21	9.39	10.22	11.04	11.93	12.83	13.68	14.59
22	9.54	10.39	11.22	12.12	13.05	13.92	14.84
23	9.68	10.55	11.39	12.32	13.27	14.16	15.10
24	9.82	10.71	11.57	12.52	13.48	14.39	15.36
25	9.95	10.86	11.74	12.71	13.70	14.63	15.62
26	10.08	11.01	11.91	12.90	13.91	14.87	15.88
27	10.20	11.15	12.07	13.09	14.13	15.11	16.14
28	10.31	11.29	12.24	13.28	14.34	15.35	16.41
29	10.42	11.42	12.40	13.47	14.57	15.60	16.69
30	10.52	11.55	12.56	13.66	14.79	15.86	16.99
31	10.61	11.67	12.71	13.86	15.02	16.12	17.29
32	10.70	11.80	12.87	14.05	15.25	16.39	17.60
33	10.78	11.91	13.02	14.24	15.49	16.67	17.91
34	10.86	12.03	13.18	14.44	15.73	16.94	18.24
35	10.93	12.14	13.33	14.63	15.97	17.23	18.56
36	11.00	12.25	13.48	14.83	16.21	17.51	18.90

By Wuhan University Children’s Digital Health and Data Center (CDHDC)

The Postnatal Growth Reference for Preterm Infants

WUHAN UNIVERSITY

Head circumference(34w boys)

Corrected age(months)	Centiles						
	3rd	10rd	25rd	50rd	75rd	90rd	97rd
0	33.00	34.00	35.00	35.70	36.50	37.10	38.00
1	35.23	36.09	36.89	37.73	38.56	39.33	40.15
2	36.73	37.60	38.41	39.26	40.10	40.89	41.72
3	38.07	38.95	39.76	40.62	41.47	42.27	43.11
4	39.22	40.10	40.92	41.79	42.65	43.45	44.29
5	40.18	41.07	41.89	42.76	43.63	44.43	45.28
6	40.97	41.86	42.69	43.56	44.43	45.24	46.10
7	41.61	42.51	43.34	44.22	45.10	45.91	46.77
8	42.13	43.04	43.88	44.76	45.64	46.46	47.33
9	42.56	43.47	44.32	45.21	46.10	46.93	47.80
10	42.92	43.84	44.69	45.59	46.49	47.32	48.20
11	43.23	44.15	45.01	45.92	46.82	47.66	48.54
12	43.49	44.42	45.29	46.20	47.11	47.95	48.85
13	43.72	44.66	45.53	46.45	47.37	48.22	49.12
14	43.92	44.87	45.75	46.68	47.60	48.46	49.37
15	44.10	45.06	45.95	46.89	47.82	48.68	49.60
16	44.27	45.24	46.13	47.08	48.02	48.89	49.82
17	44.43	45.40	46.31	47.27	48.21	49.10	50.03
18	44.58	45.56	46.48	47.44	48.40	49.29	50.24
19	44.72	45.72	46.64	47.61	48.58	49.48	50.43
20	44.86	45.87	46.80	47.78	48.75	49.66	50.62
21	45.00	46.01	46.95	47.94	48.92	49.83	50.80
22	45.14	46.16	47.10	48.09	49.08	49.99	50.96
23	45.28	46.30	47.24	48.24	49.23	50.15	51.12
24	45.41	46.43	47.37	48.37	49.36	50.29	51.26
25	45.53	46.55	47.50	48.50	49.50	50.42	51.40
26	45.65	46.67	47.62	48.63	49.62	50.55	51.53
27	45.76	46.79	47.74	48.74	49.74	50.67	51.65
28	45.87	46.90	47.85	48.86	49.86	50.79	51.77
29	45.98	47.01	47.96	48.97	49.97	50.90	51.89
30	46.09	47.12	48.08	49.09	50.09	51.02	52.01
31	46.20	47.23	48.19	49.20	50.20	51.14	52.13
32	46.30	47.34	48.30	49.31	50.32	51.25	52.24
33	46.41	47.45	48.41	49.43	50.43	51.37	52.36
34	46.52	47.56	48.53	49.54	50.55	51.49	52.49
35	46.63	47.67	48.64	49.66	50.67	51.61	52.61
36	46.75	47.79	48.76	49.78	50.79	51.74	52.74

By Wuhan University Children’s Digital Health and Data Center (CDHDC)

The Postnatal Growth Reference for Preterm Infants

WUHAN UNIVERSITY

BMI(34w boys)

Corrected age(months)	Centiles						
	3rd	10rd	25rd	50rd	75rd	90rd	97rd
0	10.96	11.97	12.81	13.82	14.83	15.82	16.79
1	12.62	13.60	14.60	15.72	16.89	18.01	19.20
2	13.46	14.45	15.46	16.60	17.78	18.90	20.10
3	14.12	15.13	16.13	17.27	18.45	19.57	20.76
4	14.60	15.60	16.59	17.72	18.88	19.99	21.17
5	14.90	15.88	16.86	17.97	19.11	20.20	21.35
6	15.06	16.01	16.98	18.05	19.17	20.23	21.35
7	15.11	16.04	16.98	18.02	19.11	20.13	21.22
8	15.09	15.99	16.90	17.92	18.97	19.97	21.02
9	15.02	15.90	16.78	17.77	18.79	19.76	20.78
10	14.92	15.78	16.64	17.60	18.59	19.53	20.52
11	14.81	15.65	16.49	17.43	18.39	19.31	20.28
12	14.69	15.51	16.33	17.25	18.20	19.10	20.05
13	14.56	15.37	16.18	17.09	18.02	18.91	19.84
14	14.44	15.24	16.04	16.94	17.86	18.73	19.65
15	14.33	15.12	15.91	16.80	17.71	18.57	19.48
16	14.22	15.00	15.79	16.67	17.57	18.42	19.33
17	14.12	14.90	15.68	16.55	17.44	18.29	19.18
18	14.04	14.81	15.58	16.44	17.32	18.16	19.05
19	13.96	14.73	15.49	16.34	17.22	18.05	18.93
20	13.89	14.65	15.41	16.25	17.12	17.94	18.81
21	13.83	14.58	15.33	16.17	17.03	17.85	18.71
22	13.77	14.51	15.26	16.09	16.94	17.75	18.61
23	13.71	14.45	15.19	16.01	16.86	17.66	18.51
24	13.65	14.38	15.12	15.94	16.78	17.58	18.42
25	13.59	14.32	15.05	15.87	16.70	17.50	18.34
26	13.53	14.26	14.99	15.80	16.63	17.42	18.26
27	13.48	14.21	14.93	15.74	16.57	17.36	18.19
28	13.43	14.15	14.88	15.69	16.52	17.30	18.14
29	13.38	14.11	14.83	15.64	16.48	17.26	18.10
30	13.34	14.07	14.80	15.61	16.45	17.24	18.07
31	13.31	14.04	14.77	15.58	16.42	17.22	18.06
32	13.27	14.01	14.74	15.56	16.41	17.21	18.05
33	13.24	13.98	14.72	15.54	16.39	17.20	18.05
34	13.21	13.96	14.70	15.53	16.38	17.19	18.05
35	13.19	13.93	14.68	15.51	16.37	17.19	18.05
36	13.16	13.91	14.66	15.50	16.36	17.18	18.05

By Wuhan University Children's Digital Health and Data Center (CDHDC)

The Postnatal Growth Reference for Preterm Infants

WUHAN UNIVERSITY

Length (33w boys)

Corrected age(months)	Centiles						
	3rd	10rd	25rd	50rd	75rd	90rd	97rd
0	47.00	48.30	49.90	51.50	53.00	54.50	55.53
1	51.17	52.82	54.37	56.02	57.61	59.03	60.44
2	54.45	56.11	57.68	59.34	60.96	62.39	63.83
3	57.40	59.06	60.64	62.32	63.94	65.39	66.83
4	59.96	61.62	63.20	64.88	66.51	67.96	69.41
5	62.12	63.78	65.36	67.04	68.67	70.13	71.59
6	63.94	65.60	67.18	68.86	70.50	71.96	73.42
7	65.48	67.15	68.74	70.43	72.07	73.54	75.01
8	66.82	68.50	70.10	71.80	73.46	74.95	76.43
9	68.00	69.71	71.33	73.06	74.74	76.25	77.75
10	69.10	70.83	72.49	74.24	75.96	77.49	79.02
11	70.13	71.91	73.59	75.39	77.13	78.70	80.26
12	71.13	72.94	74.67	76.50	78.29	79.89	81.48
13	72.09	73.95	75.72	77.60	79.43	81.06	82.70
14	73.04	74.94	76.75	78.67	80.54	82.22	83.89
15	73.97	75.91	77.76	79.73	81.64	83.35	85.06
16	74.89	76.88	78.77	80.77	82.72	84.47	86.21
17	75.80	77.82	79.75	81.79	83.78	85.56	87.33
18	76.69	78.75	80.71	82.79	84.81	86.62	88.43
19	77.56	79.65	81.65	83.77	85.82	87.66	89.50
20	78.40	80.54	82.57	84.72	86.81	88.69	90.55
21	79.22	81.39	83.46	85.65	87.78	89.69	91.59
22	80.02	82.23	84.34	86.57	88.74	90.67	92.61
23	80.80	83.05	85.19	87.46	89.67	91.64	93.61
24	81.56	83.86	86.04	88.35	90.59	92.59	94.59
25	82.31	84.65	86.86	89.21	91.49	93.53	95.56
26	83.04	85.42	87.67	90.06	92.38	94.45	96.52
27	83.75	86.17	88.47	90.90	93.26	95.37	97.47
28	84.43	86.89	89.23	91.71	94.12	96.26	98.40
29	85.07	87.59	89.98	92.51	94.96	97.15	99.33
30	85.68	88.26	90.70	93.28	95.79	98.02	100.24
31	86.25	88.89	91.39	94.04	96.60	98.88	101.15
32	86.80	89.50	92.06	94.77	97.40	99.73	102.06
33	87.31	90.09	92.72	95.49	98.18	100.58	102.96
34	87.80	90.66	93.36	96.21	98.97	101.42	103.86
35	88.28	91.22	93.99	96.92	99.75	102.26	104.76
36	88.76	91.78	94.62	97.63	100.53	103.11	105.67

The Postnatal Growth Reference for Preterm Infants

WUHAN UNIVERSITY

Weight (33w boys)

Corrected age(months)	Centiles						
	3rd	10rd	25rd	50rd	75rd	90rd	97rd
0	2.60	2.90	3.20	3.55	4.00	4.40	4.80
1	3.58	4.03	4.48	4.96	5.46	5.94	6.45
2	4.40	4.92	5.43	5.99	6.56	7.10	7.69
3	5.11	5.68	6.24	6.85	7.47	8.06	8.70
4	5.70	6.30	6.89	7.54	8.20	8.83	9.50
5	6.17	6.80	7.41	8.09	8.77	9.42	10.12
6	6.55	7.19	7.82	8.51	9.22	9.89	10.60
7	6.85	7.51	8.15	8.86	9.57	10.25	10.98
8	7.09	7.76	8.42	9.13	9.87	10.56	11.30
9	7.29	7.98	8.64	9.37	10.12	10.82	11.58
10	7.47	8.17	8.85	9.59	10.35	11.07	11.84
11	7.63	8.34	9.04	9.80	10.58	11.32	12.10
12	7.78	8.51	9.23	10.01	10.80	11.56	12.37
13	7.93	8.68	9.41	10.21	11.02	11.80	12.62
14	8.08	8.84	9.59	10.41	11.24	12.03	12.87
15	8.23	9.01	9.77	10.61	11.45	12.26	13.12
16	8.39	9.18	9.96	10.81	11.67	12.49	13.36
17	8.55	9.36	10.14	11.00	11.88	12.72	13.60
18	8.70	9.53	10.33	11.20	12.10	12.94	13.84
19	8.86	9.70	10.51	11.40	12.31	13.17	14.08
20	9.02	9.87	10.69	11.60	12.52	13.39	14.32
21	9.17	10.04	10.87	11.79	12.73	13.62	14.56
22	9.32	10.20	11.05	11.99	12.94	13.84	14.81
23	9.47	10.36	11.23	12.18	13.15	14.07	15.05
24	9.61	10.52	11.41	12.38	13.37	14.30	15.30
25	9.75	10.68	11.58	12.57	13.58	14.54	15.56
26	9.88	10.83	11.76	12.77	13.80	14.78	15.83
27	10.00	10.98	11.92	12.96	14.03	15.03	16.10
28	10.11	11.11	12.09	13.16	14.25	15.29	16.39
29	10.21	11.24	12.25	13.35	14.48	15.55	16.69
30	10.30	11.36	12.40	13.55	14.71	15.82	17.00
31	10.37	11.48	12.55	13.74	14.94	16.09	17.31
32	10.44	11.58	12.70	13.93	15.18	16.37	17.64
33	10.49	11.68	12.84	14.11	15.41	16.65	17.97
34	10.54	11.77	12.97	14.30	15.65	16.94	18.32
35	10.58	11.86	13.11	14.48	15.89	17.23	18.66
36	10.61	11.94	13.24	14.67	16.13	17.52	19.02

By Wuhan University Children’s Digital Health and Data Center (CDHDC)

The Postnatal Growth Reference for Preterm Infants

WUHAN UNIVERSITY

Head circumference (33w boys)

Corrected age(months)	Centiles						
	3rd	10rd	25rd	50rd	75rd	90rd	97rd
0	33.00	33.60	34.50	35.50	36.20	37.00	37.65
1	35.07	36.01	36.86	37.72	38.55	39.32	40.11
2	36.55	37.50	38.36	39.23	40.07	40.84	41.64
3	37.87	38.83	39.69	40.56	41.41	42.18	43.00
4	39.01	39.96	40.82	41.70	42.56	43.34	44.16
5	39.95	40.91	41.78	42.66	43.52	44.30	45.13
6	40.73	41.70	42.56	43.45	44.31	45.11	45.93
7	41.37	42.34	43.21	44.11	44.98	45.77	46.61
8	41.90	42.87	43.75	44.66	45.53	46.33	47.18
9	42.34	43.32	44.21	45.12	46.00	46.81	47.66
10	42.71	43.71	44.60	45.52	46.41	47.22	48.08
11	43.03	44.04	44.94	45.86	46.76	47.58	48.44
12	43.31	44.32	45.23	46.16	47.06	47.89	48.76
13	43.55	44.57	45.48	46.42	47.33	48.17	49.04
14	43.76	44.78	45.71	46.65	47.57	48.41	49.29
15	43.95	44.98	45.91	46.86	47.78	48.63	49.51
16	44.12	45.16	46.09	47.05	47.97	48.83	49.72
17	44.28	45.32	46.26	47.22	48.15	49.01	49.91
18	44.43	45.48	46.42	47.39	48.32	49.18	50.08
19	44.57	45.63	46.57	47.55	48.49	49.35	50.26
20	44.71	45.77	46.72	47.70	48.64	49.51	50.42
21	44.84	45.90	46.86	47.84	48.79	49.67	50.58
22	44.96	46.03	46.99	47.98	48.94	49.82	50.73
23	45.08	46.16	47.12	48.11	49.07	49.96	50.88
24	45.19	46.27	47.24	48.24	49.21	50.09	51.02
25	45.29	46.38	47.36	48.36	49.33	50.22	51.15
26	45.39	46.49	47.47	48.48	49.46	50.35	51.29
27	45.49	46.59	47.58	48.59	49.58	50.48	51.42
28	45.57	46.68	47.68	48.70	49.70	50.60	51.56
29	45.65	46.77	47.78	48.81	49.81	50.73	51.69
30	45.72	46.86	47.88	48.92	49.93	50.85	51.82
31	45.79	46.94	47.97	49.02	50.04	50.97	51.95
32	45.85	47.01	48.05	49.12	50.15	51.09	52.08
33	45.91	47.08	48.14	49.21	50.26	51.21	52.21
34	45.97	47.16	48.22	49.31	50.36	51.33	52.34
35	46.02	47.23	48.30	49.41	50.47	51.45	52.46
36	46.08	47.30	48.39	49.50	50.58	51.56	52.59

By Wuhan University Children’s Digital Health and Data Center (CDHDC)

The Postnatal Growth Reference for Preterm Infants

BMI (33w boys)

Corrected age(months)	Centiles						
	3rd	10rd	25rd	50rd	75rd	90rd	97rd
0	10.85	11.70	12.58	13.51	14.58	15.39	16.42
1	12.66	13.66	14.66	15.78	16.97	18.13	19.41
2	13.47	14.48	15.48	16.60	17.79	18.95	20.22
3	14.11	15.12	16.11	17.23	18.41	19.55	20.82
4	14.56	15.56	16.54	17.65	18.81	19.94	21.18
5	14.83	15.82	16.79	17.87	19.01	20.12	21.33
6	14.97	15.93	16.88	17.95	19.06	20.14	21.32
7	15.00	15.94	16.87	17.91	18.99	20.04	21.20
8	14.95	15.87	16.78	17.79	18.85	19.87	21.00
9	14.85	15.75	16.64	17.63	18.67	19.67	20.77
10	14.72	15.61	16.48	17.46	18.47	19.46	20.53
11	14.58	15.46	16.32	17.28	18.28	19.25	20.31
12	14.44	15.31	16.16	17.11	18.09	19.05	20.10
13	14.31	15.16	16.00	16.94	17.92	18.86	19.90
14	14.18	15.03	15.86	16.79	17.75	18.69	19.71
15	14.07	14.90	15.73	16.64	17.60	18.53	19.54
16	13.97	14.80	15.61	16.52	17.46	18.38	19.38
17	13.88	14.70	15.51	16.40	17.34	18.24	19.23
18	13.80	14.61	15.41	16.30	17.22	18.12	19.10
19	13.73	14.54	15.33	16.21	17.12	18.01	18.98
20	13.67	14.46	15.25	16.12	17.03	17.91	18.87
21	13.61	14.40	15.18	16.04	16.94	17.81	18.77
22	13.55	14.34	15.11	15.97	16.86	17.73	18.67
23	13.50	14.28	15.04	15.90	16.78	17.64	18.58
24	13.44	14.22	14.98	15.83	16.71	17.57	18.50
25	13.38	14.16	14.92	15.76	16.65	17.50	18.43
26	13.32	14.10	14.86	15.70	16.59	17.44	18.37
27	13.26	14.04	14.80	15.65	16.53	17.39	18.33
28	13.20	13.98	14.75	15.60	16.49	17.35	18.29
29	13.14	13.93	14.70	15.56	16.45	17.32	18.27
30	13.09	13.88	14.66	15.52	16.43	17.30	18.26
31	13.04	13.83	14.62	15.50	16.41	17.30	18.27
32	12.98	13.79	14.58	15.47	16.39	17.29	18.28
33	12.93	13.74	14.55	15.45	16.38	17.29	18.29
34	12.88	13.70	14.51	15.42	16.37	17.30	18.31
35	12.82	13.65	14.48	15.40	16.36	17.30	18.33
36	12.76	13.61	14.44	15.38	16.35	17.31	18.35

By Wuhan University Children’s Digital Health and Data Center (CDHDC)

The Postnatal Growth Reference for Preterm Infants

WUHAN UNIVERSITY

Length (32w boys)

Corrected age(months)	Centiles						
	3rd	10rd	25rd	50rd	75rd	90rd	97rd
0	45.89	47.61	49.80	51.40	53.00	54.30	56.00
1	50.51	52.41	54.14	55.92	57.62	59.12	60.61
2	53.77	55.67	57.40	59.19	60.89	62.40	63.91
3	56.74	58.62	60.35	62.14	63.84	65.36	66.87
4	59.34	61.21	62.93	64.71	66.41	67.93	69.44
5	61.56	63.42	65.13	66.90	68.60	70.12	71.64
6	63.46	65.30	67.01	68.78	70.48	72.00	73.52
7	65.09	66.93	68.63	70.41	72.11	73.63	75.15
8	66.52	68.36	70.07	71.85	73.55	75.08	76.61
9	67.81	69.66	71.37	73.16	74.88	76.41	77.95
10	69.00	70.86	72.59	74.39	76.12	77.66	79.21
11	70.11	72.00	73.74	75.55	77.30	78.86	80.43
12	71.16	73.07	74.83	76.67	78.44	80.02	81.61
13	72.16	74.09	75.89	77.75	79.55	81.15	82.77
14	73.13	75.09	76.91	78.80	80.63	82.26	83.89
15	74.07	76.06	77.91	79.83	81.68	83.34	85.00
16	75.00	77.02	78.89	80.84	82.72	84.39	86.08
17	75.92	77.96	79.86	81.83	83.73	85.43	87.13
18	76.83	78.90	80.81	82.81	84.73	86.44	88.17
19	77.73	79.82	81.76	83.77	85.72	87.45	89.19
20	78.62	80.74	82.70	84.73	86.70	88.45	90.21
21	79.50	81.64	83.62	85.68	87.67	89.44	91.22
22	80.35	82.52	84.52	86.61	88.62	90.42	92.22
23	81.18	83.37	85.40	87.52	89.55	91.37	93.20
24	81.97	84.19	86.25	88.39	90.46	92.30	94.15
25	82.74	84.99	87.07	89.24	91.33	93.20	95.07
26	83.48	85.76	87.87	90.06	92.18	94.07	95.96
27	84.19	86.50	88.64	90.87	93.01	94.92	96.84
28	84.89	87.23	89.40	91.65	93.82	95.76	97.70
29	85.56	87.94	90.14	92.42	94.63	96.59	98.56
30	86.21	88.63	90.87	93.19	95.42	97.42	99.42
31	86.86	89.31	91.59	93.95	96.21	98.24	100.27
32	87.50	89.99	92.30	94.70	97.00	99.06	101.12
33	88.13	90.67	93.01	95.44	97.78	99.87	101.96
34	88.77	91.34	93.72	96.19	98.56	100.67	102.79
35	89.40	92.02	94.43	96.93	99.34	101.48	103.63
36	90.04	92.69	95.14	97.67	100.11	102.29	104.46

By Wuhan University Children's Digital Health and Data Center (CDHDC)

The Postnatal Growth Reference for Preterm Infants

WUHAN UNIVERSITY

Weight(32w boys)

Corrected age(months)	Centiles						
	3rd	10rd	25rd	50rd	75rd	90rd	97rd
0	2.40	2.79	3.15	3.60	4.05	4.50	4.90
1	3.46	3.96	4.44	4.96	5.48	5.97	6.48
2	4.23	4.81	5.36	5.96	6.56	7.12	7.72
3	4.91	5.55	6.16	6.82	7.48	8.10	8.75
4	5.49	6.17	6.82	7.52	8.22	8.88	9.58
5	5.97	6.67	7.35	8.08	8.81	9.50	10.22
6	6.36	7.09	7.78	8.53	9.28	9.99	10.73
7	6.69	7.43	8.13	8.90	9.66	10.38	11.14
8	6.97	7.72	8.43	9.21	9.98	10.71	11.47
9	7.22	7.97	8.70	9.48	10.26	10.99	11.76
10	7.44	8.20	8.93	9.72	10.51	11.25	12.03
11	7.64	8.41	9.15	9.95	10.75	11.49	12.28
12	7.83	8.60	9.35	10.16	10.97	11.72	12.52
13	8.00	8.79	9.54	10.36	11.18	11.94	12.75
14	8.16	8.96	9.72	10.55	11.38	12.16	12.97
15	8.32	9.13	9.90	10.74	11.58	12.37	13.19
16	8.48	9.30	10.08	10.93	11.78	12.58	13.41
17	8.64	9.47	10.26	11.12	11.98	12.79	13.64
18	8.80	9.64	10.45	11.32	12.19	13.01	13.86
19	8.97	9.82	10.63	11.51	12.40	13.23	14.10
20	9.13	10.00	10.82	11.71	12.61	13.45	14.33
21	9.30	10.17	11.01	11.91	12.82	13.67	14.56
22	9.46	10.35	11.19	12.11	13.03	13.89	14.79
23	9.62	10.52	11.37	12.30	13.23	14.10	15.02
24	9.77	10.68	11.55	12.48	13.43	14.30	15.23
25	9.92	10.84	11.72	12.66	13.61	14.50	15.44
26	10.07	10.99	11.88	12.84	13.80	14.70	15.64
27	10.21	11.15	12.04	13.01	13.98	14.89	15.85
28	10.35	11.30	12.21	13.19	14.17	15.09	16.06
29	10.50	11.45	12.37	13.36	14.36	15.29	16.27
30	10.64	11.61	12.54	13.54	14.55	15.49	16.48
31	10.79	11.77	12.71	13.72	14.74	15.70	16.70
32	10.94	11.93	12.88	13.91	14.94	15.91	16.92
33	11.10	12.10	13.06	14.10	15.14	16.11	17.14
34	11.26	12.27	13.24	14.29	15.34	16.32	17.35
35	11.43	12.45	13.42	14.48	15.53	16.52	17.56
36	11.60	12.62	13.60	14.66	15.73	16.72	17.77

By Wuhan University Children’s Digital Health and Data Center (CDHDC)

The Postnatal Growth Reference for Preterm Infants

WUHAN UNIVERSITY

Head circumference(32w boys)

Corrected age(months)	Centiles						
	3rd	10rd	25rd	50rd	75rd	90rd	97rd
0	32.52	33.50	34.50	35.50	36.40	37.00	37.80
1	34.87	35.88	36.77	37.66	38.49	39.24	40.00
2	36.36	37.38	38.27	39.16	40.00	40.76	41.52
3	37.71	38.72	39.61	40.50	41.35	42.11	42.89
4	38.86	39.88	40.77	41.66	42.51	43.28	44.06
5	39.83	40.84	41.73	42.63	43.49	44.26	45.04
6	40.61	41.63	42.53	43.44	44.30	45.07	45.86
7	41.25	42.28	43.18	44.10	44.97	45.75	46.55
8	41.77	42.81	43.73	44.65	45.53	46.32	47.13
9	42.20	43.25	44.18	45.11	46.00	46.80	47.61
10	42.56	43.62	44.56	45.50	46.40	47.21	48.03
11	42.86	43.93	44.88	45.83	46.74	47.56	48.39
12	43.11	44.20	45.16	46.12	47.04	47.86	48.70
13	43.34	44.43	45.40	46.37	47.30	48.13	48.98
14	43.54	44.65	45.62	46.60	47.53	48.37	49.22
15	43.73	44.84	45.82	46.81	47.75	48.59	49.45
16	43.92	45.03	46.02	47.01	47.95	48.80	49.66
17	44.09	45.22	46.20	47.20	48.15	49.00	49.86
18	44.26	45.39	46.38	47.38	48.33	49.19	50.06
19	44.43	45.56	46.56	47.56	48.52	49.37	50.25
20	44.60	45.73	46.73	47.74	48.70	49.56	50.44
21	44.76	45.90	46.91	47.92	48.88	49.74	50.62
22	44.92	46.07	47.07	48.09	49.05	49.92	50.80
23	45.08	46.22	47.24	48.25	49.22	50.09	50.98
24	45.23	46.38	47.39	48.41	49.38	50.25	51.14
25	45.38	46.53	47.54	48.56	49.53	50.40	51.30
26	45.53	46.68	47.69	48.71	49.68	50.55	51.44
27	45.68	46.82	47.83	48.85	49.81	50.68	51.57
28	45.82	46.96	47.97	48.98	49.94	50.81	51.69
29	45.97	47.10	48.10	49.10	50.06	50.92	51.80
30	46.11	47.23	48.22	49.22	50.17	51.02	51.90
31	46.25	47.36	48.34	49.33	50.27	51.12	51.99
32	46.40	47.49	48.46	49.44	50.37	51.21	52.07
33	46.55	47.63	48.58	49.54	50.46	51.29	52.14
34	46.71	47.76	48.70	49.65	50.55	51.37	52.21
35	46.86	47.90	48.82	49.75	50.64	51.44	52.27
36	47.02	48.03	48.94	49.85	50.73	51.52	52.33

By Wuhan University Children’s Digital Health and Data Center (CDHDC)

The Postnatal Growth Reference for Preterm Infants

WUHAN UNIVERSITY

BMI(32w boys)

Corrected age(months)	Centiles						
	3rd	10rd	25rd	50rd	75rd	90rd	97rd
0	10.79	11.73	12.69	13.72	14.76	15.72	16.61
1	12.60	13.64	14.69	15.86	17.07	18.22	19.45
2	13.36	14.42	15.47	16.66	17.87	19.03	20.26
3	13.97	15.03	16.09	17.27	18.50	19.65	20.88
4	14.39	15.45	16.51	17.69	18.91	20.06	21.28
5	14.65	15.71	16.75	17.92	19.13	20.27	21.47
6	14.79	15.82	16.86	18.01	19.19	20.31	21.50
7	14.83	15.85	16.86	17.99	19.15	20.24	21.40
8	14.81	15.81	16.80	17.90	19.03	20.10	21.23
9	14.76	15.73	16.70	17.77	18.87	19.92	21.02
10	14.68	15.63	16.57	17.62	18.70	19.71	20.79
11	14.58	15.51	16.43	17.46	18.51	19.51	20.56
12	14.47	15.38	16.29	17.30	18.33	19.31	20.34
13	14.36	15.25	16.15	17.14	18.16	19.12	20.14
14	14.24	15.13	16.01	16.99	17.99	18.94	19.94
15	14.13	15.01	15.88	16.84	17.83	18.77	19.76
16	14.03	14.90	15.76	16.71	17.69	18.61	19.59
17	13.94	14.80	15.65	16.59	17.55	18.47	19.43
18	13.86	14.71	15.55	16.48	17.43	18.34	19.29
19	13.79	14.63	15.46	16.38	17.33	18.22	19.16
20	13.73	14.56	15.38	16.29	17.23	18.11	19.05
21	13.67	14.49	15.31	16.21	17.14	18.01	18.94
22	13.61	14.43	15.23	16.13	17.05	17.91	18.83
23	13.56	14.36	15.16	16.05	16.95	17.81	18.71
24	13.50	14.29	15.09	15.96	16.86	17.71	18.60
25	13.44	14.23	15.01	15.88	16.77	17.61	18.50
26	13.39	14.17	14.94	15.80	16.69	17.52	18.39
27	13.33	14.11	14.88	15.73	16.61	17.43	18.30
28	13.29	14.06	14.82	15.67	16.54	17.36	18.22
29	13.25	14.01	14.77	15.62	16.48	17.29	18.15
30	13.22	13.98	14.73	15.57	16.43	17.24	18.09
31	13.19	13.95	14.70	15.53	16.39	17.19	18.04
32	13.17	13.93	14.68	15.51	16.36	17.16	18.01
33	13.16	13.91	14.66	15.49	16.34	17.14	17.98
34	13.15	13.90	14.65	15.47	16.32	17.12	17.96
35	13.14	13.89	14.63	15.46	16.30	17.10	17.94
36	13.12	13.87	14.61	15.44	16.28	17.07	17.91

By Wuhan University Children's Digital Health and Data Center (CDHDC)

The Postnatal Growth Reference for Preterm Infants

Length(31w boys)

Corrected age(months)	Centiles						
	3rd	10rd	25rd	50rd	75rd	90rd	97rd
0	45.00	47.80	49.50	51.15	53.00	54.50	55.60
1	50.30	52.15	53.83	55.56	57.25	58.81	60.45
2	53.49	55.36	57.06	58.81	60.53	62.11	63.77
3	56.42	58.30	60.01	61.78	63.51	65.11	66.78
4	59.02	60.90	62.61	64.38	66.12	67.72	69.41
5	61.26	63.14	64.85	66.62	68.36	69.96	71.65
6	63.18	65.06	66.77	68.54	70.28	71.89	73.58
7	64.85	66.73	68.44	70.21	71.96	73.57	75.26
8	66.33	68.21	69.92	71.71	73.45	75.07	76.78
9	67.67	69.56	71.28	73.07	74.83	76.46	78.17
10	68.89	70.80	72.54	74.35	76.12	77.76	79.49
11	70.02	71.95	73.70	75.53	77.33	78.99	80.74
12	71.06	73.02	74.80	76.66	78.48	80.17	81.95
13	72.04	74.04	75.86	77.75	79.61	81.32	83.14
14	72.99	75.03	76.88	78.82	80.71	82.46	84.31
15	73.91	75.99	77.89	79.86	81.80	83.59	85.48
16	74.80	76.93	78.87	80.89	82.87	84.70	86.63
17	75.66	77.85	79.83	81.90	83.93	85.80	87.77
18	76.51	78.75	80.78	82.89	84.96	86.88	88.90
19	77.35	79.63	81.71	83.87	85.98	87.94	90.00
20	78.18	80.50	82.62	84.82	86.98	88.97	91.07
21	78.99	81.36	83.51	85.75	87.94	89.97	92.10
22	79.79	82.19	84.38	86.65	88.87	90.92	93.08
23	80.58	83.01	85.21	87.51	89.75	91.83	94.01
24	81.36	83.80	86.02	88.33	90.60	92.69	94.89
25	82.13	84.58	86.82	89.14	91.41	93.51	95.72
26	82.90	85.36	87.60	89.93	92.21	94.31	96.53
27	83.67	86.13	88.38	90.71	92.99	95.10	97.32
28	84.44	86.90	89.15	91.48	93.76	95.87	98.09
29	85.21	87.67	89.91	92.24	94.52	96.63	98.85
30	85.99	88.44	90.68	93.00	95.28	97.38	99.60
31	86.77	89.21	91.44	93.75	96.02	98.12	100.33
32	87.55	89.98	92.19	94.50	96.76	98.84	101.05
33	88.34	90.75	92.95	95.24	97.48	99.56	101.75
34	89.13	91.52	93.70	95.97	98.20	100.27	102.45
35	89.92	92.29	94.46	96.71	98.92	100.97	103.13
36	90.71	93.06	95.21	97.44	99.64	101.67	103.81

The Postnatal Growth Reference for Preterm Infants

WUHAN UNIVERSITY

Weight(31w boys)

Corrected age(months)	Centiles						
	3rd	10rd	25rd	50rd	75rd	90rd	97rd
0	2.38	2.75	3.15	3.60	4.00	4.40	4.80
1	3.41	3.86	4.31	4.82	5.35	5.86	6.42
2	4.17	4.69	5.21	5.79	6.39	6.97	7.61
3	4.86	5.43	6.00	6.63	7.29	7.92	8.62
4	5.45	6.06	6.66	7.33	8.02	8.69	9.43
5	5.94	6.58	7.20	7.89	8.61	9.31	10.07
6	6.35	7.00	7.64	8.35	9.08	9.80	10.57
7	6.68	7.35	8.00	8.72	9.47	10.19	10.98
8	6.97	7.65	8.31	9.04	9.79	10.52	11.32
9	7.23	7.91	8.58	9.32	10.08	10.82	11.62
10	7.45	8.14	8.82	9.56	10.33	11.08	11.88
11	7.65	8.34	9.03	9.78	10.56	11.31	12.13
12	7.82	8.53	9.22	9.98	10.77	11.53	12.36
13	7.97	8.69	9.39	10.17	10.97	11.74	12.58
14	8.11	8.84	9.56	10.35	11.16	11.95	12.80
15	8.25	8.99	9.72	10.53	11.36	12.16	13.03
16	8.37	9.14	9.88	10.71	11.56	12.38	13.27
17	8.50	9.28	10.04	10.89	11.76	12.60	13.51
18	8.62	9.42	10.20	11.07	11.96	12.82	13.76
19	8.75	9.56	10.36	11.25	12.17	13.05	14.00
20	8.87	9.71	10.53	11.43	12.37	13.27	14.25
21	9.00	9.85	10.68	11.61	12.56	13.48	14.48
22	9.12	9.99	10.84	11.78	12.75	13.69	14.71
23	9.24	10.12	10.99	11.94	12.93	13.89	14.92
24	9.36	10.26	11.13	12.10	13.10	14.07	15.12
25	9.48	10.39	11.27	12.25	13.27	14.25	15.31
26	9.60	10.52	11.41	12.41	13.43	14.42	15.50
27	9.73	10.65	11.55	12.56	13.60	14.60	15.68
28	9.85	10.78	11.70	12.71	13.76	14.77	15.86
29	9.98	10.92	11.85	12.87	13.92	14.94	16.05
30	10.12	11.07	12.00	13.03	14.09	15.12	16.23
31	10.26	11.21	12.15	13.19	14.26	15.30	16.42
32	10.40	11.37	12.31	13.36	14.44	15.48	16.61
33	10.55	11.52	12.47	13.52	14.61	15.66	16.79
34	10.70	11.67	12.63	13.69	14.78	15.83	16.97
35	10.84	11.82	12.79	13.85	14.95	16.01	17.15
36	10.99	11.98	12.94	14.01	15.12	16.18	17.33

By Wuhan University Children’s Digital Health and Data Center (CDHDC)

The Postnatal Growth Reference for Preterm Infants

Head circumference(31w boys)

Corrected age(months)	Centiles						
	3rd	10rd	25rd	50rd	75rd	90rd	97rd
0	32.00	33.50	34.20	35.20	36.00	37.00	37.71
1	34.69	35.67	36.54	37.42	38.28	39.07	39.93
2	36.14	37.13	38.00	38.89	39.76	40.57	41.43
3	37.47	38.46	39.34	40.24	41.11	41.92	42.80
4	38.63	39.62	40.51	41.41	42.29	43.11	43.99
5	39.61	40.61	41.50	42.41	43.29	44.12	45.00
6	40.43	41.43	42.32	43.24	44.13	44.96	45.84
7	41.10	42.11	43.01	43.92	44.82	45.65	46.55
8	41.66	42.68	43.58	44.50	45.40	46.23	47.13
9	42.14	43.15	44.06	44.98	45.89	46.73	47.63
10	42.53	43.56	44.46	45.39	46.30	47.15	48.05
11	42.87	43.90	44.81	45.75	46.66	47.51	48.42
12	43.16	44.19	45.11	46.05	46.97	47.82	48.74
13	43.40	44.44	45.37	46.31	47.24	48.10	49.02
14	43.62	44.67	45.59	46.55	47.48	48.34	49.27
15	43.81	44.86	45.80	46.76	47.70	48.57	49.50
16	43.98	45.05	45.99	46.96	47.90	48.78	49.72
17	44.15	45.22	46.17	47.14	48.09	48.98	49.93
18	44.30	45.38	46.34	47.32	48.28	49.17	50.13
19	44.44	45.53	46.50	47.49	48.45	49.35	50.32
20	44.58	45.67	46.65	47.65	48.62	49.53	50.50
21	44.71	45.81	46.79	47.80	48.78	49.69	50.67
22	44.83	45.94	46.93	47.94	48.93	49.85	50.83
23	44.95	46.07	47.06	48.08	49.07	49.99	50.98
24	45.07	46.19	47.19	48.21	49.20	50.13	51.12
25	45.20	46.32	47.32	48.33	49.33	50.26	51.25
26	45.32	46.44	47.44	48.46	49.45	50.37	51.37
27	45.45	46.57	47.56	48.57	49.56	50.49	51.47
28	45.58	46.69	47.68	48.69	49.67	50.59	51.57
29	45.71	46.81	47.79	48.79	49.77	50.68	51.66
30	45.84	46.93	47.90	48.89	49.86	50.77	51.74
31	45.97	47.05	48.01	48.99	49.95	50.85	51.81
32	46.10	47.17	48.11	49.08	50.03	50.92	51.87
33	46.24	47.29	48.22	49.18	50.11	50.99	51.92
34	46.38	47.41	48.33	49.27	50.19	51.05	51.98
35	46.53	47.54	48.44	49.37	50.27	51.12	52.02
36	46.67	47.67	48.55	49.46	50.35	51.18	52.07

The Postnatal Growth Reference for Preterm Infants

WUHAN UNIVERSITY

BMI(31w boys)

Corrected age(months)	Centiles						
	3rd	10rd	25rd	50rd	75rd	90rd	97rd
0	10.58	11.72	12.51	13.62	14.80	15.52	16.80
1	12.41	13.44	14.48	15.65	16.89	18.10	19.45
2	13.15	14.18	15.21	16.38	17.60	18.81	20.13
3	13.75	14.78	15.81	16.96	18.17	19.36	20.66
4	14.20	15.22	16.23	17.37	18.57	19.73	21.01
5	14.50	15.50	16.50	17.62	18.79	19.93	21.18
6	14.67	15.65	16.63	17.72	18.87	19.98	21.20
7	14.75	15.71	16.66	17.72	18.83	19.91	21.10
8	14.76	15.70	16.62	17.65	18.73	19.77	20.92
9	14.73	15.64	16.53	17.53	18.57	19.58	20.69
10	14.67	15.55	16.41	17.38	18.39	19.37	20.43
11	14.58	15.43	16.27	17.21	18.19	19.14	20.17
12	14.46	15.30	16.12	17.03	17.99	18.91	19.92
13	14.33	15.15	15.95	16.85	17.78	18.69	19.67
14	14.19	14.99	15.79	16.67	17.59	18.48	19.45
15	14.04	14.84	15.63	16.50	17.41	18.29	19.25
16	13.90	14.70	15.48	16.35	17.25	18.13	19.08
17	13.77	14.56	15.34	16.21	17.11	17.98	18.93
18	13.65	14.43	15.21	16.08	16.98	17.85	18.80
19	13.53	14.32	15.10	15.96	16.86	17.73	18.68
20	13.43	14.22	14.99	15.86	16.76	17.63	18.58
21	13.34	14.12	14.90	15.76	16.66	17.53	18.49
22	13.25	14.03	14.81	15.67	16.57	17.44	18.39
23	13.16	13.95	14.72	15.58	16.48	17.35	18.31
24	13.08	13.86	14.64	15.50	16.39	17.26	18.21
25	13.00	13.78	14.55	15.41	16.31	17.17	18.12
26	12.93	13.71	14.48	15.33	16.22	17.08	18.03
27	12.87	13.64	14.40	15.25	16.14	17.00	17.93
28	12.82	13.58	14.34	15.18	16.06	16.91	17.84
29	12.77	13.53	14.28	15.12	15.99	16.83	17.76
30	12.73	13.49	14.23	15.06	15.93	16.77	17.68
31	12.70	13.45	14.19	15.02	15.87	16.70	17.61
32	12.68	13.42	14.15	14.97	15.82	16.65	17.55
33	12.65	13.39	14.12	14.93	15.78	16.60	17.49
34	12.63	13.36	14.09	14.89	15.73	16.55	17.43
35	12.60	13.33	14.05	14.85	15.69	16.49	17.37
36	12.57	13.29	14.01	14.81	15.63	16.44	17.31

By Wuhan University Children's Digital Health and Data Center (CDHDC)

The Postnatal Growth Reference for Preterm Infants

Length (30w boys)

Corrected age(months)	Centiles						
	3rd	10rd	25rd	50rd	75rd	90rd	97rd
0	45.00	47.27	49.00	51.00	52.50	53.50	54.73
1	50.27	51.92	53.51	55.21	56.84	58.25	59.59
2	53.46	55.13	56.75	58.47	60.13	61.57	62.94
3	56.38	58.07	59.70	61.45	63.13	64.59	65.98
4	58.95	60.64	62.28	64.04	65.73	67.20	68.61
5	61.13	62.82	64.47	66.24	67.94	69.42	70.84
6	62.99	64.68	66.33	68.10	69.81	71.30	72.73
7	64.57	66.27	67.93	69.71	71.43	72.93	74.37
8	65.95	67.67	69.34	71.15	72.88	74.40	75.85
9	67.19	68.93	70.63	72.46	74.22	75.76	77.23
10	68.33	70.10	71.83	73.69	75.48	77.04	78.54
11	69.39	71.19	72.95	74.84	76.66	78.25	79.78
12	70.39	72.22	74.01	75.93	77.79	79.41	80.96
13	71.36	73.23	75.05	77.00	78.89	80.53	82.11
14	72.33	74.22	76.07	78.06	79.97	81.64	83.24
15	73.29	75.21	77.09	79.11	81.05	82.75	84.37
16	74.24	76.19	78.10	80.15	82.12	83.84	85.49
17	75.19	77.17	79.10	81.18	83.18	84.92	86.59
18	76.11	78.12	80.08	82.18	84.21	85.98	87.67
19	77.01	79.04	81.03	83.16	85.21	87.00	88.72
20	77.87	79.93	81.95	84.11	86.19	88.00	89.74
21	78.70	80.79	82.84	85.03	87.14	88.98	90.74
22	79.50	81.63	83.70	85.92	88.06	89.93	91.72
23	80.27	82.43	84.54	86.79	88.97	90.87	92.68
24	81.01	83.21	85.35	87.64	89.86	91.78	93.63
25	81.72	83.96	86.14	88.47	90.72	92.68	94.56
26	82.41	84.69	86.90	89.28	91.57	93.56	95.47
27	83.08	85.39	87.65	90.06	92.39	94.41	96.35
28	83.72	86.08	88.37	90.83	93.19	95.25	97.22
29	84.36	86.75	89.08	91.58	93.98	96.07	98.07
30	84.98	87.41	89.78	92.32	94.75	96.87	98.90
31	85.59	88.06	90.47	93.04	95.52	97.67	99.73
32	86.18	88.70	91.15	93.76	96.28	98.47	100.56
33	86.78	89.34	91.83	94.48	97.04	99.26	101.38
34	87.38	89.98	92.51	95.20	97.79	100.04	102.20
35	87.99	90.63	93.19	95.92	98.55	100.83	103.01
36	88.60	91.27	93.87	96.64	99.31	101.62	103.73

By Wuhan University Children’s Digital Health and Data Center (CDHDC)

The Postnatal Growth Reference for Preterm Infants

WUHAN UNIVERSITY

Weight(30w boys)

Corrected age(months)	Centiles						
	3rd	10rd	25rd	50rd	75rd	90rd	97rd
0	2.27	2.72	3.15	3.50	4.00	4.33	4.70
1	3.07	3.58	4.09	4.65	5.19	5.67	6.14
2	3.87	4.43	4.98	5.59	6.18	6.71	7.22
3	4.60	5.19	5.78	6.42	7.05	7.60	8.15
4	5.25	5.86	6.46	7.12	7.77	8.35	8.91
5	5.78	6.40	7.02	7.69	8.36	8.95	9.53
6	6.21	6.84	7.47	8.16	8.84	9.44	10.03
7	6.57	7.21	7.85	8.55	9.24	9.85	10.45
8	6.86	7.51	8.17	8.88	9.58	10.21	10.82
9	7.11	7.78	8.45	9.18	9.91	10.55	11.18
10	7.32	8.02	8.71	9.46	10.21	10.87	11.52
11	7.52	8.23	8.94	9.72	10.49	11.17	11.84
12	7.69	8.43	9.16	9.96	10.75	11.45	12.14
13	7.86	8.61	9.36	10.18	10.99	11.72	12.42
14	8.01	8.78	9.55	10.39	11.22	11.96	12.68
15	8.17	8.95	9.73	10.59	11.43	12.18	12.92
16	8.32	9.11	9.91	10.78	11.63	12.39	13.14
17	8.47	9.28	10.08	10.96	11.82	12.59	13.34
18	8.63	9.44	10.25	11.13	12.00	12.77	13.53
19	8.79	9.60	10.41	11.29	12.17	12.94	13.70
20	8.95	9.76	10.57	11.45	12.33	13.11	13.87
21	9.10	9.91	10.72	11.61	12.48	13.26	14.02
22	9.26	10.07	10.87	11.76	12.63	13.41	14.17
23	9.41	10.21	11.02	11.91	12.78	13.56	14.32
24	9.55	10.36	11.17	12.06	12.93	13.71	14.47
25	9.70	10.51	11.32	12.21	13.09	13.87	14.63
26	9.84	10.65	11.47	12.36	13.24	14.02	14.79
27	9.98	10.80	11.61	12.51	13.40	14.18	14.95
28	10.12	10.94	11.76	12.66	13.55	14.34	15.12
29	10.27	11.09	11.91	12.82	13.71	14.51	15.29
30	10.41	11.24	12.07	12.98	13.88	14.68	15.46
31	10.57	11.40	12.23	13.14	14.05	14.85	15.63
32	10.73	11.56	12.40	13.31	14.22	15.03	15.82
33	10.89	11.73	12.57	13.49	14.40	15.21	16.00
34	11.05	11.90	12.74	13.66	14.58	15.39	16.18
35	11.22	12.07	12.91	13.84	14.75	15.57	16.37
36	11.39	12.23	13.08	14.01	14.93	15.75	16.56

By Wuhan University Children’s Digital Health and Data Center (CDHDC)

The Postnatal Growth Reference for Preterm Infants

WUHAN UNIVERSITY

Head circumference(30w boys)

Corrected age(months)	Centiles						
	3rd	10rd	25rd	50rd	75rd	90rd	97rd
0	32.00	33.00	34.00	35.00	36.00	36.80	37.71
1	34.32	35.33	36.29	37.31	38.26	39.08	39.86
2	35.79	36.79	37.74	38.74	39.68	40.50	41.27
3	37.16	38.13	39.07	40.06	41.00	41.81	42.58
4	38.37	39.33	40.25	41.23	42.16	42.96	43.73
5	39.41	40.36	41.27	42.24	43.17	43.96	44.72
6	40.29	41.23	42.14	43.10	44.02	44.82	45.57
7	41.02	41.96	42.86	43.82	44.74	45.54	46.29
8	41.63	42.56	43.47	44.43	45.35	46.14	46.90
9	42.13	43.07	43.98	44.94	45.87	46.67	47.43
10	42.54	43.49	44.40	45.37	46.30	47.11	47.88
11	42.88	43.83	44.76	45.74	46.67	47.49	48.26
12	43.16	44.13	45.06	46.05	46.99	47.81	48.60
13	43.40	44.38	45.32	46.32	47.27	48.10	48.89
14	43.61	44.60	45.54	46.55	47.52	48.35	49.15
15	43.81	44.79	45.75	46.77	47.74	48.58	49.38
16	43.99	44.98	45.94	46.96	47.94	48.78	49.58
17	44.17	45.17	46.13	47.15	48.12	48.96	49.77
18	44.35	45.34	46.30	47.32	48.29	49.13	49.94
19	44.52	45.51	46.46	47.48	48.45	49.29	50.09
20	44.68	45.67	46.62	47.63	48.59	49.43	50.23
21	44.85	45.82	46.77	47.77	48.73	49.56	50.36
22	45.01	45.98	46.91	47.91	48.87	49.69	50.49
23	45.16	46.12	47.06	48.05	49.00	49.82	50.61
24	45.32	46.27	47.20	48.18	49.13	49.95	50.73
25	45.47	46.42	47.34	48.32	49.26	50.07	50.85
26	45.61	46.55	47.47	48.45	49.38	50.19	50.97
27	45.75	46.69	47.60	48.57	49.50	50.31	51.08
28	45.88	46.81	47.72	48.69	49.62	50.42	51.19
29	46.01	46.94	47.85	48.81	49.73	50.54	51.31
30	46.15	47.07	47.97	48.93	49.85	50.65	51.42
31	46.29	47.21	48.11	49.06	49.98	50.77	51.54
32	46.45	47.36	48.25	49.20	50.11	50.90	51.66
33	46.61	47.51	48.39	49.33	50.24	51.02	51.77
34	46.77	47.67	48.54	49.47	50.36	51.14	51.89
35	46.94	47.82	48.69	49.61	50.49	51.26	52.00
36	47.10	47.98	48.83	49.74	50.62	51.38	52.11

By Wuhan University Children’s Digital Health and Data Center (CDHDC)

The Postnatal Growth Reference for Preterm Infants

WUHAN UNIVERSITY

BMI(30w boys)

Corrected age(months)	Centiles						
	3rd	10rd	25rd	50rd	75rd	90rd	97rd
0	10.54	11.45	12.49	13.69	14.72	15.74	16.91
1	12.16	13.27	14.40	15.25	17.00	18.19	19.39
2	12.91	13.99	15.11	15.99	17.65	18.81	19.98
3	13.53	14.59	15.68	16.76	18.16	19.30	20.44
4	14.00	15.03	16.10	17.15	18.52	19.63	20.74
5	14.32	15.33	16.36	17.41	18.71	19.79	20.87
6	14.52	15.50	16.51	17.54	18.79	19.83	20.87
7	14.62	15.58	16.55	17.59	18.77	19.78	20.79
8	14.66	15.58	16.53	17.56	18.68	19.67	20.65
9	14.63	15.54	16.46	17.47	18.56	19.52	20.48
10	14.57	15.45	16.36	17.36	18.41	19.35	20.28
11	14.48	15.34	16.23	17.22	18.25	19.17	20.08
12	14.36	15.21	16.09	17.08	18.08	18.99	19.89
13	14.22	15.07	15.94	16.93	17.91	18.81	19.71
14	14.08	14.92	15.79	16.78	17.76	18.66	19.55
15	13.93	14.78	15.65	16.64	17.62	18.52	19.42
16	13.79	14.64	15.52	16.52	17.49	18.40	19.30
17	13.67	14.52	15.40	16.41	17.39	18.30	19.20
18	13.56	14.42	15.30	16.31	17.30	18.21	19.13
19	13.48	14.34	15.22	16.22	17.22	18.14	19.05
20	13.41	14.27	15.15	16.14	17.15	18.06	18.98
21	13.35	14.21	15.08	16.07	17.07	17.98	18.89
22	13.30	14.15	15.02	15.99	16.98	17.88	18.78
23	13.25	14.09	14.95	15.91	16.89	17.78	18.67
24	13.21	14.03	14.88	15.83	16.80	17.67	18.55
25	13.17	13.98	14.82	15.76	16.71	17.57	18.43
26	13.13	13.93	14.75	15.68	16.62	17.47	18.32
27	13.09	13.88	14.70	15.62	16.54	17.38	18.22
28	13.06	13.85	14.65	15.56	16.47	17.30	18.13
29	13.04	13.82	14.61	15.51	16.41	17.24	18.06
30	13.04	13.81	14.60	15.47	16.38	17.20	18.01
31	13.06	13.82	14.60	15.45	16.37	17.18	17.99
32	13.10	13.86	14.64	15.43	16.40	17.20	18.00
33	13.16	13.91	14.69	15.42	16.44	17.24	18.04
34	13.22	13.98	14.75	15.41	16.50	17.30	18.09
35	13.30	14.05	14.82	15.40	16.56	17.36	18.15
36	13.37	14.12	14.89	15.38	16.63	17.43	18.22

By Wuhan University Children’s Digital Health and Data Center (CDHDC)

The Postnatal Growth Reference for Preterm Infants

WUHAN UNIVERSITY

Length (29w boys)

Corrected age(months)	Centiles						
	3rd	10rd	25rd	50rd	75rd	90rd	97rd
0	46.00	47.30	48.80	50.60	52.50	54.00	56.48
1	49.16	51.00	53.04	55.01	56.96	58.51	60.01
2	53.26	54.92	56.57	58.38	60.15	61.71	63.23
3	56.18	57.85	59.51	61.32	63.09	64.67	66.20
4	58.83	60.49	62.14	63.95	65.72	67.30	68.83
5	61.18	62.83	64.46	66.26	68.02	69.58	71.11
6	63.24	64.86	66.49	68.27	70.02	71.57	73.09
7	65.03	66.65	68.26	70.03	71.77	73.31	74.82
8	66.59	68.20	69.81	71.57	73.30	74.84	76.34
9	67.96	69.57	71.17	72.93	74.66	76.20	77.70
10	69.16	70.77	72.38	74.14	75.88	77.43	78.93
11	70.22	71.84	73.46	75.24	76.99	78.54	80.06
12	71.17	72.81	74.44	76.24	78.00	79.57	81.10
13	72.05	73.71	75.36	77.18	78.97	80.55	82.10
14	72.88	74.56	76.24	78.08	79.90	81.52	83.09
15	73.69	75.40	77.11	78.99	80.83	82.48	84.08
16	74.51	76.25	77.99	79.90	81.78	83.45	85.07
17	75.34	77.11	78.87	80.81	82.72	84.42	86.07
18	76.19	77.98	79.77	81.74	83.68	85.40	87.08
19	77.05	78.87	80.69	82.68	84.64	86.38	88.08
20	77.93	79.77	81.60	83.62	85.60	87.36	89.08
21	78.79	80.65	82.51	84.54	86.55	88.33	90.06
22	79.64	81.51	83.39	85.44	87.47	89.27	91.02
23	80.46	82.35	84.24	86.31	88.36	90.17	91.94
24	81.25	83.16	85.06	87.15	89.21	91.04	92.83
25	82.01	83.93	85.86	87.96	90.04	91.88	93.68
26	82.75	84.69	86.62	88.74	90.84	92.69	94.50
27	83.47	85.42	87.37	89.50	91.60	93.47	95.30
28	84.17	86.13	88.09	90.24	92.35	94.23	96.06
29	84.86	86.83	88.80	90.95	93.08	94.96	96.81
30	85.54	87.51	89.48	91.65	93.78	95.67	97.52
31	86.20	88.18	90.16	92.32	94.46	96.36	98.22
32	86.85	88.83	90.82	92.99	95.13	97.04	98.89
33	87.50	89.48	91.47	93.64	95.79	97.70	99.56
34	88.14	90.12	92.11	94.29	96.44	98.35	100.21
35	88.77	90.76	92.76	94.94	97.09	99.00	100.87
36	89.41	91.41	93.40	95.58	97.74	99.65	101.52

By Wuhan University Children’s Digital Health and Data Center (CDHDC)

The Postnatal Growth Reference for Preterm Infants

WUHAN UNIVERSITY

Weight(29w boys)

Corrected age(months)	Centiles						
	3rd	10rd	25rd	50rd	75rd	90rd	97rd
0	2.50	2.76	3.00	3.40	3.90	4.32	4.93
1	3.38	3.80	4.25	4.75	5.28	5.76	6.25
2	4.11	4.59	5.10	5.69	6.28	6.84	7.39
3	4.77	5.31	5.87	6.51	7.17	7.78	8.39
4	5.35	5.93	6.52	7.21	7.91	8.55	9.20
5	5.83	6.43	7.06	7.77	8.50	9.17	9.84
6	6.23	6.85	7.49	8.23	8.98	9.67	10.36
7	6.56	7.20	7.85	8.60	9.37	10.08	10.79
8	6.84	7.49	8.16	8.92	9.71	10.42	11.14
9	7.08	7.74	8.42	9.20	9.99	10.72	11.45
10	7.29	7.96	8.64	9.43	10.23	10.97	11.70
11	7.46	8.14	8.83	9.62	10.43	11.18	11.92
12	7.61	8.29	8.99	9.79	10.61	11.36	12.11
13	7.74	8.43	9.14	9.94	10.77	11.53	12.28
14	7.87	8.56	9.28	10.09	10.92	11.68	12.45
15	8.00	8.70	9.42	10.24	11.08	11.84	12.62
16	8.14	8.84	9.57	10.39	11.24	12.01	12.78
17	8.29	9.00	9.73	10.55	11.40	12.17	12.95
18	8.46	9.16	9.89	10.72	11.57	12.35	13.13
19	8.63	9.34	10.07	10.91	11.76	12.53	13.32
20	8.81	9.52	10.26	11.09	11.95	12.73	13.51
21	8.99	9.70	10.45	11.29	12.14	12.93	13.72
22	9.16	9.88	10.63	11.47	12.34	13.13	13.92
23	9.31	10.04	10.80	11.66	12.53	13.33	14.13
24	9.46	10.20	10.96	11.83	12.72	13.53	14.34
25	9.59	10.34	11.12	12.00	12.90	13.72	14.55
26	9.71	10.48	11.27	12.17	13.08	13.92	14.76
27	9.83	10.61	11.42	12.33	13.26	14.12	14.94
28	9.95	10.75	11.57	12.50	13.45	14.31	15.11
29	10.07	10.88	11.72	12.66	13.63	14.48	15.28
30	10.20	11.02	11.87	12.83	13.81	14.66	15.41
31	10.32	11.16	12.02	12.99	13.99	14.84	15.62
32	10.45	11.29	12.17	13.16	14.17	15.02	15.80
33	10.58	11.43	12.32	13.33	14.35	15.19	16.00
34	10.71	11.57	12.47	13.49	14.53	15.38	16.13
35	10.83	11.71	12.62	13.65	14.70	15.56	16.33
36	10.96	11.85	12.77	13.81	14.87	15.74	16.52

The Postnatal Growth Reference for Preterm Infants

WUHAN UNIVERSITY

Head circumference(29w boys)

Corrected age(months)	Centiles						
	3rd	10rd	25rd	50rd	75rd	90rd	97rd
0	32.00	33.00	34.00	34.80	35.80	36.66	37.47
1	34.46	35.35	36.23	37.17	38.10	38.91	39.69
2	35.84	36.74	37.62	38.58	39.52	40.34	41.14
3	37.13	38.03	38.92	39.89	40.83	41.66	42.47
4	38.28	39.19	40.08	41.05	42.00	42.83	43.64
5	39.29	40.20	41.09	42.06	43.01	43.85	44.65
6	40.18	41.07	41.96	42.93	43.87	44.70	45.51
7	40.94	41.82	42.71	43.66	44.60	45.42	46.22
8	41.58	42.46	43.33	44.28	45.21	46.02	46.82
9	42.13	43.00	43.86	44.79	45.71	46.52	47.30
10	42.59	43.45	44.29	45.22	46.12	46.92	47.70
11	42.97	43.81	44.65	45.56	46.46	47.25	48.02
12	43.28	44.11	44.95	45.85	46.74	47.52	48.29
13	43.54	44.37	45.20	46.10	46.98	47.76	48.52
14	43.76	44.59	45.42	46.32	47.20	47.97	48.73
15	43.97	44.79	45.62	46.52	47.40	48.18	48.93
16	44.15	44.98	45.80	46.70	47.59	48.37	49.12
17	44.31	45.15	45.97	46.88	47.76	48.54	49.30
18	44.47	45.30	46.13	47.04	47.92	48.71	49.47
19	44.62	45.46	46.29	47.19	48.08	48.87	49.63
20	44.78	45.62	46.45	47.35	48.24	49.03	49.79
21	44.94	45.78	46.61	47.52	48.40	49.19	49.95
22	45.10	45.94	46.77	47.68	48.57	49.36	50.12
23	45.25	46.09	46.93	47.84	48.73	49.52	50.28
24	45.41	46.25	47.08	47.99	48.89	49.68	50.44
25	45.56	46.40	47.23	48.14	49.04	49.83	50.60
26	45.70	46.54	47.38	48.29	49.18	49.97	50.74
27	45.85	46.69	47.52	48.43	49.32	50.10	50.87
28	45.99	46.83	47.66	48.56	49.45	50.23	50.99
29	46.13	46.96	47.79	48.68	49.57	50.35	51.11
30	46.27	47.09	47.91	48.80	49.68	50.46	51.21
31	46.41	47.22	48.04	48.92	49.79	50.56	51.31
32	46.55	47.35	48.16	49.03	49.89	50.65	51.39
33	46.68	47.48	48.27	49.13	49.98	50.74	51.47
34	46.81	47.60	48.38	49.23	50.07	50.81	51.53
35	46.94	47.71	48.48	49.32	50.14	50.87	51.58
36	47.06	47.82	48.58	49.40	50.22	50.94	51.64

By Wuhan University Children's Digital Health and Data Center (CDHDC)

The Postnatal Growth Reference for Preterm Infants

WUHAN UNIVERSITY

BMI(29w boys)

Corrected age(months)	Centiles						
	3rd	10rd	25rd	50rd	75rd	90rd	97rd
0	11.08	11.51	12.32	13.47	14.15	15.20	16.63
1	12.42	13.38	14.41	15.62	16.90	18.12	19.37
2	13.06	14.04	15.09	16.32	17.62	18.85	20.12
3	13.59	14.58	15.64	16.88	18.19	19.43	20.70
4	13.97	14.97	16.03	17.27	18.58	19.81	21.09
5	14.21	15.20	16.26	17.49	18.79	20.01	21.28
6	14.32	15.31	16.35	17.57	18.86	20.07	21.31
7	14.35	15.32	16.36	17.56	18.82	20.02	21.25
8	14.33	15.29	16.30	17.49	18.73	19.90	21.11
9	14.27	15.22	16.21	17.37	18.59	19.74	20.92
10	14.20	15.12	16.10	17.23	18.42	19.54	20.70
11	14.11	15.01	15.96	17.07	18.23	19.32	20.44
12	14.00	14.88	15.81	16.89	18.02	19.09	20.18
13	13.90	14.75	15.66	16.71	17.82	18.85	19.91
14	13.80	14.63	15.52	16.54	17.62	18.62	19.65
15	13.71	14.53	15.39	16.39	17.43	18.41	19.41
16	13.64	14.43	15.27	16.25	17.26	18.21	19.19
17	13.57	14.35	15.17	16.12	17.11	18.03	18.98
18	13.52	14.28	15.08	16	16.97	17.87	18.80
19	13.48	14.22	15.01	15.91	16.85	17.74	18.64
20	13.45	14.18	14.95	15.83	16.76	17.62	18.50
21	13.42	14.14	14.89	15.77	16.67	17.52	18.39
22	13.38	14.09	14.84	15.7	16.59	17.43	18.28
23	13.34	14.04	14.78	15.63	16.51	17.34	18.19
24	13.27	13.97	14.71	15.56	16.44	17.26	18.10
25	13.20	13.90	14.63	15.48	16.36	17.18	18.02
26	13.13	13.83	14.56	15.41	16.29	17.12	17.96
27	13.06	13.76	14.50	15.35	16.24	17.07	17.91
28	12.99	13.70	14.44	15.3	16.19	17.02	17.88
29	12.93	13.64	14.39	15.25	16.15	17.00	17.86
30	12.87	13.59	14.34	15.22	16.13	16.99	17.86
31	12.81	13.54	14.31	15.2	16.13	16.99	17.88
32	12.75	13.49	14.28	15.18	16.13	17.01	17.92
33	12.69	13.45	14.24	15.17	16.13	17.04	17.97
34	12.63	13.40	14.21	15.15	16.14	17.07	18.02
35	12.56	13.35	14.17	15.14	16.15	17.09	18.07
36	12.49	13.29	14.14	15.12	16.15	17.12	18.11

By Wuhan University Children’s Digital Health and Data Center (CDHDC)

The Postnatal Growth Reference for Preterm Infants

WUHAN UNIVERSITY

Length (28w boys)

Corrected age(months)	Centiles						
	3rd	10rd	25rd	50rd	75rd	90rd	97rd
0	44.40	46.25	48.03	50.00	51.93	53.70	55.60
1	49.14	51.05	52.94	54.99	57.02	58.82	60.60
2	52.17	54.08	55.97	58.03	60.07	61.88	63.67
3	55.00	56.92	58.81	60.88	62.92	64.74	66.53
4	57.57	59.49	61.38	63.45	65.49	67.32	69.11
5	59.85	61.76	63.65	65.72	67.76	69.59	71.38
6	61.84	63.74	65.64	67.70	69.74	71.57	73.36
7	63.58	65.48	67.37	69.44	71.48	73.30	75.10
8	65.12	67.02	68.91	70.97	73.01	74.83	76.63
9	66.49	68.39	70.28	72.34	74.38	76.20	78.00
10	67.74	69.64	71.52	73.58	75.62	77.44	79.24
11	68.88	70.78	72.66	74.72	76.75	78.58	80.37
12	69.95	71.84	73.72	75.78	77.81	79.63	81.43
13	70.95	72.84	74.72	76.78	78.81	80.63	82.43
14	71.89	73.79	75.67	77.73	79.77	81.60	83.40
15	72.80	74.71	76.60	78.67	80.71	82.55	84.36
16	73.71	75.62	77.52	79.60	81.66	83.50	85.32
17	74.62	76.54	78.45	80.54	82.61	84.46	86.29
18	75.54	77.47	79.39	81.49	83.56	85.42	87.26
19	76.46	78.40	80.32	82.43	84.51	86.37	88.21
20	77.38	79.31	81.24	83.35	85.44	87.30	89.15
21	78.27	80.21	82.14	84.25	86.35	88.22	90.07
22	79.13	81.08	83.01	85.13	87.23	89.11	90.96
23	79.95	81.91	83.85	85.98	88.09	89.97	91.83
24	80.74	82.71	84.66	86.80	88.91	90.81	92.68
25	81.49	83.47	85.43	87.59	89.72	91.63	93.51
26	82.20	84.20	86.18	88.35	90.50	92.42	94.32
27	82.89	84.90	86.90	89.09	91.26	93.20	95.12
28	83.54	85.58	87.60	89.82	92.01	93.97	95.91
29	84.17	86.23	88.29	90.53	92.75	94.74	96.71
30	84.77	86.87	88.95	91.23	93.48	95.51	97.50
31	85.34	87.47	89.59	91.91	94.21	96.26	98.29
32	85.89	88.06	90.22	92.58	94.92	97.01	99.08
33	86.41	88.63	90.83	93.24	95.62	97.76	99.87
34	86.92	89.18	91.43	93.88	96.32	98.49	100.64
35	87.41	89.72	92.01	94.52	97.00	99.22	101.42
36	87.90	90.26	92.60	95.16	97.69	99.96	102.19

By Wuhan University Children's Digital Health and Data Center (CDHDC)

The Postnatal Growth Reference for Preterm Infants

WUHAN UNIVERSITY

Weight(28w boys)

Corrected age(months)	Centiles						
	3rd	10rd	25rd	50rd	75rd	90rd	97rd
0	2.31	2.67	3.00	3.40	3.90	4.52	5.10
1	2.97	3.45	3.95	4.53	5.12	5.67	6.22
2	3.64	4.18	4.74	5.37	6.03	6.63	7.24
3	4.28	4.85	5.45	6.14	6.84	7.49	8.14
4	4.85	5.45	6.08	6.80	7.53	8.20	8.88
5	5.34	5.97	6.61	7.35	8.10	8.79	9.48
6	5.76	6.39	7.05	7.80	8.56	9.26	9.97
7	6.10	6.75	7.42	8.17	8.95	9.66	10.37
8	6.39	7.05	7.72	8.49	9.27	9.99	10.71
9	6.64	7.30	7.99	8.76	9.55	10.28	11.01
10	6.85	7.52	8.22	9.00	9.80	10.53	11.27
11	7.05	7.72	8.42	9.21	10.02	10.75	11.49
12	7.23	7.90	8.61	9.40	10.21	10.95	11.69
13	7.39	8.08	8.78	9.58	10.39	11.13	11.88
14	7.55	8.24	8.94	9.74	10.56	11.30	12.05
15	7.70	8.39	9.10	9.90	10.72	11.46	12.21
16	7.85	8.54	9.25	10.06	10.88	11.63	12.37
17	8.01	8.70	9.41	10.22	11.04	11.78	12.53
18	8.17	8.86	9.57	10.38	11.20	11.94	12.69
19	8.34	9.03	9.74	10.54	11.36	12.11	12.86
20	8.50	9.19	9.90	10.71	11.52	12.27	13.02
21	8.67	9.36	10.07	10.88	11.69	12.44	13.19
22	8.83	9.52	10.24	11.04	11.87	12.62	13.37
23	8.97	9.67	10.40	11.21	12.04	12.80	13.55
24	9.11	9.82	10.55	11.38	12.21	12.98	13.75
25	9.24	9.96	10.70	11.54	12.39	13.17	13.94
26	9.35	10.09	10.84	11.69	12.56	13.35	14.14
27	9.47	10.21	10.98	11.84	12.73	13.53	14.34
28	9.57	10.33	11.11	12.00	12.89	13.72	14.54
29	9.68	10.45	11.25	12.15	13.06	13.90	14.74
30	9.79	10.57	11.38	12.30	13.23	14.09	14.94
31	9.89	10.69	11.52	12.45	13.40	14.27	15.14
32	10.00	10.82	11.66	12.61	13.57	14.45	15.34
33	10.11	10.94	11.79	12.76	13.74	14.64	15.53
34	10.22	11.06	11.93	12.91	13.90	14.81	15.72
35	10.33	11.18	12.06	13.05	14.06	14.99	15.91
36	10.44	11.30	12.19	13.20	14.22	15.16	16.10

By Wuhan University Children’s Digital Health and Data Center (CDHDC)

The Postnatal Growth Reference for Preterm Infants

WUHAN UNIVERSITY

Head circumference(28w boys)

Corrected age(months)	Centiles						
	3rd	10rd	25rd	50rd	75rd	90rd	97rd
0	31.00	32.00	33.50	34.50	35.40	36.62	38.26
1	34.11	35.06	35.99	37.00	37.97	38.81	39.63
2	35.57	36.49	37.40	38.38	39.33	40.16	40.96
3	36.91	37.82	38.71	39.67	40.61	41.43	42.21
4	38.11	39.00	39.88	40.83	41.75	42.56	43.35
5	39.15	40.03	40.90	41.84	42.76	43.56	44.34
6	40.03	40.91	41.77	42.71	43.62	44.41	45.19
7	40.78	41.65	42.51	43.44	44.34	45.14	45.90
8	41.42	42.28	43.13	44.06	44.96	45.75	46.51
9	41.97	42.82	43.67	44.59	45.48	46.27	47.03
10	42.43	43.28	44.12	45.04	45.93	46.71	47.47
11	42.83	43.67	44.51	45.42	46.30	47.08	47.84
12	43.16	44.00	44.84	45.74	46.62	47.40	48.15
13	43.45	44.29	45.12	46.02	46.89	47.67	48.42
14	43.69	44.53	45.36	46.26	47.13	47.91	48.66
15	43.89	44.73	45.56	46.46	47.34	48.12	48.87
16	44.05	44.90	45.73	46.64	47.53	48.31	49.06
17	44.19	45.04	45.89	46.80	47.69	48.48	49.24
18	44.32	45.17	46.02	46.94	47.84	48.63	49.39
19	44.43	45.29	46.15	47.07	47.97	48.77	49.54
20	44.56	45.42	46.27	47.20	48.10	48.90	49.67
21	44.69	45.55	46.41	47.34	48.24	49.04	49.81
22	44.84	45.70	46.56	47.48	48.39	49.18	49.96
23	45.00	45.86	46.71	47.64	48.54	49.34	50.11
24	45.18	46.03	46.88	47.80	48.71	49.50	50.27
25	45.36	46.21	47.05	47.97	48.86	49.65	50.42
26	45.53	46.38	47.21	48.12	49.01	49.79	50.55
27	45.70	46.54	47.36	48.26	49.14	49.92	50.67
28	45.86	46.68	47.50	48.38	49.25	50.02	50.76
29	46.00	46.81	47.62	48.49	49.35	50.10	50.84
30	46.13	46.93	47.72	48.58	49.43	50.17	50.90
31	46.25	47.04	47.82	48.67	49.50	50.24	50.95
32	46.37	47.14	47.91	48.75	49.57	50.29	50.99
33	46.49	47.25	48.00	48.83	49.63	50.35	51.04
34	46.60	47.35	48.09	48.90	49.70	50.40	51.08
35	46.71	47.45	48.18	48.98	49.76	50.45	51.12
36	46.82	47.55	48.27	49.05	49.82	50.50	51.16

By Wuhan University Children’s Digital Health and Data Center (CDHDC)

The Postnatal Growth Reference for Preterm Infants

WUHAN UNIVERSITY

BMI(28w boys)

Corrected age(months)	Centiles						
	3rd	10rd	25rd	50rd	75rd	90rd	97rd
0	10.66	11.63	12.43	13.79	14.60	16.44	17.81
1	11.59	12.65	13.77	15.04	16.36	17.60	18.86
2	12.28	13.33	14.41	15.66	16.94	18.14	19.36
3	12.89	13.91	14.97	16.17	17.42	18.58	19.76
4	13.37	14.36	15.39	16.56	17.77	18.89	20.03
5	13.72	14.69	15.68	16.82	17.99	19.07	20.17
6	13.94	14.88	15.85	16.95	18.08	19.13	20.20
7	14.05	14.97	15.91	16.99	18.09	19.12	20.16
8	14.08	14.98	15.90	16.96	18.04	19.04	20.06
9	14.05	14.93	15.85	16.88	17.94	18.92	19.92
10	13.98	14.85	15.75	16.76	17.81	18.77	19.75
11	13.89	14.74	15.62	16.62	17.65	18.59	19.55
12	13.78	14.62	15.49	16.47	17.47	18.40	19.34
13	13.66	14.49	15.34	16.3	17.29	18.20	19.13
14	13.54	14.35	15.19	16.14	17.11	18.01	18.92
15	13.42	14.22	15.04	15.97	16.93	17.81	18.71
16	13.30	14.09	14.90	15.81	16.75	17.62	18.50
17	13.21	13.98	14.77	15.67	16.59	17.44	18.30
18	13.13	13.89	14.66	15.55	16.45	17.28	18.13
19	13.07	13.81	14.58	15.44	16.33	17.14	17.97
20	13.02	13.75	14.50	15.35	16.22	17.02	17.83
21	12.98	13.70	14.44	15.27	16.13	16.92	17.72
22	12.94	13.65	14.38	15.21	16.05	16.83	17.62
23	12.90	13.60	14.33	15.14	15.98	16.75	17.53
24	12.85	13.55	14.27	15.09	15.92	16.69	17.47
25	12.79	13.49	14.21	15.03	15.86	16.63	17.41
26	12.72	13.42	14.15	14.97	15.81	16.58	17.36
27	12.64	13.35	14.08	14.91	15.76	16.54	17.33
28	12.56	13.28	14.02	14.86	15.71	16.50	17.30
29	12.48	13.21	13.96	14.81	15.67	16.48	17.29
30	12.40	13.14	13.90	14.76	15.65	16.47	17.30
31	12.32	13.07	13.85	14.73	15.64	16.47	17.32
32	12.24	13.01	13.80	14.71	15.63	16.49	17.36
33	12.15	12.94	13.76	14.69	15.64	16.52	17.41
34	12.07	12.88	13.71	14.67	15.65	16.55	17.47
35	11.98	12.81	13.67	14.65	15.66	16.59	17.54
36	11.89	12.74	13.63	14.63	15.67	16.63	17.61

By Wuhan University Children’s Digital Health and Data Center (CDHDC)

The Postnatal Growth Reference for Preterm Infants

WUHAN UNIVERSITY

Length (<28w boys))

Corrected age(months)	Centiles						
	3rd	10rd	25rd	50rd	75rd	90rd	97rd
0	44.99	46.80	48.43	49.95	52.38	53.00	55.07
1	48.82	50.52	52.18	54.01	55.83	57.51	59.24
2	51.99	53.75	55.48	57.38	59.30	61.06	62.88
3	54.92	56.71	58.47	60.40	62.35	64.14	65.98
4	57.60	59.40	61.17	63.12	65.07	66.87	68.72
5	60.01	61.80	63.56	65.49	67.44	69.23	71.06
6	62.12	63.89	65.63	67.54	69.46	71.22	73.03
7	63.97	65.71	67.42	69.30	71.18	72.92	74.70
8	65.59	67.30	68.98	70.83	72.68	74.39	76.14
9	67.00	68.69	70.36	72.18	74.01	75.69	77.42
10	68.26	69.94	71.59	73.39	75.21	76.87	78.59
11	69.40	71.07	72.71	74.51	76.31	77.98	79.68
12	70.44	72.11	73.76	75.56	77.36	79.02	80.73
13	71.42	73.10	74.75	76.56	78.37	80.04	81.75
14	72.35	74.04	75.70	77.52	79.35	81.03	82.75
15	73.25	74.95	76.63	78.46	80.30	82.00	83.74
16	74.11	75.84	77.53	79.39	81.25	82.97	84.73
17	74.95	76.70	78.42	80.31	82.20	83.94	85.72
18	75.75	77.53	79.28	81.21	83.14	84.91	86.73
19	76.49	78.32	80.12	82.09	84.06	85.88	87.75
20	77.21	79.08	80.93	82.95	84.98	86.85	88.77
21	77.89	79.81	81.71	83.79	85.88	87.80	89.77
22	78.54	80.52	82.47	84.60	86.74	88.72	90.74
23	79.16	81.19	83.19	85.38	87.58	89.60	91.68
24	79.75	81.83	83.89	86.13	88.39	90.47	92.60
25	80.32	82.46	84.56	86.87	89.18	91.32	93.50
26	80.88	83.07	85.23	87.59	89.96	92.15	94.40
27	81.43	83.68	85.88	88.30	90.73	92.96	95.26
28	82.00	84.28	86.53	88.99	91.47	93.75	96.09
29	82.57	84.89	87.17	89.68	92.19	94.50	96.88
30	83.16	85.51	87.82	90.35	92.90	95.24	97.65
31	83.77	86.14	88.47	91.03	93.60	95.96	98.39
32	84.39	86.77	89.13	91.70	94.29	96.68	99.12
33	85.02	87.42	89.79	92.38	94.99	97.39	99.85
34	85.66	88.07	90.45	93.06	95.68	98.09	100.57
35	86.31	88.72	91.12	93.74	96.37	98.80	101.29
36	86.96	89.39	91.83	94.46	97.06	99.50	102.00

The Postnatal Growth Reference for Preterm Infants

WUHAN UNIVERSITY

Weight(<28w boys))

Corrected age(months)	Centiles						
	3rd	10rd	25rd	50rd	75rd	90rd	97rd
0	2.17	2.50	2.85	3.26	3.69	4.09	4.50
1	2.87	3.28	3.71	4.21	4.74	5.23	5.73
2	3.53	4.00	4.50	5.08	5.69	6.26	6.85
3	4.16	4.67	5.22	5.85	6.52	7.14	7.77
4	4.73	5.27	5.85	6.52	7.21	7.86	8.52
5	5.23	5.79	6.38	7.07	7.78	8.44	9.11
6	5.67	6.24	6.84	7.52	8.24	8.90	9.57
7	6.06	6.63	7.22	7.91	8.61	9.27	9.94
8	6.40	6.96	7.56	8.24	8.94	9.59	10.25
9	6.70	7.26	7.85	8.53	9.22	9.87	10.52
10	6.97	7.53	8.11	8.79	9.48	10.12	10.77
11	7.20	7.77	8.35	9.02	9.72	10.36	11.00
12	7.42	7.98	8.57	9.25	9.94	10.58	11.23
13	7.61	8.18	8.78	9.46	10.16	10.80	11.46
14	7.79	8.37	8.97	9.65	10.36	11.02	11.68
15	7.95	8.53	9.14	9.84	10.56	11.22	11.89
16	8.10	8.69	9.31	10.02	10.75	11.42	12.10
17	8.24	8.84	9.47	10.19	10.93	11.61	12.30
18	8.38	8.99	9.63	10.36	11.11	11.80	12.50
19	8.51	9.14	9.78	10.52	11.29	11.99	12.70
20	8.65	9.29	9.94	10.70	11.47	12.18	12.91
21	8.79	9.44	10.10	10.87	11.65	12.38	13.11
22	8.93	9.59	10.26	11.04	11.83	12.57	13.31
23	9.07	9.73	10.42	11.20	12.01	12.76	13.51
24	9.20	9.87	10.57	11.37	12.19	12.94	13.71
25	9.33	10.01	10.71	11.52	12.35	13.12	13.90
26	9.44	10.13	10.85	11.67	12.52	13.30	14.09
27	9.56	10.26	10.99	11.82	12.67	13.46	14.26
28	9.67	10.38	11.11	11.96	12.82	13.62	14.43
29	9.78	10.50	11.24	12.10	12.97	13.78	14.60
30	9.90	10.62	11.38	12.24	13.12	13.94	14.77
31	10.02	10.75	11.51	12.38	13.27	14.10	14.93
32	10.15	10.89	11.65	12.53	13.43	14.26	15.10
33	10.28	11.02	11.80	12.68	13.59	14.43	15.28
34	10.42	11.17	11.95	12.84	13.75	14.60	15.45
35	10.55	11.31	12.10	12.99	13.92	14.77	15.63
36	10.69	11.45	12.24	13.15	14.08	14.94	15.80

By Wuhan University Children’s Digital Health and Data Center (CDHDC)

The Postnatal Growth Reference for Preterm Infants

Head circumference(<28w boys))

Corrected age(months)	Centiles						
	3rd	10rd	25rd	50rd	75rd	90rd	97rd
0	31.49	32.46	33.42	34.25	35.28	36.21	37.14
1	33.81	34.77	35.71	36.73	37.74	38.65	39.56
2	35.21	36.20	37.17	38.22	39.26	40.20	41.15
3	36.47	37.46	38.43	39.49	40.53	41.48	42.43
4	37.60	38.59	39.56	40.61	41.66	42.61	43.56
5	38.57	39.56	40.52	41.57	42.62	43.56	44.51
6	39.38	40.36	41.32	42.36	43.40	44.34	45.28
7	40.04	41.01	41.96	42.99	44.02	44.94	45.88
8	40.59	41.54	42.47	43.49	44.50	45.41	46.33
9	41.03	41.97	42.88	43.88	44.87	45.76	46.67
10	41.40	42.32	43.22	44.19	45.16	46.04	46.93
11	41.72	42.62	43.50	44.45	45.40	46.27	47.13
12	42.00	42.88	43.74	44.68	45.61	46.46	47.31
13	42.26	43.12	43.97	44.89	45.81	46.64	47.47
14	42.50	43.35	44.18	45.09	45.99	46.81	47.63
15	42.73	43.57	44.39	45.29	46.18	46.98	47.80
16	42.94	43.77	44.59	45.48	46.36	47.16	47.97
17	43.13	43.96	44.77	45.66	46.54	47.33	48.14
18	43.30	44.13	44.94	45.82	46.70	47.49	48.30
19	43.45	44.28	45.09	45.97	46.85	47.65	48.45
20	43.59	44.42	45.23	46.12	47.00	47.80	48.60
21	43.72	44.55	45.37	46.26	47.14	47.94	48.75
22	43.83	44.67	45.49	46.39	47.28	48.08	48.90
23	43.94	44.78	45.61	46.51	47.40	48.21	49.03
24	44.03	44.88	45.71	46.62	47.52	48.33	49.16
25	44.12	44.97	45.81	46.72	47.62	48.44	49.27
26	44.20	45.05	45.89	46.80	47.71	48.53	49.36
27	44.27	45.12	45.96	46.87	47.78	48.60	49.42
28	44.34	45.19	46.02	46.93	47.83	48.65	49.47
29	44.41	45.26	46.09	46.99	47.88	48.70	49.52
30	44.49	45.33	46.15	47.05	47.94	48.75	49.56
31	44.58	45.41	46.23	47.12	48.00	48.80	49.61
32	44.68	45.50	46.31	47.19	48.07	48.86	49.66
33	44.78	45.60	46.40	47.27	48.14	48.93	49.72
34	44.89	45.70	46.50	47.36	48.22	49.00	49.78
35	45.01	45.81	46.60	47.45	48.30	49.07	49.85
36	45.13	45.92	46.69	47.54	48.38	49.14	49.91

By Wuhan University Children’s Digital Health and Data Center (CDHDC)

The Postnatal Growth Reference for Preterm Infants

WUHAN UNIVERSITY

BMI(<28w boys))

Corrected age(months)	Centiles						
	3rd	10rd	25rd	50rd	75rd	90rd	97rd
0	10.36	11.53	12.74	13.45	15.54	16.86	18.20
1	11.02	12.17	13.35	14.69	16.07	17.35	18.65
2	11.65	12.77	13.92	15.23	16.57	17.82	19.08
3	12.19	13.29	14.42	15.69	17.00	18.20	19.43
4	12.63	13.70	14.80	16.04	17.31	18.48	19.67
5	12.96	14.00	15.07	16.27	17.49	18.63	19.78
6	13.21	14.21	15.24	16.40	17.58	18.67	19.78
7	13.37	14.34	15.33	16.45	17.59	18.64	19.71
8	13.48	14.42	15.38	16.46	17.56	18.57	19.60
9	13.54	14.45	15.38	16.42	17.49	18.47	19.46
10	13.57	14.45	15.35	16.37	17.41	18.36	19.32
11	13.57	14.43	15.31	16.30	17.31	18.24	19.18
12	13.55	14.40	15.26	16.23	17.22	18.12	19.04
13	13.53	14.36	15.20	16.15	17.12	18.01	18.91
14	13.49	14.31	15.14	16.07	17.02	17.89	18.77
15	13.45	14.25	15.07	15.98	16.91	17.77	18.63
16	13.40	14.19	14.99	15.88	16.80	17.63	18.48
17	13.36	14.12	14.90	15.78	16.67	17.49	18.32
18	13.31	14.06	14.83	15.68	16.55	17.35	18.16
19	13.28	14.01	14.76	15.60	16.45	17.23	18.02
20	13.26	13.97	14.70	15.52	16.36	17.12	17.89
21	13.24	13.94	14.66	15.46	16.28	17.03	17.78
22	13.23	13.92	14.63	15.41	16.21	16.95	17.69
23	13.22	13.90	14.60	15.37	16.16	16.88	17.61
24	13.21	13.88	14.57	15.33	16.11	16.82	17.54
25	13.19	13.85	14.53	15.28	16.05	16.75	17.46
26	13.16	13.81	14.48	15.22	15.98	16.67	17.37
27	13.11	13.76	14.42	15.16	15.91	16.59	17.29
28	13.06	13.70	14.35	15.09	15.83	16.51	17.20
29	13.00	13.64	14.29	15.02	15.76	16.44	17.13
30	12.94	13.58	14.23	14.96	15.70	16.38	17.07
31	12.89	13.53	14.18	14.91	15.66	16.34	17.02
32	12.84	13.49	14.14	14.88	15.62	16.30	16.99
33	12.80	13.45	14.11	14.85	15.60	16.29	16.98
34	12.76	13.41	14.08	14.83	15.59	16.28	16.98
35	12.71	13.38	14.05	14.81	15.58	16.28	16.99
36	12.67	13.34	14.02	14.79	15.57	16.28	17.01
