# Supplementary material for: The growth diversity of preterm infants at 0–36 months corrected age in China: a real-world observational study
Source: Front Pediatr. 2025 Jan 31;13:1506244. doi: 10.3389/fped.2025.1506244 (PMC11825782; doi:10.3389/fped.2025.1506244)
Supplement: Supplementary file 4 [file Datasheet4.pdf]

# The Postnatal Growth Reference for Preterm Infants

WUHAN UNIVERSITY

## Length (36w girls)

| Corrected age(months) | Centiles | | | | | | |
|---|---|---|---|---|---|---|---|
| | 3rd | 10rd | 25rd | 50rd | 75rd | 90rd | 97rd |
| 0 | 46.72 | 48.79 | 50.00 | 51.40 | 53.00 | 54.00 | 55.30 |
| 1 | 50.97 | 52.41 | 53.75 | 55.15 | 56.50 | 57.73 | 58.98 |
| 2 | 53.93 | 55.42 | 56.81 | 58.26 | 59.67 | 60.94 | 62.24 |
| 3 | 56.62 | 58.15 | 59.58 | 61.07 | 62.52 | 63.83 | 65.17 |
| 4 | 58.98 | 60.55 | 62.00 | 63.52 | 65.00 | 66.34 | 67.71 |
| 5 | 61.03 | 62.62 | 64.10 | 65.65 | 67.15 | 68.52 | 69.91 |
| 6 | 62.79 | 64.41 | 65.90 | 67.47 | 69.00 | 70.39 | 71.80 |
| 7 | 64.33 | 65.96 | 67.48 | 69.07 | 70.62 | 72.02 | 73.46 |
| 8 | 65.69 | 67.34 | 68.89 | 70.50 | 72.07 | 73.50 | 74.95 |
| 9 | 66.92 | 68.61 | 70.17 | 71.81 | 73.41 | 74.86 | 76.34 |
| 10 | 68.07 | 69.79 | 71.38 | 73.05 | 74.67 | 76.15 | 77.65 |
| 11 | 69.16 | 70.91 | 72.53 | 74.23 | 75.88 | 77.38 | 78.92 |
| 12 | 70.20 | 71.98 | 73.63 | 75.37 | 77.05 | 78.58 | 80.14 |
| 13 | 71.21 | 73.02 | 74.71 | 76.48 | 78.20 | 79.76 | 81.35 |
| 14 | 72.19 | 74.04 | 75.76 | 77.56 | 79.32 | 80.91 | 82.53 |
| 15 | 73.15 | 75.04 | 76.79 | 78.63 | 80.42 | 82.04 | 83.70 |
| 16 | 74.09 | 76.01 | 77.81 | 79.68 | 81.51 | 83.16 | 84.85 |
| 17 | 75.01 | 76.97 | 78.80 | 80.71 | 82.57 | 84.25 | 85.97 |
| 18 | 75.90 | 77.90 | 79.76 | 81.70 | 83.60 | 85.31 | 87.06 |
| 19 | 76.76 | 78.80 | 80.69 | 82.67 | 84.59 | 86.34 | 88.11 |
| 20 | 77.60 | 79.67 | 81.59 | 83.60 | 85.56 | 87.33 | 89.14 |
| 21 | 78.42 | 80.52 | 82.48 | 84.52 | 86.51 | 88.30 | 90.14 |
| 22 | 79.22 | 81.36 | 83.34 | 85.41 | 87.43 | 89.25 | 91.11 |
| 23 | 80.01 | 82.17 | 84.18 | 86.29 | 88.33 | 90.18 | 92.07 |
| 24 | 80.77 | 82.97 | 85.01 | 87.14 | 89.22 | 91.09 | 93.01 |
| 25 | 81.51 | 83.74 | 85.81 | 87.98 | 90.08 | 91.98 | 93.92 |
| 26 | 82.24 | 84.50 | 86.60 | 88.80 | 90.93 | 92.86 | 94.82 |
| 27 | 82.96 | 85.25 | 87.38 | 89.60 | 91.76 | 93.71 | 95.70 |
| 28 | 83.66 | 85.98 | 88.14 | 90.39 | 92.57 | 94.55 | 96.56 |
| 29 | 84.35 | 86.70 | 88.88 | 91.16 | 93.37 | 95.37 | 97.41 |
| 30 | 85.03 | 87.40 | 89.61 | 91.92 | 94.16 | 96.18 | 98.24 |
| 31 | 85.68 | 88.09 | 90.33 | 92.66 | 94.93 | 96.98 | 99.06 |
| 32 | 86.33 | 88.77 | 91.03 | 93.39 | 95.69 | 97.76 | 99.87 |
| 33 | 86.96 | 89.43 | 91.72 | 94.11 | 96.44 | 98.54 | 100.67 |
| 34 | 87.59 | 90.09 | 92.41 | 94.83 | 97.18 | 99.31 | 101.47 |
| 35 | 88.22 | 90.75 | 93.10 | 95.55 | 97.92 | 100.07 | 102.26 |
| 36 | 88.85 | 91.41 | 93.78 | 96.26 | 98.67 | 100.84 | 103.05 |

By Wuhan University Children's Digital Health and Data Center (CDHDC)

# The Postnatal Growth Reference for Preterm Infants

WUHAN UNIVERSITY

## Weight (36w girls)

| Corrected age(months) | Centiles | | | | | | |
|---|---|---|---|---|---|---|---|
| | **3rd** | **10rd** | **25rd** | **50rd** | **75rd** | **90rd** | **97rd** |
| 0 | 2.59 | 2.90 | 3.20 | 3.50 | 3.90 | 4.19 | 4.50 |
| 1 | 3.54 | 3.90 | 4.25 | 4.66 | 5.07 | 5.48 | 5.91 |
| 2 | 4.27 | 4.69 | 5.12 | 5.60 | 6.09 | 6.57 | 7.09 |
| 3 | 4.90 | 5.38 | 5.87 | 6.41 | 6.97 | 7.51 | 8.09 |
| 4 | 5.43 | 5.96 | 6.48 | 7.07 | 7.69 | 8.28 | 8.91 |
| 5 | 5.87 | 6.43 | 6.99 | 7.62 | 8.27 | 8.90 | 9.57 |
| 6 | 6.22 | 6.81 | 7.40 | 8.05 | 8.73 | 9.39 | 10.10 |
| 7 | 6.52 | 7.12 | 7.73 | 8.40 | 9.11 | 9.78 | 10.51 |
| 8 | 6.77 | 7.39 | 8.01 | 8.70 | 9.42 | 10.11 | 10.85 |
| 9 | 6.98 | 7.61 | 8.24 | 8.95 | 9.68 | 10.38 | 11.14 |
| 10 | 7.17 | 7.82 | 8.46 | 9.17 | 9.91 | 10.63 | 11.39 |
| 11 | 7.35 | 8.00 | 8.65 | 9.38 | 10.13 | 10.85 | 11.63 |
| 12 | 7.52 | 8.18 | 8.83 | 9.57 | 10.33 | 11.06 | 11.85 |
| 13 | 7.67 | 8.34 | 9.01 | 9.75 | 10.53 | 11.27 | 12.06 |
| 14 | 7.82 | 8.50 | 9.18 | 9.94 | 10.72 | 11.47 | 12.28 |
| 15 | 7.98 | 8.67 | 9.35 | 10.12 | 10.91 | 11.67 | 12.49 |
| 16 | 8.13 | 8.83 | 9.53 | 10.31 | 11.11 | 11.89 | 12.72 |
| 17 | 8.29 | 9.00 | 9.71 | 10.50 | 11.32 | 12.10 | 12.94 |
| 18 | 8.45 | 9.17 | 9.89 | 10.69 | 11.52 | 12.32 | 13.17 |
| 19 | 8.61 | 9.34 | 10.07 | 10.88 | 11.73 | 12.53 | 13.40 |
| 20 | 8.76 | 9.51 | 10.25 | 11.08 | 11.93 | 12.75 | 13.63 |
| 21 | 8.92 | 9.68 | 10.43 | 11.27 | 12.14 | 12.98 | 13.87 |
| 22 | 9.08 | 9.85 | 10.61 | 11.47 | 12.35 | 13.20 | 14.12 |
| 23 | 9.23 | 10.02 | 10.80 | 11.67 | 12.57 | 13.43 | 14.36 |
| 24 | 9.38 | 10.18 | 10.98 | 11.87 | 12.79 | 13.67 | 14.62 |
| 25 | 9.53 | 10.35 | 11.16 | 12.06 | 13.00 | 13.90 | 14.87 |
| 26 | 9.67 | 10.51 | 11.34 | 12.26 | 13.22 | 14.14 | 15.13 |
| 27 | 9.81 | 10.67 | 11.51 | 12.46 | 13.44 | 14.39 | 15.40 |
| 28 | 9.95 | 10.82 | 11.69 | 12.66 | 13.67 | 14.63 | 15.67 |
| 29 | 10.09 | 10.98 | 11.87 | 12.86 | 13.89 | 14.88 | 15.94 |
| 30 | 10.22 | 11.14 | 12.04 | 13.06 | 14.11 | 15.12 | 16.21 |
| 31 | 10.36 | 11.29 | 12.22 | 13.26 | 14.34 | 15.38 | 16.49 |
| 32 | 10.49 | 11.44 | 12.40 | 13.46 | 14.57 | 15.63 | 16.77 |
| 33 | 10.62 | 11.60 | 12.57 | 13.66 | 14.79 | 15.88 | 17.05 |
| 34 | 10.75 | 11.75 | 12.75 | 13.86 | 15.02 | 16.13 | 17.33 |
| 35 | 10.88 | 11.91 | 12.92 | 14.07 | 15.25 | 16.39 | 17.62 |
| 36 | 11.02 | 12.06 | 13.10 | 14.27 | 15.48 | 16.65 | 17.90 |

By Wuhan University Children’s Digital Health and Data Center (CDHDC)

# The Postnatal Growth Reference for Preterm Infants

WUHAN UNIVERSITY

## Head circumference (36w girls)

| Corrected age(months) | Centiles | | | | | | |
|---|---|---|---|---|---|---|---|
| | 3rd | 10rd | 25rd | 50rd | 75rd | 90rd | 97rd |
| 0 | 32.50 | 33.50 | 34.30 | 35.00 | 36.00 | 36.50 | 37.00 |
| 1 | 34.75 | 35.54 | 36.27 | 37.02 | 37.77 | 38.46 | 39.19 |
| 2 | 36.09 | 36.91 | 37.65 | 38.42 | 39.18 | 39.89 | 40.63 |
| 3 | 37.31 | 38.14 | 38.89 | 39.68 | 40.46 | 41.17 | 41.94 |
| 4 | 38.37 | 39.21 | 39.97 | 40.78 | 41.56 | 42.29 | 43.07 |
| 5 | 39.27 | 40.12 | 40.90 | 41.71 | 42.51 | 43.25 | 44.03 |
| 6 | 40.02 | 40.88 | 41.67 | 42.49 | 43.30 | 44.05 | 44.84 |
| 7 | 40.65 | 41.52 | 42.31 | 43.14 | 43.96 | 44.71 | 45.51 |
| 8 | 41.17 | 42.05 | 42.85 | 43.69 | 44.51 | 45.27 | 46.08 |
| 9 | 41.61 | 42.50 | 43.30 | 44.15 | 44.98 | 45.75 | 46.56 |
| 10 | 41.98 | 42.87 | 43.69 | 44.54 | 45.37 | 46.15 | 46.97 |
| 11 | 42.30 | 43.20 | 44.02 | 44.87 | 45.72 | 46.50 | 47.32 |
| 12 | 42.58 | 43.48 | 44.31 | 45.17 | 46.01 | 46.80 | 47.63 |
| 13 | 42.83 | 43.74 | 44.56 | 45.43 | 46.28 | 47.07 | 47.90 |
| 14 | 43.06 | 43.97 | 44.80 | 45.66 | 46.52 | 47.31 | 48.15 |
| 15 | 43.27 | 44.18 | 45.01 | 45.88 | 46.74 | 47.53 | 48.37 |
| 16 | 43.47 | 44.38 | 45.22 | 46.09 | 46.94 | 47.74 | 48.58 |
| 17 | 43.66 | 44.58 | 45.41 | 46.28 | 47.13 | 47.93 | 48.77 |
| 18 | 43.85 | 44.76 | 45.59 | 46.46 | 47.32 | 48.11 | 48.95 |
| 19 | 44.03 | 44.94 | 45.77 | 46.64 | 47.49 | 48.28 | 49.12 |
| 20 | 44.19 | 45.10 | 45.93 | 46.80 | 47.65 | 48.45 | 49.28 |
| 21 | 44.36 | 45.26 | 46.09 | 46.96 | 47.81 | 48.60 | 49.44 |
| 22 | 44.51 | 45.42 | 46.24 | 47.11 | 47.96 | 48.75 | 49.59 |
| 23 | 44.66 | 45.56 | 46.39 | 47.25 | 48.10 | 48.89 | 49.73 |
| 24 | 44.80 | 45.70 | 46.53 | 47.39 | 48.24 | 49.03 | 49.87 |
| 25 | 44.93 | 45.84 | 46.66 | 47.53 | 48.38 | 49.16 | 50.00 |
| 26 | 45.07 | 45.97 | 46.79 | 47.66 | 48.51 | 49.29 | 50.13 |
| 27 | 45.19 | 46.09 | 46.92 | 47.78 | 48.63 | 49.42 | 50.26 |
| 28 | 45.31 | 46.21 | 47.04 | 47.90 | 48.75 | 49.54 | 50.38 |
| 29 | 45.43 | 46.33 | 47.16 | 48.02 | 48.87 | 49.66 | 50.49 |
| 30 | 45.54 | 46.44 | 47.27 | 48.13 | 48.98 | 49.77 | 50.61 |
| 31 | 45.64 | 46.55 | 47.38 | 48.24 | 49.09 | 49.88 | 50.72 |
| 32 | 45.75 | 46.66 | 47.49 | 48.35 | 49.20 | 49.99 | 50.83 |
| 33 | 45.86 | 46.76 | 47.59 | 48.46 | 49.31 | 50.11 | 50.95 |
| 34 | 45.96 | 46.87 | 47.70 | 48.57 | 49.42 | 50.22 | 51.06 |
| 35 | 46.07 | 46.98 | 47.81 | 48.68 | 49.54 | 50.33 | 51.17 |
| 36 | 46.18 | 47.09 | 47.92 | 48.79 | 49.65 | 50.44 | 51.29 |

By Wuhan University Children's Digital Health and Data Center (CDHDC)

# The Postnatal Growth Reference for Preterm Infants

WUHAN UNIVERSITY

## BMI (36w girls)

| Corrected age(months) | Centiles | | | | | | |
|---|---|---|---|---|---|---|---|
| | 3rd | 10rd | 25rd | 50rd | 75rd | 90rd | 97rd |
| 0 | 10.76 | 11.59 | 12.36 | 13.28 | 14.23 | 15.08 | 16.02 |
| 1 | 12.48 | 13.33 | 14.20 | 15.19 | 16.24 | 17.27 | 18.41 |
| 2 | 13.23 | 14.12 | 15.03 | 16.06 | 17.16 | 18.23 | 19.42 |
| 3 | 13.82 | 14.75 | 15.68 | 16.75 | 17.88 | 18.99 | 20.21 |
| 4 | 14.26 | 15.20 | 16.15 | 17.24 | 18.39 | 19.51 | 20.75 |
| 5 | 14.54 | 15.48 | 16.44 | 17.53 | 18.69 | 19.82 | 21.06 |
| 6 | 14.69 | 15.63 | 16.58 | 17.67 | 18.81 | 19.93 | 21.16 |
| 7 | 14.73 | 15.66 | 16.61 | 17.67 | 18.80 | 19.91 | 21.12 |
| 8 | 14.71 | 15.62 | 16.54 | 17.59 | 18.70 | 19.78 | 20.97 |
| 9 | 14.63 | 15.53 | 16.43 | 17.46 | 18.54 | 19.59 | 20.75 |
| 10 | 14.53 | 15.40 | 16.28 | 17.28 | 18.34 | 19.37 | 20.49 |
| 11 | 14.40 | 15.26 | 16.12 | 17.10 | 18.12 | 19.13 | 20.22 |
| 12 | 14.28 | 15.11 | 15.95 | 16.90 | 17.91 | 18.88 | 19.95 |
| 13 | 14.15 | 14.96 | 15.79 | 16.72 | 17.69 | 18.65 | 19.69 |
| 14 | 14.03 | 14.83 | 15.63 | 16.54 | 17.50 | 18.43 | 19.44 |
| 15 | 13.92 | 14.70 | 15.49 | 16.38 | 17.32 | 18.23 | 19.22 |
| 16 | 13.82 | 14.59 | 15.37 | 16.24 | 17.16 | 18.05 | 19.02 |
| 17 | 13.74 | 14.50 | 15.26 | 16.12 | 17.02 | 17.90 | 18.85 |
| 18 | 13.67 | 14.42 | 15.17 | 16.01 | 16.90 | 17.76 | 18.70 |
| 19 | 13.61 | 14.35 | 15.09 | 15.92 | 16.80 | 17.65 | 18.57 |
| 20 | 13.56 | 14.29 | 15.02 | 15.84 | 16.71 | 17.55 | 18.46 |
| 21 | 13.51 | 14.23 | 14.96 | 15.78 | 16.63 | 17.47 | 18.37 |
| 22 | 13.47 | 14.19 | 14.91 | 15.72 | 16.57 | 17.40 | 18.30 |
| 23 | 13.43 | 14.14 | 14.86 | 15.67 | 16.52 | 17.34 | 18.24 |
| 24 | 13.39 | 14.10 | 14.82 | 15.63 | 16.47 | 17.30 | 18.19 |
| 25 | 13.35 | 14.06 | 14.78 | 15.59 | 16.43 | 17.26 | 18.15 |
| 26 | 13.31 | 14.02 | 14.74 | 15.55 | 16.40 | 17.22 | 18.12 |
| 27 | 13.27 | 13.98 | 14.70 | 15.52 | 16.37 | 17.20 | 18.10 |
| 28 | 13.23 | 13.95 | 14.67 | 15.49 | 16.34 | 17.18 | 18.08 |
| 29 | 13.19 | 13.91 | 14.64 | 15.46 | 16.33 | 17.16 | 18.08 |
| 30 | 13.15 | 13.89 | 14.62 | 15.45 | 16.32 | 17.16 | 18.08 |
| 31 | 13.13 | 13.86 | 14.60 | 15.44 | 16.31 | 17.16 | 18.09 |
| 32 | 13.10 | 13.84 | 14.59 | 15.43 | 16.32 | 17.18 | 18.12 |
| 33 | 13.08 | 13.82 | 14.58 | 15.43 | 16.32 | 17.19 | 18.15 |
| 34 | 13.05 | 13.81 | 14.57 | 15.43 | 16.34 | 17.22 | 18.18 |
| 35 | 13.04 | 13.80 | 14.57 | 15.44 | 16.35 | 17.24 | 18.22 |
| 36 | 13.02 | 13.79 | 14.56 | 15.45 | 16.37 | 17.27 | 18.26 |

# The Postnatal Growth Reference for Preterm Infants

WUHAN UNIVERSITY

## Length (35w girls)

| Corrected age(months) | Centiles | | | | | | |
|---|---|---|---|---|---|---|---|
| | 3rd | 10rd | 25rd | 50rd | 75rd | 90rd | 97rd |
| 0 | 46.50 | 48.00 | 49.50 | 51.00 | 52.50 | 53.70 | 54.80 |
| 1 | 50.60 | 52.08 | 53.47 | 54.93 | 56.35 | 57.63 | 58.93 |
| 2 | 53.62 | 55.15 | 56.59 | 58.09 | 59.56 | 60.88 | 62.23 |
| 3 | 56.36 | 57.92 | 59.39 | 60.93 | 62.44 | 63.80 | 65.18 |
| 4 | 58.76 | 60.35 | 61.84 | 63.42 | 64.95 | 66.34 | 67.75 |
| 5 | 60.83 | 62.45 | 63.96 | 65.56 | 67.12 | 68.53 | 69.96 |
| 6 | 62.61 | 64.25 | 65.79 | 67.41 | 68.99 | 70.42 | 71.87 |
| 7 | 64.17 | 65.83 | 67.38 | 69.02 | 70.62 | 72.06 | 73.54 |
| 8 | 65.56 | 67.23 | 68.80 | 70.46 | 72.08 | 73.54 | 75.03 |
| 9 | 66.83 | 68.52 | 70.12 | 71.80 | 73.43 | 74.92 | 76.43 |
| 10 | 68.01 | 69.73 | 71.35 | 73.05 | 74.72 | 76.23 | 77.76 |
| 11 | 69.12 | 70.88 | 72.52 | 74.26 | 75.95 | 77.48 | 79.05 |
| 12 | 70.18 | 71.97 | 73.65 | 75.41 | 77.14 | 78.70 | 80.29 |
| 13 | 71.20 | 73.03 | 74.73 | 76.54 | 78.30 | 79.89 | 81.51 |
| 14 | 72.20 | 74.06 | 75.80 | 77.63 | 79.42 | 81.05 | 82.70 |
| 15 | 73.18 | 75.06 | 76.84 | 78.71 | 80.53 | 82.18 | 83.86 |
| 16 | 74.14 | 76.06 | 77.86 | 79.76 | 81.61 | 83.29 | 84.99 |
| 17 | 75.08 | 77.03 | 78.86 | 80.78 | 82.66 | 84.37 | 86.10 |
| 18 | 76.00 | 77.98 | 79.83 | 81.79 | 83.69 | 85.42 | 87.18 |
| 19 | 76.90 | 78.91 | 80.79 | 82.77 | 84.70 | 86.45 | 88.23 |
| 20 | 77.79 | 79.82 | 81.73 | 83.73 | 85.69 | 87.46 | 89.26 |
| 21 | 78.67 | 80.73 | 82.65 | 84.68 | 86.66 | 88.45 | 90.28 |
| 22 | 79.54 | 81.62 | 83.56 | 85.62 | 87.62 | 89.43 | 91.28 |
| 23 | 80.39 | 82.49 | 84.46 | 86.54 | 88.56 | 90.39 | 92.26 |
| 24 | 81.22 | 83.34 | 85.34 | 87.44 | 89.49 | 91.34 | 93.23 |
| 25 | 82.03 | 84.18 | 86.19 | 88.32 | 90.39 | 92.27 | 94.17 |
| 26 | 82.81 | 84.99 | 87.03 | 89.18 | 91.28 | 93.17 | 95.10 |
| 27 | 83.57 | 85.77 | 87.84 | 90.01 | 92.14 | 94.06 | 96.01 |
| 28 | 84.30 | 86.53 | 88.62 | 90.83 | 92.97 | 94.92 | 96.90 |
| 29 | 85.00 | 87.26 | 89.38 | 91.61 | 93.79 | 95.76 | 97.76 |
| 30 | 85.68 | 87.97 | 90.11 | 92.37 | 94.58 | 96.57 | 98.59 |
| 31 | 86.33 | 88.65 | 90.82 | 93.11 | 95.34 | 97.36 | 99.41 |
| 32 | 86.96 | 89.31 | 91.51 | 93.82 | 96.08 | 98.12 | 100.20 |
| 33 | 87.56 | 89.94 | 92.17 | 94.52 | 96.81 | 98.87 | 100.98 |
| 34 | 88.16 | 90.57 | 92.83 | 95.20 | 97.52 | 99.61 | 101.74 |
| 35 | 88.75 | 91.19 | 93.47 | 95.88 | 98.22 | 100.34 | 102.49 |
| 36 | 89.34 | 91.81 | 94.12 | 96.55 | 98.93 | 101.07 | 103.25 |

By Wuhan University Children’s Digital Health and Data Center (CDHDC)

# The Postnatal Growth Reference for Preterm Infants

WUHAN UNIVERSITY

## Weight (35w girls)

| Corrected age(months) | Centiles | | | | | | |
|---|---|---|---|---|---|---|---|
| | 3rd | 10rd | 25rd | 50rd | 75rd | 90rd | 97rd |
| 0 | 2.50 | 2.80 | 3.10 | 3.46 | 3.80 | 4.03 | 4.37 |
| 1 | 3.45 | 3.82 | 4.19 | 4.60 | 5.03 | 5.44 | 5.88 |
| 2 | 4.19 | 4.62 | 5.06 | 5.55 | 6.05 | 6.54 | 7.05 |
| 3 | 4.82 | 5.31 | 5.80 | 6.35 | 6.92 | 7.46 | 8.04 |
| 4 | 5.35 | 5.89 | 6.42 | 7.02 | 7.64 | 8.23 | 8.85 |
| 5 | 5.80 | 6.36 | 6.93 | 7.56 | 8.22 | 8.85 | 9.51 |
| 6 | 6.16 | 6.75 | 7.34 | 8.00 | 8.69 | 9.34 | 10.03 |
| 7 | 6.46 | 7.07 | 7.68 | 8.36 | 9.07 | 9.74 | 10.45 |
| 8 | 6.72 | 7.34 | 7.97 | 8.66 | 9.38 | 10.06 | 10.79 |
| 9 | 6.95 | 7.58 | 8.22 | 8.92 | 9.66 | 10.35 | 11.08 |
| 10 | 7.16 | 7.80 | 8.44 | 9.16 | 9.90 | 10.60 | 11.34 |
| 11 | 7.35 | 8.00 | 8.65 | 9.37 | 10.12 | 10.83 | 11.59 |
| 12 | 7.52 | 8.18 | 8.84 | 9.57 | 10.33 | 11.05 | 11.81 |
| 13 | 7.68 | 8.35 | 9.02 | 9.77 | 10.54 | 11.27 | 12.04 |
| 14 | 7.84 | 8.52 | 9.20 | 9.96 | 10.74 | 11.48 | 12.27 |
| 15 | 7.99 | 8.68 | 9.37 | 10.15 | 10.94 | 11.70 | 12.50 |
| 16 | 8.14 | 8.84 | 9.55 | 10.34 | 11.15 | 11.92 | 12.73 |
| 17 | 8.29 | 9.01 | 9.73 | 10.53 | 11.36 | 12.14 | 12.97 |
| 18 | 8.44 | 9.17 | 9.90 | 10.72 | 11.57 | 12.37 | 13.22 |
| 19 | 8.59 | 9.33 | 10.08 | 10.92 | 11.78 | 12.60 | 13.46 |
| 20 | 8.74 | 9.50 | 10.26 | 11.12 | 12.00 | 12.83 | 13.72 |
| 21 | 8.89 | 9.67 | 10.45 | 11.32 | 12.22 | 13.07 | 13.97 |
| 22 | 9.04 | 9.84 | 10.63 | 11.52 | 12.44 | 13.31 | 14.23 |
| 23 | 9.20 | 10.01 | 10.82 | 11.73 | 12.67 | 13.56 | 14.50 |
| 24 | 9.35 | 10.18 | 11.01 | 11.94 | 12.90 | 13.81 | 14.77 |
| 25 | 9.50 | 10.35 | 11.20 | 12.15 | 13.13 | 14.06 | 15.05 |
| 26 | 9.65 | 10.52 | 11.39 | 12.36 | 13.37 | 14.32 | 15.33 |
| 27 | 9.80 | 10.68 | 11.57 | 12.57 | 13.60 | 14.58 | 15.61 |
| 28 | 9.94 | 10.85 | 11.76 | 12.78 | 13.84 | 14.84 | 15.90 |
| 29 | 10.08 | 11.01 | 11.94 | 12.99 | 14.07 | 15.10 | 16.19 |
| 30 | 10.21 | 11.17 | 12.13 | 13.20 | 14.31 | 15.37 | 16.49 |
| 31 | 10.34 | 11.32 | 12.31 | 13.41 | 14.55 | 15.63 | 16.78 |
| 32 | 10.47 | 11.48 | 12.49 | 13.62 | 14.79 | 15.90 | 17.08 |
| 33 | 10.59 | 11.63 | 12.66 | 13.83 | 15.03 | 16.17 | 17.38 |
| 34 | 10.72 | 11.77 | 12.84 | 14.03 | 15.27 | 16.44 | 17.69 |
| 35 | 10.84 | 11.92 | 13.01 | 14.24 | 15.51 | 16.72 | 18.00 |
| 36 | 10.95 | 12.07 | 13.19 | 14.45 | 15.75 | 16.99 | 18.31 |

By Wuhan University Children's Digital Health and Data Center (CDHDC)

# The Postnatal Growth Reference for Preterm Infants

WUHAN UNIVERSITY

## Head circumference (35w girls)

| Corrected age(months) | Centiles | | | | | | |
|---|---|---|---|---|---|---|---|
| | 3rd | 10rd | 25rd | 50rd | 75rd | 90rd | 97rd |
| 0 | 32.50 | 33.30 | 34.00 | 35.00 | 35.80 | 36.50 | 37.00 |
| 1 | 34.55 | 35.37 | 36.13 | 36.92 | 37.70 | 38.42 | 39.18 |
| 2 | 35.95 | 36.79 | 37.55 | 38.36 | 39.15 | 39.88 | 40.65 |
| 3 | 37.21 | 38.05 | 38.83 | 39.64 | 40.44 | 41.18 | 41.97 |
| 4 | 38.30 | 39.15 | 39.93 | 40.76 | 41.57 | 42.32 | 43.11 |
| 5 | 39.22 | 40.08 | 40.87 | 41.70 | 42.52 | 43.28 | 44.08 |
| 6 | 39.98 | 40.85 | 41.66 | 42.49 | 43.32 | 44.09 | 44.89 |
| 7 | 40.62 | 41.50 | 42.31 | 43.15 | 43.99 | 44.76 | 45.58 |
| 8 | 41.15 | 42.04 | 42.85 | 43.71 | 44.55 | 45.33 | 46.16 |
| 9 | 41.59 | 42.49 | 43.31 | 44.18 | 45.03 | 45.82 | 46.65 |
| 10 | 41.97 | 42.87 | 43.71 | 44.58 | 45.44 | 46.23 | 47.07 |
| 11 | 42.29 | 43.20 | 44.04 | 44.92 | 45.79 | 46.59 | 47.44 |
| 12 | 42.57 | 43.49 | 44.33 | 45.22 | 46.09 | 46.90 | 47.75 |
| 13 | 42.81 | 43.74 | 44.59 | 45.48 | 46.35 | 47.17 | 48.03 |
| 14 | 43.03 | 43.96 | 44.82 | 45.71 | 46.59 | 47.41 | 48.27 |
| 15 | 43.24 | 44.17 | 45.03 | 45.92 | 46.81 | 47.63 | 48.49 |
| 16 | 43.44 | 44.37 | 45.23 | 46.12 | 47.00 | 47.82 | 48.69 |
| 17 | 43.63 | 44.56 | 45.41 | 46.30 | 47.18 | 48.00 | 48.86 |
| 18 | 43.81 | 44.73 | 45.58 | 46.47 | 47.35 | 48.17 | 49.02 |
| 19 | 43.99 | 44.90 | 45.75 | 46.64 | 47.51 | 48.32 | 49.18 |
| 20 | 44.16 | 45.07 | 45.91 | 46.79 | 47.66 | 48.47 | 49.32 |
| 21 | 44.32 | 45.23 | 46.07 | 46.94 | 47.81 | 48.61 | 49.45 |
| 22 | 44.48 | 45.39 | 46.22 | 47.09 | 47.95 | 48.74 | 49.58 |
| 23 | 44.64 | 45.54 | 46.36 | 47.23 | 48.08 | 48.87 | 49.71 |
| 24 | 44.79 | 45.68 | 46.50 | 47.36 | 48.21 | 49.00 | 49.83 |
| 25 | 44.92 | 45.81 | 46.63 | 47.49 | 48.34 | 49.13 | 49.96 |
| 26 | 45.06 | 45.94 | 46.76 | 47.62 | 48.46 | 49.25 | 50.08 |
| 27 | 45.18 | 46.07 | 46.88 | 47.74 | 48.58 | 49.37 | 50.19 |
| 28 | 45.30 | 46.19 | 47.00 | 47.86 | 48.70 | 49.48 | 50.31 |
| 29 | 45.41 | 46.30 | 47.12 | 47.97 | 48.82 | 49.60 | 50.43 |
| 30 | 45.52 | 46.41 | 47.23 | 48.08 | 48.93 | 49.71 | 50.54 |
| 31 | 45.62 | 46.51 | 47.33 | 48.19 | 49.04 | 49.83 | 50.66 |
| 32 | 45.72 | 46.61 | 47.43 | 48.30 | 49.15 | 49.94 | 50.77 |
| 33 | 45.81 | 46.71 | 47.53 | 48.40 | 49.26 | 50.05 | 50.89 |
| 34 | 45.90 | 46.80 | 47.63 | 48.50 | 49.36 | 50.16 | 51.00 |
| 35 | 45.98 | 46.89 | 47.73 | 48.61 | 49.47 | 50.27 | 51.12 |
| 36 | 46.07 | 46.99 | 47.83 | 48.71 | 49.58 | 50.38 | 51.23 |

By Wuhan University Children’s Digital Health and Data Center (CDHDC)

# The Postnatal Growth Reference for Preterm Infants

WUHAN UNIVERSITY

## BMI (35w girls)

| Corrected age(months) | Centiles | | | | | | |
|---|---|---|---|---|---|---|---|
| | 3rd | 10rd | 25rd | 50rd | 75rd | 90rd | 97rd |
| 0 | 10.52 | 11.51 | 12.38 | 13.24 | 14.05 | 14.88 | 15.52 |
| 1 | 12.36 | 13.23 | 14.10 | 15.09 | 16.13 | 17.16 | 18.31 |
| 2 | 13.15 | 14.05 | 14.96 | 15.98 | 17.07 | 18.13 | 19.31 |
| 3 | 13.77 | 14.70 | 15.63 | 16.68 | 17.79 | 18.88 | 20.09 |
| 4 | 14.23 | 15.17 | 16.11 | 17.17 | 18.29 | 19.39 | 20.61 |
| 5 | 14.52 | 15.46 | 16.40 | 17.46 | 18.58 | 19.68 | 20.89 |
| 6 | 14.68 | 15.61 | 16.55 | 17.60 | 18.70 | 19.79 | 20.99 |
| 7 | 14.73 | 15.66 | 16.57 | 17.61 | 18.69 | 19.76 | 20.93 |
| 8 | 14.72 | 15.62 | 16.52 | 17.53 | 18.59 | 19.63 | 20.78 |
| 9 | 14.66 | 15.54 | 16.42 | 17.40 | 18.44 | 19.44 | 20.56 |
| 10 | 14.57 | 15.43 | 16.28 | 17.24 | 18.24 | 19.22 | 20.30 |
| 11 | 14.47 | 15.30 | 16.13 | 17.06 | 18.04 | 18.99 | 20.04 |
| 12 | 14.35 | 15.16 | 15.97 | 16.88 | 17.83 | 18.75 | 19.77 |
| 13 | 14.22 | 15.02 | 15.81 | 16.70 | 17.63 | 18.53 | 19.53 |
| 14 | 14.10 | 14.89 | 15.66 | 16.53 | 17.44 | 18.32 | 19.30 |
| 15 | 13.99 | 14.76 | 15.52 | 16.38 | 17.27 | 18.14 | 19.10 |
| 16 | 13.88 | 14.64 | 15.39 | 16.24 | 17.12 | 17.98 | 18.92 |
| 17 | 13.78 | 14.54 | 15.28 | 16.11 | 16.99 | 17.83 | 18.77 |
| 18 | 13.69 | 14.44 | 15.18 | 16.01 | 16.87 | 17.71 | 18.64 |
| 19 | 13.61 | 14.35 | 15.09 | 15.91 | 16.77 | 17.60 | 18.52 |
| 20 | 13.54 | 14.27 | 15.01 | 15.82 | 16.68 | 17.51 | 18.43 |
| 21 | 13.47 | 14.20 | 14.93 | 15.75 | 16.60 | 17.43 | 18.34 |
| 22 | 13.41 | 14.14 | 14.87 | 15.69 | 16.54 | 17.37 | 18.28 |
| 23 | 13.36 | 14.09 | 14.82 | 15.64 | 16.49 | 17.32 | 18.23 |
| 24 | 13.31 | 14.04 | 14.77 | 15.59 | 16.44 | 17.28 | 18.19 |
| 25 | 13.26 | 14.00 | 14.73 | 15.55 | 16.41 | 17.24 | 18.16 |
| 26 | 13.21 | 13.95 | 14.69 | 15.51 | 16.37 | 17.21 | 18.13 |
| 27 | 13.17 | 13.92 | 14.65 | 15.48 | 16.35 | 17.19 | 18.12 |
| 28 | 13.13 | 13.88 | 14.62 | 15.45 | 16.33 | 17.17 | 18.11 |
| 29 | 13.10 | 13.85 | 14.60 | 15.44 | 16.31 | 17.17 | 18.11 |
| 30 | 13.07 | 13.83 | 14.58 | 15.43 | 16.31 | 17.18 | 18.13 |
| 31 | 13.05 | 13.81 | 14.57 | 15.42 | 16.32 | 17.19 | 18.16 |
| 32 | 13.02 | 13.80 | 14.57 | 15.43 | 16.33 | 17.22 | 18.19 |
| 33 | 13.01 | 13.79 | 14.56 | 15.44 | 16.35 | 17.25 | 18.24 |
| 34 | 12.99 | 13.78 | 14.57 | 15.45 | 16.38 | 17.29 | 18.29 |
| 35 | 12.98 | 13.78 | 14.57 | 15.47 | 16.41 | 17.33 | 18.35 |
| 36 | 12.96 | 13.77 | 14.58 | 15.48 | 16.44 | 17.37 | 18.41 |

# The Postnatal Growth Reference for Preterm Infants

WUHAN UNIVERSITY

## Length(34w girls)

| Corrected age(months) | Centiles | | | | | | |
|---|---|---|---|---|---|---|---|
| | 3rd | 10rd | 25rd | 50rd | 75rd | 90rd | 97rd |
| 0 | 46.70 | 48.00 | 49.60 | 51.00 | 52.50 | 53.80 | 55.00 |
| 1 | 50.00 | 51.67 | 53.17 | 54.70 | 56.17 | 57.48 | 58.81 |
| 2 | 53.01 | 54.73 | 56.28 | 57.87 | 59.38 | 60.74 | 62.12 |
| 3 | 55.75 | 57.51 | 59.09 | 60.72 | 62.27 | 63.66 | 65.08 |
| 4 | 58.17 | 59.95 | 61.56 | 63.21 | 64.79 | 66.21 | 67.65 |
| 5 | 60.27 | 62.08 | 63.70 | 65.37 | 66.97 | 68.41 | 69.87 |
| 6 | 62.10 | 63.92 | 65.56 | 67.25 | 68.86 | 70.32 | 71.81 |
| 7 | 63.70 | 65.53 | 67.19 | 68.90 | 70.53 | 72.01 | 73.51 |
| 8 | 65.11 | 66.97 | 68.65 | 70.38 | 72.03 | 73.53 | 75.05 |
| 9 | 66.39 | 68.27 | 69.98 | 71.73 | 73.41 | 74.93 | 76.47 |
| 10 | 67.56 | 69.48 | 71.21 | 72.99 | 74.70 | 76.24 | 77.82 |
| 11 | 68.68 | 70.62 | 72.39 | 74.20 | 75.94 | 77.51 | 79.11 |
| 12 | 69.75 | 71.73 | 73.52 | 75.36 | 77.13 | 78.72 | 80.35 |
| 13 | 70.80 | 72.81 | 74.63 | 76.50 | 78.29 | 79.91 | 81.56 |
| 14 | 71.84 | 73.88 | 75.72 | 77.61 | 79.43 | 81.07 | 82.74 |
| 15 | 72.88 | 74.94 | 76.80 | 78.71 | 80.55 | 82.20 | 83.89 |
| 16 | 73.92 | 75.99 | 77.87 | 79.80 | 81.65 | 83.32 | 85.03 |
| 17 | 74.93 | 77.02 | 78.92 | 80.86 | 82.73 | 84.42 | 86.14 |
| 18 | 75.92 | 78.03 | 79.94 | 81.90 | 83.79 | 85.49 | 87.22 |
| 19 | 76.88 | 79.00 | 80.93 | 82.91 | 84.81 | 86.53 | 88.28 |
| 20 | 77.81 | 79.95 | 81.89 | 83.89 | 85.81 | 87.54 | 89.31 |
| 21 | 78.70 | 80.87 | 82.83 | 84.85 | 86.79 | 88.54 | 90.32 |
| 22 | 79.56 | 81.75 | 83.73 | 85.77 | 87.73 | 89.50 | 91.31 |
| 23 | 80.38 | 82.59 | 84.60 | 86.66 | 88.65 | 90.44 | 92.27 |
| 24 | 81.16 | 83.40 | 85.43 | 87.52 | 89.53 | 91.34 | 93.19 |
| 25 | 81.90 | 84.17 | 86.23 | 88.35 | 90.38 | 92.22 | 94.09 |
| 26 | 82.61 | 84.92 | 87.01 | 89.15 | 91.22 | 93.08 | 94.97 |
| 27 | 83.30 | 85.64 | 87.76 | 89.94 | 92.03 | 93.91 | 95.84 |
| 28 | 83.98 | 86.35 | 88.50 | 90.71 | 92.83 | 94.74 | 96.69 |
| 29 | 84.65 | 87.05 | 89.23 | 91.46 | 93.61 | 95.54 | 97.52 |
| 30 | 85.30 | 87.74 | 89.94 | 92.20 | 94.37 | 96.33 | 98.33 |
| 31 | 85.95 | 88.41 | 90.64 | 92.93 | 95.13 | 97.10 | 99.12 |
| 32 | 86.58 | 89.07 | 91.33 | 93.64 | 95.86 | 97.87 | 99.91 |
| 33 | 87.20 | 89.72 | 92.00 | 94.35 | 96.59 | 98.62 | 100.68 |
| 34 | 87.82 | 90.37 | 92.68 | 95.04 | 97.31 | 99.36 | 101.44 |
| 35 | 88.43 | 91.01 | 93.34 | 95.73 | 98.03 | 100.09 | 102.20 |
| 36 | 89.04 | 91.65 | 94.00 | 96.42 | 98.74 | 100.83 | 102.96 |

By Wuhan University Children's Digital Health and Data Center (CDHDC)

# The Postnatal Growth Reference for Preterm Infants

WUHAN UNIVERSITY

## Weight(34w girls)

| Corrected age(months) | Centiles | | | | | | |
|---|---|---|---|---|---|---|---|
| | 3rd | 10rd | 25rd | 50rd | 75rd | 90rd | 97rd |
| 0 | 2.50 | 2.80 | 3.10 | 3.47 | 3.80 | 4.10 | 4.35 |
| 1 | 3.36 | 3.74 | 4.12 | 4.53 | 4.96 | 5.37 | 5.81 |
| 2 | 4.07 | 4.53 | 4.97 | 5.47 | 5.97 | 6.46 | 6.98 |
| 3 | 4.69 | 5.20 | 5.71 | 6.27 | 6.84 | 7.38 | 7.97 |
| 4 | 5.21 | 5.77 | 6.32 | 6.93 | 7.55 | 8.15 | 8.79 |
| 5 | 5.64 | 6.24 | 6.83 | 7.47 | 8.13 | 8.77 | 9.45 |
| 6 | 6.00 | 6.63 | 7.24 | 7.92 | 8.61 | 9.27 | 9.98 |
| 7 | 6.31 | 6.95 | 7.59 | 8.28 | 8.99 | 9.68 | 10.41 |
| 8 | 6.57 | 7.23 | 7.88 | 8.59 | 9.32 | 10.02 | 10.76 |
| 9 | 6.79 | 7.47 | 8.13 | 8.85 | 9.60 | 10.31 | 11.07 |
| 10 | 7.00 | 7.68 | 8.35 | 9.09 | 9.85 | 10.57 | 11.34 |
| 11 | 7.18 | 7.88 | 8.56 | 9.31 | 10.07 | 10.80 | 11.59 |
| 12 | 7.36 | 8.06 | 8.75 | 9.51 | 10.29 | 11.03 | 11.82 |
| 13 | 7.53 | 8.25 | 8.94 | 9.71 | 10.49 | 11.24 | 12.04 |
| 14 | 7.71 | 8.43 | 9.13 | 9.91 | 10.70 | 11.45 | 12.26 |
| 15 | 7.89 | 8.62 | 9.33 | 10.10 | 10.90 | 11.66 | 12.48 |
| 16 | 8.07 | 8.81 | 9.52 | 10.31 | 11.11 | 11.87 | 12.69 |
| 17 | 8.26 | 9.00 | 9.72 | 10.51 | 11.32 | 12.09 | 12.91 |
| 18 | 8.46 | 9.20 | 9.92 | 10.72 | 11.53 | 12.30 | 13.13 |
| 19 | 8.65 | 9.40 | 10.12 | 10.92 | 11.74 | 12.52 | 13.35 |
| 20 | 8.84 | 9.59 | 10.33 | 11.13 | 11.95 | 12.73 | 13.57 |
| 21 | 9.03 | 9.79 | 10.53 | 11.33 | 12.16 | 12.95 | 13.79 |
| 22 | 9.21 | 9.98 | 10.72 | 11.54 | 12.37 | 13.16 | 14.01 |
| 23 | 9.40 | 10.17 | 10.92 | 11.74 | 12.58 | 13.38 | 14.24 |
| 24 | 9.57 | 10.35 | 11.11 | 11.94 | 12.79 | 13.59 | 14.46 |
| 25 | 9.74 | 10.53 | 11.29 | 12.13 | 12.99 | 13.81 | 14.68 |
| 26 | 9.90 | 10.69 | 11.47 | 12.32 | 13.19 | 14.02 | 14.90 |
| 27 | 10.05 | 10.86 | 11.65 | 12.51 | 13.39 | 14.23 | 15.12 |
| 28 | 10.20 | 11.02 | 11.82 | 12.69 | 13.58 | 14.43 | 15.34 |
| 29 | 10.34 | 11.18 | 11.99 | 12.87 | 13.78 | 14.64 | 15.56 |
| 30 | 10.48 | 11.33 | 12.15 | 13.05 | 13.97 | 14.84 | 15.77 |
| 31 | 10.62 | 11.48 | 12.31 | 13.22 | 14.15 | 15.04 | 15.99 |
| 32 | 10.76 | 11.63 | 12.47 | 13.39 | 14.34 | 15.23 | 16.20 |
| 33 | 10.89 | 11.77 | 12.63 | 13.56 | 14.52 | 15.43 | 16.40 |
| 34 | 11.02 | 11.92 | 12.78 | 13.73 | 14.70 | 15.62 | 16.61 |
| 35 | 11.16 | 12.06 | 12.94 | 13.90 | 14.88 | 15.81 | 16.81 |
| 36 | 11.29 | 12.20 | 13.09 | 14.06 | 15.06 | 16.00 | 17.01 |

By Wuhan University Children’s Digital Health and Data Center (CDHDC)

# The Postnatal Growth Reference for Preterm Infants

## Head circumference(34w girls)

| Corrected age(months) | Centiles | | | | | | |
|---|---|---|---|---|---|---|---|
| | 3rd | 10rd | 25rd | 50rd | 75rd | 90rd | 97rd |
| 0 | 32.40 | 33.20 | 34.00 | 35.00 | 35.60 | 36.33 | 37.00 |
| 1 | 34.34 | 35.21 | 36.00 | 36.83 | 37.65 | 38.40 | 39.18 |
| 2 | 35.77 | 36.64 | 37.45 | 38.29 | 39.12 | 39.87 | 40.67 |
| 3 | 37.04 | 37.93 | 38.74 | 39.59 | 40.42 | 41.19 | 42.00 |
| 4 | 38.14 | 39.04 | 39.86 | 40.72 | 41.56 | 42.34 | 43.15 |
| 5 | 39.07 | 39.97 | 40.80 | 41.67 | 42.52 | 43.31 | 44.13 |
| 6 | 39.84 | 40.75 | 41.59 | 42.46 | 43.32 | 44.12 | 44.95 |
| 7 | 40.49 | 41.40 | 42.24 | 43.13 | 44.00 | 44.79 | 45.63 |
| 8 | 41.02 | 41.94 | 42.79 | 43.68 | 44.55 | 45.36 | 46.20 |
| 9 | 41.47 | 42.39 | 43.25 | 44.14 | 45.02 | 45.83 | 46.68 |
| 10 | 41.85 | 42.78 | 43.64 | 44.54 | 45.42 | 46.23 | 47.08 |
| 11 | 42.18 | 43.11 | 43.97 | 44.87 | 45.76 | 46.57 | 47.42 |
| 12 | 42.47 | 43.40 | 44.26 | 45.16 | 46.05 | 46.86 | 47.72 |
| 13 | 42.73 | 43.66 | 44.52 | 45.42 | 46.31 | 47.12 | 47.98 |
| 14 | 42.97 | 43.90 | 44.76 | 45.66 | 46.54 | 47.36 | 48.21 |
| 15 | 43.18 | 44.11 | 44.97 | 45.87 | 46.76 | 47.58 | 48.43 |
| 16 | 43.38 | 44.32 | 45.17 | 46.08 | 46.96 | 47.78 | 48.63 |
| 17 | 43.57 | 44.50 | 45.36 | 46.26 | 47.15 | 47.97 | 48.82 |
| 18 | 43.74 | 44.68 | 45.54 | 46.44 | 47.33 | 48.15 | 49.01 |
| 19 | 43.91 | 44.84 | 45.71 | 46.61 | 47.50 | 48.32 | 49.18 |
| 20 | 44.07 | 45.00 | 45.87 | 46.78 | 47.67 | 48.49 | 49.35 |
| 21 | 44.23 | 45.17 | 46.03 | 46.94 | 47.84 | 48.66 | 49.52 |
| 22 | 44.39 | 45.33 | 46.20 | 47.11 | 48.00 | 48.83 | 49.69 |
| 23 | 44.55 | 45.49 | 46.36 | 47.27 | 48.16 | 48.99 | 49.85 |
| 24 | 44.71 | 45.65 | 46.51 | 47.43 | 48.32 | 49.15 | 50.01 |
| 25 | 44.86 | 45.80 | 46.67 | 47.58 | 48.47 | 49.29 | 50.16 |
| 26 | 45.01 | 45.95 | 46.81 | 47.72 | 48.61 | 49.43 | 50.30 |
| 27 | 45.16 | 46.09 | 46.95 | 47.86 | 48.75 | 49.57 | 50.43 |
| 28 | 45.30 | 46.23 | 47.09 | 47.99 | 48.87 | 49.69 | 50.55 |
| 29 | 45.44 | 46.36 | 47.22 | 48.11 | 48.99 | 49.81 | 50.66 |
| 30 | 45.58 | 46.49 | 47.34 | 48.23 | 49.11 | 49.92 | 50.76 |
| 31 | 45.71 | 46.62 | 47.46 | 48.35 | 49.22 | 50.02 | 50.86 |
| 32 | 45.84 | 46.75 | 47.58 | 48.46 | 49.33 | 50.12 | 50.96 |
| 33 | 45.97 | 46.87 | 47.70 | 48.57 | 49.43 | 50.22 | 51.05 |
| 34 | 46.10 | 46.99 | 47.82 | 48.68 | 49.53 | 50.32 | 51.14 |
| 35 | 46.23 | 47.11 | 47.93 | 48.79 | 49.63 | 50.41 | 51.23 |
| 36 | 46.35 | 47.23 | 48.04 | 48.89 | 49.73 | 50.51 | 51.32 |

By Wuhan University Children’s Digital Health and Data Center (CDHDC)

# The Postnatal Growth Reference for Preterm Infants

WUHAN UNIVERSITY

## BMI(34w girls)

| Corrected age(months) | Centiles | | | | | | |
|---|---|---|---|---|---|---|---|
| | 3rd | 10rd | 25rd | 50rd | 75rd | 90rd | 97rd |
| 0 | 10.99 | 11.69 | 12.40 | 13.30 | 14.12 | 15.00 | 15.87 |
| 1 | 12.23 | 13.11 | 13.99 | 14.99 | 16.05 | 17.10 | 18.28 |
| 2 | 13.03 | 13.94 | 14.86 | 15.89 | 16.99 | 18.08 | 19.29 |
| 3 | 13.65 | 14.59 | 15.53 | 16.59 | 17.72 | 18.83 | 20.07 |
| 4 | 14.09 | 15.05 | 16.00 | 17.08 | 18.22 | 19.34 | 20.60 |
| 5 | 14.38 | 15.34 | 16.29 | 17.37 | 18.51 | 19.63 | 20.89 |
| 6 | 14.53 | 15.48 | 16.43 | 17.50 | 18.62 | 19.74 | 20.98 |
| 7 | 14.59 | 15.52 | 16.45 | 17.50 | 18.61 | 19.70 | 20.92 |
| 8 | 14.57 | 15.49 | 16.40 | 17.43 | 18.51 | 19.58 | 20.77 |
| 9 | 14.51 | 15.40 | 16.30 | 17.30 | 18.36 | 19.40 | 20.55 |
| 10 | 14.41 | 15.29 | 16.16 | 17.14 | 18.17 | 19.18 | 20.31 |
| 11 | 14.30 | 15.16 | 16.01 | 16.96 | 17.97 | 18.95 | 20.05 |
| 12 | 14.17 | 15.01 | 15.85 | 16.78 | 17.76 | 18.73 | 19.80 |
| 13 | 14.05 | 14.87 | 15.69 | 16.60 | 17.57 | 18.51 | 19.56 |
| 14 | 13.93 | 14.74 | 15.54 | 16.44 | 17.39 | 18.31 | 19.34 |
| 15 | 13.82 | 14.62 | 15.41 | 16.29 | 17.22 | 18.13 | 19.14 |
| 16 | 13.73 | 14.52 | 15.29 | 16.16 | 17.07 | 17.97 | 18.96 |
| 17 | 13.66 | 14.43 | 15.19 | 16.05 | 16.94 | 17.82 | 18.79 |
| 18 | 13.59 | 14.35 | 15.11 | 15.95 | 16.83 | 17.69 | 18.64 |
| 19 | 13.54 | 14.29 | 15.03 | 15.86 | 16.72 | 17.57 | 18.51 |
| 20 | 13.50 | 14.24 | 14.97 | 15.78 | 16.64 | 17.47 | 18.39 |
| 21 | 13.47 | 14.20 | 14.92 | 15.72 | 16.56 | 17.38 | 18.29 |
| 22 | 13.45 | 14.17 | 14.88 | 15.67 | 16.50 | 17.31 | 18.20 |
| 23 | 13.43 | 14.14 | 14.84 | 15.62 | 16.44 | 17.24 | 18.12 |
| 24 | 13.41 | 14.12 | 14.81 | 15.59 | 16.40 | 17.19 | 18.06 |
| 25 | 13.40 | 14.09 | 14.78 | 15.55 | 16.35 | 17.14 | 18.00 |
| 26 | 13.38 | 14.07 | 14.75 | 15.52 | 16.31 | 17.09 | 17.95 |
| 27 | 13.35 | 14.04 | 14.72 | 15.48 | 16.27 | 17.04 | 17.90 |
| 28 | 13.33 | 14.02 | 14.69 | 15.44 | 16.23 | 17.00 | 17.85 |
| 29 | 13.30 | 13.98 | 14.66 | 15.41 | 16.19 | 16.95 | 17.80 |
| 30 | 13.27 | 13.95 | 14.62 | 15.37 | 16.15 | 16.91 | 17.75 |
| 31 | 13.24 | 13.92 | 14.59 | 15.33 | 16.11 | 16.86 | 17.70 |
| 32 | 13.21 | 13.89 | 14.55 | 15.29 | 16.07 | 16.82 | 17.65 |
| 33 | 13.18 | 13.85 | 14.51 | 15.25 | 16.02 | 16.78 | 17.61 |
| 34 | 13.15 | 13.82 | 14.48 | 15.21 | 15.98 | 16.73 | 17.56 |
| 35 | 13.12 | 13.78 | 14.44 | 15.17 | 15.94 | 16.68 | 17.51 |
| 36 | 13.08 | 13.75 | 14.40 | 15.13 | 15.89 | 16.64 | 17.46 |

By Wuhan University Children’s Digital Health and Data Center (CDHDC)

# The Postnatal Growth Reference for Preterm Infants

WUHAN UNIVERSITY

## Length (33w girls)

| Corrected age(months) | Centiles | | | | | | |
|---|---|---|---|---|---|---|---|
| | 3rd | 10rd | 25rd | 50rd | 75rd | 90rd | 97rd |
| 0 | 46.06 | 47.70 | 49.30 | 50.70 | 52.00 | 53.67 | 54.86 |
| 1 | 50.11 | 51.70 | 53.17 | 54.72 | 56.23 | 57.59 | 58.97 |
| 2 | 53.28 | 54.87 | 56.36 | 57.92 | 59.44 | 60.81 | 62.21 |
| 3 | 56.15 | 57.74 | 59.22 | 60.78 | 62.31 | 63.68 | 65.09 |
| 4 | 58.63 | 60.22 | 61.70 | 63.27 | 64.79 | 66.17 | 67.58 |
| 5 | 60.75 | 62.33 | 63.82 | 65.39 | 66.91 | 68.30 | 69.71 |
| 6 | 62.55 | 64.14 | 65.64 | 67.21 | 68.75 | 70.15 | 71.57 |
| 7 | 64.11 | 65.72 | 67.23 | 68.82 | 70.38 | 71.79 | 73.23 |
| 8 | 65.49 | 67.12 | 68.65 | 70.27 | 71.85 | 73.28 | 74.74 |
| 9 | 66.74 | 68.40 | 69.96 | 71.60 | 73.20 | 74.66 | 76.14 |
| 10 | 67.90 | 69.59 | 71.18 | 72.85 | 74.48 | 75.96 | 77.47 |
| 11 | 68.99 | 70.71 | 72.33 | 74.03 | 75.69 | 77.20 | 78.74 |
| 12 | 70.03 | 71.79 | 73.43 | 75.16 | 76.86 | 78.39 | 79.96 |
| 13 | 71.04 | 72.83 | 74.50 | 76.27 | 77.99 | 79.55 | 81.15 |
| 14 | 72.02 | 73.84 | 75.54 | 77.34 | 79.10 | 80.69 | 82.32 |
| 15 | 72.97 | 74.83 | 76.57 | 78.40 | 80.19 | 81.81 | 83.47 |
| 16 | 73.90 | 75.80 | 77.57 | 79.44 | 81.27 | 82.92 | 84.61 |
| 17 | 74.82 | 76.75 | 78.56 | 80.47 | 82.33 | 84.02 | 85.74 |
| 18 | 75.70 | 77.67 | 79.52 | 81.47 | 83.38 | 85.10 | 86.86 |
| 19 | 76.54 | 78.57 | 80.46 | 82.46 | 84.40 | 86.17 | 87.97 |
| 20 | 77.35 | 79.43 | 81.37 | 83.41 | 85.41 | 87.21 | 89.06 |
| 21 | 78.13 | 80.26 | 82.25 | 84.35 | 86.39 | 88.24 | 90.13 |
| 22 | 78.89 | 81.07 | 83.11 | 85.26 | 87.35 | 89.25 | 91.18 |
| 23 | 79.61 | 81.85 | 83.94 | 86.15 | 88.29 | 90.24 | 92.22 |
| 24 | 80.31 | 82.61 | 84.75 | 87.01 | 89.21 | 91.20 | 93.23 |
| 25 | 80.98 | 83.34 | 85.54 | 87.85 | 90.11 | 92.14 | 94.22 |
| 26 | 81.64 | 84.06 | 86.31 | 88.68 | 90.99 | 93.08 | 95.20 |
| 27 | 82.28 | 84.76 | 87.07 | 89.50 | 91.86 | 94.00 | 96.17 |
| 28 | 82.92 | 85.46 | 87.82 | 90.31 | 92.72 | 94.90 | 97.13 |
| 29 | 83.56 | 86.15 | 88.57 | 91.11 | 93.57 | 95.80 | 98.07 |
| 30 | 84.20 | 86.85 | 89.32 | 91.90 | 94.42 | 96.69 | 99.01 |
| 31 | 84.84 | 87.54 | 90.06 | 92.70 | 95.26 | 97.58 | 99.93 |
| 32 | 85.50 | 88.25 | 90.81 | 93.50 | 96.11 | 98.46 | 100.86 |
| 33 | 86.15 | 88.96 | 91.56 | 94.30 | 96.95 | 99.35 | 101.78 |
| 34 | 86.82 | 89.67 | 92.32 | 95.10 | 97.80 | 100.23 | 102.70 |
| 35 | 87.48 | 90.38 | 93.07 | 95.90 | 98.64 | 101.11 | 103.62 |
| 36 | 88.15 | 91.09 | 93.83 | 96.69 | 99.48 | 101.99 | 104.54 |

By Wuhan University Children’s Digital Health and Data Center (CDHDC)

# The Postnatal Growth Reference for Preterm Infants

WUHAN UNIVERSITY

## Weight (33w girls)

| Corrected age(months) | Centiles | | | | | | |
|---|---|---|---|---|---|---|---|
| | 3rd | 10rd | 25rd | 50rd | 75rd | 90rd | 97rd |
| 0 | 2.41 | 2.76 | 3.10 | 3.40 | 3.77 | 4.09 | 4.30 |
| 1 | 3.33 | 3.73 | 4.12 | 4.55 | 4.98 | 5.40 | 5.87 |
| 2 | 4.07 | 4.54 | 5.00 | 5.49 | 6.00 | 6.50 | 7.03 |
| 3 | 4.71 | 5.24 | 5.75 | 6.30 | 6.87 | 7.42 | 8.02 |
| 4 | 5.25 | 5.82 | 6.36 | 6.96 | 7.57 | 8.16 | 8.81 |
| 5 | 5.69 | 6.28 | 6.86 | 7.49 | 8.13 | 8.75 | 9.43 |
| 6 | 6.04 | 6.66 | 7.26 | 7.91 | 8.58 | 9.22 | 9.92 |
| 7 | 6.34 | 6.98 | 7.59 | 8.26 | 8.95 | 9.60 | 10.32 |
| 8 | 6.59 | 7.25 | 7.87 | 8.56 | 9.26 | 9.93 | 10.66 |
| 9 | 6.81 | 7.48 | 8.11 | 8.81 | 9.52 | 10.21 | 10.95 |
| 10 | 7.00 | 7.68 | 8.33 | 9.03 | 9.76 | 10.45 | 11.21 |
| 11 | 7.18 | 7.86 | 8.52 | 9.24 | 9.97 | 10.68 | 11.44 |
| 12 | 7.33 | 8.03 | 8.70 | 9.43 | 10.17 | 10.88 | 11.66 |
| 13 | 7.48 | 8.19 | 8.87 | 9.60 | 10.36 | 11.09 | 11.88 |
| 14 | 7.62 | 8.34 | 9.03 | 9.78 | 10.55 | 11.29 | 12.09 |
| 15 | 7.76 | 8.49 | 9.19 | 9.96 | 10.74 | 11.49 | 12.31 |
| 16 | 7.90 | 8.65 | 9.36 | 10.14 | 10.94 | 11.71 | 12.54 |
| 17 | 8.05 | 8.81 | 9.54 | 10.34 | 11.15 | 11.93 | 12.78 |
| 18 | 8.19 | 8.97 | 9.72 | 10.53 | 11.36 | 12.16 | 13.03 |
| 19 | 8.33 | 9.13 | 9.90 | 10.73 | 11.58 | 12.40 | 13.29 |
| 20 | 8.47 | 9.29 | 10.07 | 10.93 | 11.80 | 12.64 | 13.55 |
| 21 | 8.61 | 9.45 | 10.25 | 11.13 | 12.02 | 12.88 | 13.82 |
| 22 | 8.75 | 9.61 | 10.43 | 11.32 | 12.24 | 13.12 | 14.08 |
| 23 | 8.89 | 9.76 | 10.60 | 11.52 | 12.45 | 13.35 | 14.33 |
| 24 | 9.02 | 9.91 | 10.77 | 11.71 | 12.67 | 13.59 | 14.59 |
| 25 | 9.15 | 10.06 | 10.94 | 11.90 | 12.88 | 13.82 | 14.84 |
| 26 | 9.28 | 10.21 | 11.11 | 12.09 | 13.09 | 14.05 | 15.10 |
| 27 | 9.40 | 10.36 | 11.27 | 12.28 | 13.30 | 14.29 | 15.36 |
| 28 | 9.53 | 10.50 | 11.44 | 12.47 | 13.52 | 14.53 | 15.63 |
| 29 | 9.65 | 10.65 | 11.61 | 12.66 | 13.74 | 14.77 | 15.90 |
| 30 | 9.78 | 10.80 | 11.79 | 12.86 | 13.96 | 15.02 | 16.18 |
| 31 | 9.91 | 10.96 | 11.96 | 13.07 | 14.19 | 15.28 | 16.46 |
| 32 | 10.04 | 11.11 | 12.15 | 13.27 | 14.43 | 15.54 | 16.75 |
| 33 | 10.18 | 11.27 | 12.33 | 13.48 | 14.67 | 15.81 | 17.05 |
| 34 | 10.31 | 11.43 | 12.52 | 13.70 | 14.91 | 16.08 | 17.35 |
| 35 | 10.45 | 11.60 | 12.70 | 13.91 | 15.16 | 16.35 | 17.66 |
| 36 | 10.58 | 11.76 | 12.89 | 14.13 | 15.40 | 16.63 | 17.96 |

By Wuhan University Children's Digital Health and Data Center (CDHDC)

# The Postnatal Growth Reference for Preterm Infants

## Head circumference (33w girls)

| Corrected age(months) | Centiles | | | | | | |
|---|---|---|---|---|---|---|---|
| | 3rd | 10rd | 25rd | 50rd | 75rd | 90rd | 97rd |
| 0 | 32.30 | 33.40 | 34.00 | 34.80 | 35.50 | 36.13 | 36.60 |
| 1 | 34.41 | 35.28 | 36.05 | 36.86 | 37.66 | 38.42 | 39.25 |
| 2 | 35.83 | 36.70 | 37.48 | 38.30 | 39.10 | 39.86 | 40.70 |
| 3 | 37.10 | 37.98 | 38.76 | 39.58 | 40.39 | 41.16 | 42.00 |
| 4 | 38.19 | 39.07 | 39.86 | 40.68 | 41.50 | 42.27 | 43.12 |
| 5 | 39.10 | 39.98 | 40.78 | 41.61 | 42.43 | 43.22 | 44.07 |
| 6 | 39.85 | 40.74 | 41.55 | 42.39 | 43.22 | 44.01 | 44.87 |
| 7 | 40.46 | 41.37 | 42.19 | 43.03 | 43.88 | 44.67 | 45.55 |
| 8 | 40.98 | 41.90 | 42.72 | 43.58 | 44.43 | 45.24 | 46.12 |
| 9 | 41.42 | 42.35 | 43.18 | 44.04 | 44.90 | 45.71 | 46.61 |
| 10 | 41.80 | 42.73 | 43.56 | 44.43 | 45.30 | 46.12 | 47.02 |
| 11 | 42.13 | 43.06 | 43.90 | 44.77 | 45.64 | 46.46 | 47.36 |
| 12 | 42.41 | 43.35 | 44.19 | 45.06 | 45.93 | 46.75 | 47.65 |
| 13 | 42.67 | 43.60 | 44.44 | 45.31 | 46.18 | 47.00 | 47.90 |
| 14 | 42.91 | 43.84 | 44.67 | 45.54 | 46.40 | 47.22 | 48.12 |
| 15 | 43.13 | 44.05 | 44.88 | 45.74 | 46.60 | 47.42 | 48.31 |
| 16 | 43.33 | 44.25 | 45.08 | 45.94 | 46.79 | 47.60 | 48.49 |
| 17 | 43.52 | 44.44 | 45.26 | 46.12 | 46.97 | 47.78 | 48.67 |
| 18 | 43.69 | 44.61 | 45.43 | 46.29 | 47.14 | 47.95 | 48.84 |
| 19 | 43.85 | 44.77 | 45.60 | 46.46 | 47.31 | 48.12 | 49.01 |
| 20 | 44.01 | 44.93 | 45.76 | 46.62 | 47.47 | 48.29 | 49.18 |
| 21 | 44.16 | 45.08 | 45.91 | 46.78 | 47.63 | 48.45 | 49.34 |
| 22 | 44.30 | 45.23 | 46.06 | 46.93 | 47.79 | 48.61 | 49.50 |
| 23 | 44.44 | 45.37 | 46.21 | 47.08 | 47.94 | 48.76 | 49.66 |
| 24 | 44.58 | 45.51 | 46.35 | 47.22 | 48.08 | 48.91 | 49.81 |
| 25 | 44.71 | 45.64 | 46.48 | 47.36 | 48.22 | 49.05 | 49.95 |
| 26 | 44.84 | 45.77 | 46.62 | 47.49 | 48.36 | 49.19 | 50.09 |
| 27 | 44.97 | 45.91 | 46.75 | 47.62 | 48.49 | 49.32 | 50.23 |
| 28 | 45.10 | 46.04 | 46.88 | 47.76 | 48.63 | 49.45 | 50.36 |
| 29 | 45.23 | 46.17 | 47.01 | 47.89 | 48.76 | 49.59 | 50.49 |
| 30 | 45.36 | 46.30 | 47.14 | 48.02 | 48.89 | 49.72 | 50.63 |
| 31 | 45.50 | 46.44 | 47.28 | 48.16 | 49.03 | 49.85 | 50.76 |
| 32 | 45.64 | 46.58 | 47.42 | 48.30 | 49.16 | 49.99 | 50.90 |
| 33 | 45.79 | 46.72 | 47.56 | 48.44 | 49.31 | 50.13 | 51.04 |
| 34 | 45.94 | 46.87 | 47.71 | 48.58 | 49.45 | 50.28 | 51.18 |
| 35 | 46.09 | 47.02 | 47.86 | 48.73 | 49.60 | 50.42 | 51.32 |
| 36 | 46.25 | 47.18 | 48.01 | 48.88 | 49.74 | 50.57 | 51.47 |

# The Postnatal Growth Reference for Preterm Infants

## BMI (33w girls)

| Corrected age(months) | Centiles | | | | | | |
|---|---|---|---|---|---|---|---|
| | 3rd | 10rd | 25rd | 50rd | 75rd | 90rd | 97rd |
| 0 | 10.63 | 11.56 | 12.35 | 13.24 | 14.14 | 14.97 | 15.56 |
| 1 | 12.17 | 13.16 | 14.10 | 15.14 | 16.23 | 17.33 | 18.58 |
| 2 | 12.93 | 13.94 | 14.92 | 16.00 | 17.12 | 18.25 | 19.54 |
| 3 | 13.53 | 14.57 | 15.57 | 16.67 | 17.82 | 18.96 | 20.27 |
| 4 | 13.96 | 15.01 | 16.02 | 17.12 | 18.28 | 19.44 | 20.76 |
| 5 | 14.23 | 15.28 | 16.29 | 17.39 | 18.54 | 19.68 | 20.99 |
| 6 | 14.38 | 15.42 | 16.41 | 17.49 | 18.63 | 19.75 | 21.04 |
| 7 | 14.44 | 15.45 | 16.42 | 17.48 | 18.59 | 19.69 | 20.95 |
| 8 | 14.42 | 15.41 | 16.36 | 17.40 | 18.48 | 19.55 | 20.77 |
| 9 | 14.36 | 15.33 | 16.25 | 17.26 | 18.31 | 19.35 | 20.54 |
| 10 | 14.26 | 15.20 | 16.11 | 17.09 | 18.12 | 19.13 | 20.29 |
| 11 | 14.14 | 15.06 | 15.94 | 16.91 | 17.91 | 18.90 | 20.03 |
| 12 | 14.01 | 14.91 | 15.78 | 16.72 | 17.70 | 18.67 | 19.77 |
| 13 | 13.87 | 14.76 | 15.61 | 16.53 | 17.49 | 18.44 | 19.52 |
| 14 | 13.75 | 14.62 | 15.45 | 16.36 | 17.30 | 18.23 | 19.29 |
| 15 | 13.63 | 14.49 | 15.31 | 16.20 | 17.13 | 18.05 | 19.09 |
| 16 | 13.52 | 14.37 | 15.18 | 16.06 | 16.98 | 17.89 | 18.92 |
| 17 | 13.43 | 14.27 | 15.07 | 15.95 | 16.86 | 17.75 | 18.77 |
| 18 | 13.35 | 14.19 | 14.98 | 15.85 | 16.75 | 17.64 | 18.65 |
| 19 | 13.28 | 14.11 | 14.90 | 15.76 | 16.66 | 17.54 | 18.55 |
| 20 | 13.22 | 14.05 | 14.83 | 15.69 | 16.58 | 17.46 | 18.46 |
| 21 | 13.16 | 13.99 | 14.77 | 15.62 | 16.51 | 17.38 | 18.38 |
| 22 | 13.11 | 13.93 | 14.71 | 15.56 | 16.44 | 17.31 | 18.30 |
| 23 | 13.06 | 13.88 | 14.65 | 15.50 | 16.38 | 17.25 | 18.23 |
| 24 | 13.02 | 13.83 | 14.60 | 15.44 | 16.32 | 17.18 | 18.16 |
| 25 | 12.98 | 13.79 | 14.55 | 15.39 | 16.26 | 17.12 | 18.09 |
| 26 | 12.94 | 13.74 | 14.51 | 15.34 | 16.20 | 17.05 | 18.02 |
| 27 | 12.91 | 13.71 | 14.46 | 15.29 | 16.15 | 17.00 | 17.97 |
| 28 | 12.88 | 13.67 | 14.43 | 15.25 | 16.11 | 16.95 | 17.91 |
| 29 | 12.86 | 13.65 | 14.40 | 15.22 | 16.07 | 16.91 | 17.87 |
| 30 | 12.84 | 13.63 | 14.38 | 15.20 | 16.05 | 16.88 | 17.84 |
| 31 | 12.84 | 13.62 | 14.37 | 15.18 | 16.03 | 16.86 | 17.81 |
| 32 | 12.84 | 13.62 | 14.37 | 15.18 | 16.02 | 16.85 | 17.79 |
| 33 | 12.85 | 13.63 | 14.37 | 15.18 | 16.02 | 16.84 | 17.79 |
| 34 | 12.86 | 13.64 | 14.38 | 15.19 | 16.02 | 16.85 | 17.79 |
| 35 | 12.88 | 13.66 | 14.40 | 15.20 | 16.03 | 16.85 | 17.79 |
| 36 | 12.90 | 13.68 | 14.41 | 15.21 | 16.04 | 16.86 | 17.80 |

By Wuhan University Children's Digital Health and Data Center (CDHDC)

# The Postnatal Growth Reference for Preterm Infants

WUHAN UNIVERSITY

## Length (32w girls)

| Corrected age(months) | Centiles | | | | | | |
|---|---|---|---|---|---|---|---|
| | 3rd | 10rd | 25rd | 50rd | 75rd | 90rd | 97rd |
| 0 | 45.68 | 47.25 | 48.70 | 50.50 | 52.00 | 53.00 | 54.63 |
| 1 | 49.75 | 51.36 | 52.87 | 54.46 | 56.00 | 57.37 | 58.73 |
| 2 | 52.80 | 54.43 | 55.97 | 57.59 | 59.15 | 60.54 | 61.93 |
| 3 | 55.61 | 57.25 | 58.81 | 60.45 | 62.03 | 63.44 | 64.85 |
| 4 | 58.09 | 59.74 | 61.31 | 62.97 | 64.57 | 66.00 | 67.43 |
| 5 | 60.21 | 61.88 | 63.47 | 65.14 | 66.76 | 68.21 | 69.65 |
| 6 | 62.03 | 63.72 | 65.32 | 67.01 | 68.65 | 70.12 | 71.58 |
| 7 | 63.61 | 65.32 | 66.94 | 68.65 | 70.31 | 71.79 | 73.27 |
| 8 | 65.03 | 66.76 | 68.39 | 70.12 | 71.80 | 73.30 | 74.80 |
| 9 | 66.33 | 68.08 | 69.74 | 71.49 | 73.19 | 74.71 | 76.23 |
| 10 | 67.57 | 69.34 | 71.02 | 72.80 | 74.52 | 76.07 | 77.61 |
| 11 | 68.74 | 70.54 | 72.25 | 74.06 | 75.81 | 77.38 | 78.95 |
| 12 | 69.85 | 71.69 | 73.43 | 75.27 | 77.06 | 78.66 | 80.25 |
| 13 | 70.90 | 72.77 | 74.55 | 76.43 | 78.25 | 79.88 | 81.50 |
| 14 | 71.90 | 73.81 | 75.62 | 77.54 | 79.40 | 81.06 | 82.72 |
| 15 | 72.87 | 74.82 | 76.66 | 78.62 | 80.51 | 82.21 | 83.90 |
| 16 | 73.80 | 75.79 | 77.68 | 79.67 | 81.60 | 83.33 | 85.05 |
| 17 | 74.71 | 76.74 | 78.66 | 80.69 | 82.66 | 84.42 | 86.18 |
| 18 | 75.61 | 77.67 | 79.62 | 81.69 | 83.69 | 85.48 | 87.26 |
| 19 | 76.47 | 78.57 | 80.55 | 82.65 | 84.68 | 86.49 | 88.31 |
| 20 | 77.31 | 79.44 | 81.45 | 83.57 | 85.63 | 87.47 | 89.31 |
| 21 | 78.13 | 80.28 | 82.32 | 84.46 | 86.55 | 88.41 | 90.27 |
| 22 | 78.93 | 81.10 | 83.15 | 85.32 | 87.43 | 89.31 | 91.18 |
| 23 | 79.72 | 81.90 | 83.97 | 86.15 | 88.27 | 90.17 | 92.05 |
| 24 | 80.48 | 82.68 | 84.76 | 86.95 | 89.08 | 90.99 | 92.89 |
| 25 | 81.23 | 83.43 | 85.52 | 87.73 | 89.87 | 91.78 | 93.69 |
| 26 | 81.96 | 84.17 | 86.27 | 88.48 | 90.63 | 92.55 | 94.47 |
| 27 | 82.67 | 84.89 | 87.00 | 89.23 | 91.39 | 93.32 | 95.24 |
| 28 | 83.36 | 85.60 | 87.72 | 89.96 | 92.14 | 94.08 | 96.02 |
| 29 | 84.05 | 86.30 | 88.44 | 90.70 | 92.89 | 94.85 | 96.80 |
| 30 | 84.72 | 87.00 | 89.16 | 91.44 | 93.65 | 95.63 | 97.60 |
| 31 | 85.39 | 87.69 | 89.87 | 92.18 | 94.42 | 96.42 | 98.42 |
| 32 | 86.05 | 88.38 | 90.59 | 92.93 | 95.20 | 97.22 | 99.24 |
| 33 | 86.70 | 89.07 | 91.31 | 93.68 | 95.98 | 98.03 | 100.08 |
| 34 | 87.35 | 89.76 | 92.03 | 94.43 | 96.77 | 98.85 | 100.93 |
| 35 | 87.99 | 90.44 | 92.75 | 95.19 | 97.56 | 99.67 | 101.78 |
| 36 | 88.63 | 91.12 | 93.47 | 95.95 | 98.35 | 100.50 | 102.65 |

By Wuhan University Children’s Digital Health and Data Center (CDHDC)

# The Postnatal Growth Reference for Preterm Infants

## Weight(32w girls)

| Corrected age(months) | Centiles | | | | | | |
|---|---|---|---|---|---|---|---|
| | 3rd | 10rd | 25rd | 50rd | 75rd | 90rd | 97rd |
| 0 | 2.44 | 2.65 | 3.07 | 3.45 | 3.78 | 4.00 | 4.43 |
| 1 | 3.27 | 3.67 | 4.07 | 4.51 | 4.96 | 5.39 | 5.85 |
| 2 | 3.98 | 4.44 | 4.91 | 5.42 | 5.94 | 6.44 | 6.98 |
| 3 | 4.61 | 5.13 | 5.65 | 6.21 | 6.80 | 7.35 | 7.95 |
| 4 | 5.15 | 5.71 | 6.27 | 6.88 | 7.50 | 8.10 | 8.73 |
| 5 | 5.60 | 6.19 | 6.77 | 7.41 | 8.06 | 8.69 | 9.35 |
| 6 | 5.98 | 6.58 | 7.18 | 7.84 | 8.51 | 9.15 | 9.84 |
| 7 | 6.29 | 6.91 | 7.52 | 8.19 | 8.88 | 9.53 | 10.23 |
| 8 | 6.57 | 7.20 | 7.81 | 8.50 | 9.19 | 9.85 | 10.56 |
| 9 | 6.81 | 7.45 | 8.08 | 8.77 | 9.48 | 10.15 | 10.86 |
| 10 | 7.04 | 7.69 | 8.32 | 9.02 | 9.74 | 10.42 | 11.14 |
| 11 | 7.25 | 7.91 | 8.55 | 9.26 | 9.99 | 10.67 | 11.41 |
| 12 | 7.44 | 8.11 | 8.76 | 9.48 | 10.22 | 10.91 | 11.66 |
| 13 | 7.62 | 8.29 | 8.95 | 9.68 | 10.43 | 11.13 | 11.89 |
| 14 | 7.79 | 8.47 | 9.14 | 9.87 | 10.63 | 11.34 | 12.10 |
| 15 | 7.95 | 8.64 | 9.31 | 10.06 | 10.82 | 11.54 | 12.30 |
| 16 | 8.11 | 8.81 | 9.49 | 10.24 | 11.01 | 11.73 | 12.50 |
| 17 | 8.27 | 8.97 | 9.66 | 10.42 | 11.19 | 11.93 | 12.71 |
| 18 | 8.43 | 9.14 | 9.83 | 10.60 | 11.38 | 12.12 | 12.91 |
| 19 | 8.58 | 9.30 | 10.01 | 10.78 | 11.58 | 12.33 | 13.12 |
| 20 | 8.73 | 9.47 | 10.18 | 10.97 | 11.77 | 12.53 | 13.34 |
| 21 | 8.88 | 9.63 | 10.35 | 11.15 | 11.97 | 12.74 | 13.57 |
| 22 | 9.03 | 9.78 | 10.52 | 11.34 | 12.17 | 12.96 | 13.79 |
| 23 | 9.17 | 9.94 | 10.69 | 11.52 | 12.36 | 13.16 | 14.02 |
| 24 | 9.30 | 10.08 | 10.85 | 11.69 | 12.56 | 13.37 | 14.24 |
| 25 | 9.43 | 10.23 | 11.01 | 11.87 | 12.75 | 13.58 | 14.46 |
| 26 | 9.56 | 10.37 | 11.17 | 12.04 | 12.94 | 13.78 | 14.68 |
| 27 | 9.69 | 10.52 | 11.33 | 12.22 | 13.13 | 13.99 | 14.91 |
| 28 | 9.82 | 10.66 | 11.49 | 12.39 | 13.32 | 14.20 | 15.13 |
| 29 | 9.95 | 10.81 | 11.65 | 12.57 | 13.52 | 14.42 | 15.37 |
| 30 | 10.08 | 10.95 | 11.81 | 12.76 | 13.73 | 14.64 | 15.62 |
| 31 | 10.20 | 11.10 | 11.98 | 12.95 | 13.94 | 14.87 | 15.87 |
| 32 | 10.34 | 11.25 | 12.15 | 13.14 | 14.15 | 15.11 | 16.13 |
| 33 | 10.47 | 11.40 | 12.32 | 13.33 | 14.37 | 15.34 | 16.39 |
| 34 | 10.60 | 11.56 | 12.49 | 13.53 | 14.58 | 15.58 | 16.64 |
| 35 | 10.74 | 11.71 | 12.67 | 13.72 | 14.80 | 15.82 | 16.90 |
| 36 | 10.87 | 11.86 | 12.84 | 13.91 | 15.01 | 16.05 | 17.16 |

By Wuhan University Children's Digital Health and Data Center (CDHDC)

# The Postnatal Growth Reference for Preterm Infants

WUHAN UNIVERSITY

## Head circumference(32w girls)

| Corrected age(months) | Centiles | | | | | | |
|---|---|---|---|---|---|---|---|
| | 3rd | 10rd | 25rd | 50rd | 75rd | 90rd | 97rd |
| 0 | 32.00 | 33.00 | 34.00 | 34.60 | 35.50 | 36.20 | 37.00 |
| 1 | 34.23 | 35.15 | 35.97 | 36.81 | 37.63 | 38.39 | 39.21 |
| 2 | 35.59 | 36.52 | 37.35 | 38.20 | 39.02 | 39.79 | 40.61 |
| 3 | 36.84 | 37.77 | 38.60 | 39.45 | 40.28 | 41.06 | 41.88 |
| 4 | 37.93 | 38.87 | 39.70 | 40.56 | 41.39 | 42.17 | 43.00 |
| 5 | 38.87 | 39.81 | 40.64 | 41.50 | 42.33 | 43.11 | 43.94 |
| 6 | 39.68 | 40.61 | 41.44 | 42.30 | 43.13 | 43.91 | 44.74 |
| 7 | 40.36 | 41.29 | 42.12 | 42.97 | 43.80 | 44.58 | 45.41 |
| 8 | 40.94 | 41.87 | 42.69 | 43.54 | 44.37 | 45.15 | 45.98 |
| 9 | 41.42 | 42.35 | 43.18 | 44.03 | 44.86 | 45.63 | 46.46 |
| 10 | 41.82 | 42.75 | 43.58 | 44.43 | 45.27 | 46.05 | 46.88 |
| 11 | 42.15 | 43.09 | 43.92 | 44.78 | 45.62 | 46.40 | 47.24 |
| 12 | 42.43 | 43.37 | 44.21 | 45.07 | 45.91 | 46.70 | 47.54 |
| 13 | 42.66 | 43.60 | 44.45 | 45.32 | 46.17 | 46.96 | 47.81 |
| 14 | 42.85 | 43.81 | 44.66 | 45.54 | 46.40 | 47.19 | 48.05 |
| 15 | 43.03 | 44.00 | 44.86 | 45.74 | 46.60 | 47.41 | 48.27 |
| 16 | 43.21 | 44.18 | 45.04 | 45.93 | 46.80 | 47.61 | 48.48 |
| 17 | 43.38 | 44.36 | 45.23 | 46.12 | 47.00 | 47.81 | 48.68 |
| 18 | 43.55 | 44.53 | 45.41 | 46.31 | 47.19 | 48.00 | 48.88 |
| 19 | 43.72 | 44.70 | 45.58 | 46.48 | 47.37 | 48.19 | 49.07 |
| 20 | 43.88 | 44.87 | 45.75 | 46.66 | 47.54 | 48.37 | 49.25 |
| 21 | 44.03 | 45.02 | 45.91 | 46.82 | 47.71 | 48.54 | 49.42 |
| 22 | 44.18 | 45.17 | 46.06 | 46.97 | 47.86 | 48.69 | 49.58 |
| 23 | 44.33 | 45.32 | 46.21 | 47.12 | 48.01 | 48.84 | 49.73 |
| 24 | 44.46 | 45.46 | 46.34 | 47.25 | 48.14 | 48.97 | 49.86 |
| 25 | 44.60 | 45.59 | 46.47 | 47.38 | 48.27 | 49.10 | 49.99 |
| 26 | 44.73 | 45.72 | 46.60 | 47.51 | 48.40 | 49.23 | 50.12 |
| 27 | 44.85 | 45.84 | 46.72 | 47.63 | 48.52 | 49.35 | 50.24 |
| 28 | 44.98 | 45.96 | 46.85 | 47.75 | 48.64 | 49.47 | 50.36 |
| 29 | 45.09 | 46.08 | 46.96 | 47.87 | 48.76 | 49.59 | 50.47 |
| 30 | 45.21 | 46.20 | 47.08 | 47.99 | 48.88 | 49.71 | 50.59 |
| 31 | 45.33 | 46.32 | 47.20 | 48.11 | 49.00 | 49.83 | 50.71 |
| 32 | 45.45 | 46.44 | 47.32 | 48.23 | 49.12 | 49.95 | 50.84 |
| 33 | 45.57 | 46.56 | 47.44 | 48.35 | 49.24 | 50.07 | 50.96 |
| 34 | 45.68 | 46.67 | 47.56 | 48.47 | 49.36 | 50.19 | 51.08 |
| 35 | 45.80 | 46.79 | 47.68 | 48.59 | 49.48 | 50.31 | 51.20 |
| 36 | 45.91 | 46.90 | 47.79 | 48.70 | 49.59 | 50.42 | 51.31 |

By Wuhan University Children's Digital Health and Data Center (CDHDC)

# The Postnatal Growth Reference for Preterm Infants

WUHAN UNIVERSITY

## BMI(32w girls)

| Corrected age(months) | Centiles | | | | | | |
|---|---|---|---|---|---|---|---|
| | 3rd | 10rd | 25rd | 50rd | 75rd | 90rd | 97rd |
| 0 | 11.00 | 11.50 | 12.32 | 13.34 | 14.41 | 15.46 | 16.25 |
| 1 | 12.19 | 13.17 | 14.15 | 15.25 | 16.42 | 17.56 | 18.84 |
| 2 | 12.95 | 13.94 | 14.92 | 16.03 | 17.19 | 18.33 | 19.59 |
| 3 | 13.59 | 14.57 | 15.55 | 16.65 | 17.80 | 18.92 | 20.17 |
| 4 | 14.06 | 15.04 | 16.00 | 17.08 | 18.21 | 19.32 | 20.54 |
| 5 | 14.37 | 15.33 | 16.27 | 17.33 | 18.43 | 19.51 | 20.70 |
| 6 | 14.55 | 15.48 | 16.40 | 17.43 | 18.50 | 19.55 | 20.70 |
| 7 | 14.62 | 15.53 | 16.43 | 17.42 | 18.46 | 19.48 | 20.59 |
| 8 | 14.62 | 15.51 | 16.38 | 17.35 | 18.36 | 19.34 | 20.42 |
| 9 | 14.57 | 15.44 | 16.29 | 17.23 | 18.21 | 19.17 | 20.22 |
| 10 | 14.49 | 15.34 | 16.16 | 17.09 | 18.05 | 18.98 | 20.00 |
| 11 | 14.39 | 15.21 | 16.03 | 16.93 | 17.87 | 18.78 | 19.78 |
| 12 | 14.27 | 15.08 | 15.88 | 16.76 | 17.68 | 18.57 | 19.55 |
| 13 | 14.15 | 14.95 | 15.72 | 16.59 | 17.49 | 18.36 | 19.32 |
| 14 | 14.03 | 14.81 | 15.57 | 16.42 | 17.30 | 18.16 | 19.09 |
| 15 | 13.91 | 14.67 | 15.43 | 16.26 | 17.12 | 17.96 | 18.88 |
| 16 | 13.80 | 14.55 | 15.29 | 16.11 | 16.96 | 17.78 | 18.68 |
| 17 | 13.69 | 14.43 | 15.16 | 15.97 | 16.81 | 17.62 | 18.51 |
| 18 | 13.59 | 14.33 | 15.05 | 15.85 | 16.68 | 17.49 | 18.37 |
| 19 | 13.51 | 14.24 | 14.96 | 15.75 | 16.58 | 17.38 | 18.25 |
| 20 | 13.44 | 14.17 | 14.88 | 15.67 | 16.50 | 17.29 | 18.17 |
| 21 | 13.38 | 14.11 | 14.82 | 15.61 | 16.43 | 17.22 | 18.10 |
| 22 | 13.33 | 14.06 | 14.77 | 15.56 | 16.37 | 17.17 | 18.04 |
| 23 | 13.28 | 14.01 | 14.72 | 15.50 | 16.32 | 17.11 | 17.98 |
| 24 | 13.24 | 13.96 | 14.67 | 15.45 | 16.27 | 17.06 | 17.92 |
| 25 | 13.20 | 13.92 | 14.63 | 15.41 | 16.22 | 17.01 | 17.87 |
| 26 | 13.17 | 13.89 | 14.59 | 15.37 | 16.18 | 16.96 | 17.82 |
| 27 | 13.14 | 13.86 | 14.56 | 15.33 | 16.14 | 16.92 | 17.77 |
| 28 | 13.12 | 13.83 | 14.53 | 15.30 | 16.10 | 16.88 | 17.73 |
| 29 | 13.11 | 13.82 | 14.51 | 15.28 | 16.08 | 16.85 | 17.70 |
| 30 | 13.10 | 13.80 | 14.50 | 15.26 | 16.06 | 16.83 | 17.68 |
| 31 | 13.09 | 13.80 | 14.49 | 15.25 | 16.05 | 16.82 | 17.66 |
| 32 | 13.09 | 13.80 | 14.49 | 15.25 | 16.05 | 16.82 | 17.66 |
| 33 | 13.10 | 13.80 | 14.49 | 15.26 | 16.05 | 16.82 | 17.66 |
| 34 | 13.11 | 13.81 | 14.50 | 15.27 | 16.06 | 16.83 | 17.67 |
| 35 | 13.12 | 13.82 | 14.51 | 15.28 | 16.07 | 16.84 | 17.68 |
| 36 | 13.13 | 13.83 | 14.52 | 15.29 | 16.08 | 16.85 | 17.69 |

# The Postnatal Growth Reference for Preterm Infants

WUHAN UNIVERSITY

## Length(31w girls)

| Corrected age(months) | Centiles | | | | | | |
|---|---|---|---|---|---|---|---|
| | 3rd | 10rd | 25rd | 50rd | 75rd | 90rd | 97rd |
| 0 | 45.38 | 47.02 | 48.40 | 50.00 | 51.10 | 53.00 | 54.50 |
| 1 | 49.36 | 50.98 | 52.53 | 54.15 | 55.69 | 57.01 | 58.26 |
| 2 | 52.33 | 53.98 | 55.57 | 57.23 | 58.81 | 60.17 | 61.46 |
| 3 | 55.10 | 56.77 | 58.38 | 60.08 | 61.69 | 63.08 | 64.39 |
| 4 | 57.60 | 59.28 | 60.91 | 62.63 | 64.26 | 65.67 | 67.00 |
| 5 | 59.81 | 61.51 | 63.14 | 64.87 | 66.51 | 67.93 | 69.28 |
| 6 | 61.77 | 63.46 | 65.10 | 66.84 | 68.49 | 69.92 | 71.28 |
| 7 | 63.49 | 65.20 | 66.84 | 68.58 | 70.25 | 71.69 | 73.05 |
| 8 | 65.04 | 66.74 | 68.39 | 70.15 | 71.82 | 73.27 | 74.65 |
| 9 | 66.42 | 68.14 | 69.80 | 71.56 | 73.25 | 74.71 | 76.10 |
| 10 | 67.70 | 69.42 | 71.10 | 72.88 | 74.58 | 76.05 | 77.45 |
| 11 | 68.88 | 70.63 | 72.32 | 74.11 | 75.83 | 77.32 | 78.73 |
| 12 | 70.00 | 71.77 | 73.48 | 75.29 | 77.03 | 78.53 | 79.97 |
| 13 | 71.08 | 72.86 | 74.59 | 76.43 | 78.19 | 79.71 | 81.16 |
| 14 | 72.11 | 73.92 | 75.67 | 77.53 | 79.31 | 80.85 | 82.32 |
| 15 | 73.10 | 74.93 | 76.70 | 78.59 | 80.40 | 81.96 | 83.45 |
| 16 | 74.05 | 75.91 | 77.71 | 79.62 | 81.46 | 83.04 | 84.56 |
| 17 | 74.95 | 76.84 | 78.67 | 80.62 | 82.48 | 84.09 | 85.63 |
| 18 | 75.82 | 77.75 | 79.61 | 81.59 | 83.48 | 85.12 | 86.68 |
| 19 | 76.67 | 78.63 | 80.53 | 82.54 | 84.46 | 86.13 | 87.72 |
| 20 | 77.50 | 79.50 | 81.43 | 83.47 | 85.43 | 87.13 | 88.74 |
| 21 | 78.31 | 80.34 | 82.30 | 84.39 | 86.38 | 88.11 | 89.75 |
| 22 | 79.09 | 81.15 | 83.16 | 85.28 | 87.31 | 89.06 | 90.73 |
| 23 | 79.84 | 81.94 | 83.98 | 86.14 | 88.20 | 89.99 | 91.69 |
| 24 | 80.57 | 82.71 | 84.78 | 86.98 | 89.07 | 90.89 | 92.61 |
| 25 | 81.28 | 83.46 | 85.56 | 87.79 | 89.92 | 91.76 | 93.51 |
| 26 | 81.97 | 84.18 | 86.32 | 88.58 | 90.74 | 92.61 | 94.39 |
| 27 | 82.65 | 84.89 | 87.06 | 89.36 | 91.55 | 93.44 | 95.24 |
| 28 | 83.32 | 85.59 | 87.79 | 90.12 | 92.34 | 94.25 | 96.08 |
| 29 | 83.97 | 86.28 | 88.50 | 90.86 | 93.11 | 95.05 | 96.89 |
| 30 | 84.62 | 86.95 | 89.20 | 91.59 | 93.86 | 95.82 | 97.69 |
| 31 | 85.25 | 87.61 | 89.89 | 92.30 | 94.59 | 96.58 | 98.46 |
| 32 | 85.89 | 88.27 | 90.57 | 93.00 | 95.31 | 97.31 | 99.21 |
| 33 | 86.53 | 88.93 | 91.24 | 93.69 | 96.02 | 98.03 | 99.95 |
| 34 | 87.18 | 89.59 | 91.91 | 94.37 | 96.72 | 98.74 | 100.67 |
| 35 | 87.83 | 90.25 | 92.58 | 95.06 | 97.41 | 99.45 | 101.39 |
| 36 | 88.48 | 90.91 | 93.26 | 95.74 | 98.11 | 100.16 | 102.10 |

# The Postnatal Growth Reference for Preterm Infants

WUHAN UNIVERSITY

## Weight(31w girls)

| Corrected age(months) | Centiles | | | | | | |
|---|---|---|---|---|---|---|---|
| | 3rd | 10rd | 25rd | 50rd | 75rd | 90rd | 97rd |
| 0 | 2.34 | 2.62 | 2.90 | 3.30 | 3.60 | 4.02 | 4.39 |
| 1 | 3.15 | 3.54 | 3.94 | 4.40 | 4.85 | 5.27 | 5.68 |
| 2 | 3.83 | 4.28 | 4.74 | 5.26 | 5.79 | 6.27 | 6.74 |
| 3 | 4.46 | 4.96 | 5.47 | 6.04 | 6.62 | 7.14 | 7.67 |
| 4 | 5.01 | 5.55 | 6.09 | 6.71 | 7.32 | 7.89 | 8.44 |
| 5 | 5.49 | 6.05 | 6.62 | 7.27 | 7.91 | 8.50 | 9.09 |
| 6 | 5.90 | 6.48 | 7.07 | 7.74 | 8.41 | 9.01 | 9.62 |
| 7 | 6.26 | 6.85 | 7.46 | 8.13 | 8.82 | 9.44 | 10.06 |
| 8 | 6.56 | 7.16 | 7.78 | 8.47 | 9.17 | 9.80 | 10.43 |
| 9 | 6.82 | 7.43 | 8.06 | 8.76 | 9.47 | 10.11 | 10.75 |
| 10 | 7.04 | 7.66 | 8.30 | 9.01 | 9.73 | 10.38 | 11.03 |
| 11 | 7.24 | 7.87 | 8.52 | 9.24 | 9.97 | 10.64 | 11.29 |
| 12 | 7.42 | 8.06 | 8.72 | 9.46 | 10.20 | 10.87 | 11.54 |
| 13 | 7.58 | 8.24 | 8.90 | 9.65 | 10.41 | 11.09 | 11.77 |
| 14 | 7.74 | 8.40 | 9.08 | 9.84 | 10.61 | 11.30 | 12.00 |
| 15 | 7.89 | 8.56 | 9.25 | 10.02 | 10.80 | 11.51 | 12.21 |
| 16 | 8.03 | 8.72 | 9.42 | 10.20 | 10.99 | 11.71 | 12.42 |
| 17 | 8.18 | 8.87 | 9.58 | 10.38 | 11.18 | 11.91 | 12.63 |
| 18 | 8.32 | 9.03 | 9.75 | 10.55 | 11.37 | 12.10 | 12.84 |
| 19 | 8.47 | 9.19 | 9.92 | 10.74 | 11.56 | 12.31 | 13.05 |
| 20 | 8.62 | 9.35 | 10.09 | 10.92 | 11.76 | 12.52 | 13.27 |
| 21 | 8.78 | 9.52 | 10.27 | 11.11 | 11.96 | 12.73 | 13.49 |
| 22 | 8.94 | 9.68 | 10.44 | 11.29 | 12.15 | 12.93 | 13.71 |
| 23 | 9.09 | 9.85 | 10.62 | 11.48 | 12.35 | 13.14 | 13.92 |
| 24 | 9.24 | 10.01 | 10.79 | 11.66 | 12.54 | 13.34 | 14.13 |
| 25 | 9.39 | 10.16 | 10.95 | 11.84 | 12.73 | 13.54 | 14.35 |
| 26 | 9.53 | 10.31 | 11.12 | 12.02 | 12.92 | 13.74 | 14.56 |
| 27 | 9.67 | 10.46 | 11.28 | 12.19 | 13.11 | 13.94 | 14.77 |
| 28 | 9.80 | 10.61 | 11.44 | 12.36 | 13.30 | 14.14 | 14.98 |
| 29 | 9.93 | 10.75 | 11.59 | 12.53 | 13.48 | 14.34 | 15.19 |
| 30 | 10.05 | 10.89 | 11.74 | 12.70 | 13.66 | 14.54 | 15.40 |
| 31 | 10.18 | 11.03 | 11.90 | 12.87 | 13.85 | 14.74 | 15.63 |
| 32 | 10.30 | 11.17 | 12.06 | 13.05 | 14.05 | 14.96 | 15.86 |
| 33 | 10.42 | 11.31 | 12.22 | 13.23 | 14.26 | 15.18 | 16.11 |
| 34 | 10.55 | 11.46 | 12.38 | 13.42 | 14.47 | 15.42 | 16.36 |
| 35 | 10.67 | 11.60 | 12.55 | 13.62 | 14.69 | 15.66 | 16.63 |
| 36 | 10.79 | 11.74 | 12.72 | 13.81 | 14.92 | 15.91 | 16.91 |

By Wuhan University Children's Digital Health and Data Center (CDHDC)

# The Postnatal Growth Reference for Preterm Infants

WUHAN UNIVERSITY

## Head circumference(31w girls)

| Corrected age(months) | Centiles | | | | | | |
|---|---|---|---|---|---|---|---|
| | 3rd | 10rd | 25rd | 50rd | 75rd | 90rd | 97rd |
| 0 | 32.00 | 32.50 | 33.53 | 34.50 | 35.50 | 36.50 | 37.17 |
| 1 | 34.08 | 34.89 | 35.69 | 36.57 | 37.43 | 38.19 | 38.92 |
| 2 | 35.45 | 36.26 | 37.07 | 37.95 | 38.81 | 39.58 | 40.32 |
| 3 | 36.72 | 37.53 | 38.34 | 39.23 | 40.10 | 40.86 | 41.61 |
| 4 | 37.84 | 38.66 | 39.48 | 40.37 | 41.24 | 42.01 | 42.76 |
| 5 | 38.81 | 39.63 | 40.45 | 41.35 | 42.23 | 43.01 | 43.77 |
| 6 | 39.63 | 40.46 | 41.29 | 42.19 | 43.08 | 43.86 | 44.63 |
| 7 | 40.32 | 41.16 | 41.99 | 42.91 | 43.80 | 44.60 | 45.37 |
| 8 | 40.91 | 41.76 | 42.60 | 43.52 | 44.42 | 45.22 | 46.00 |
| 9 | 41.42 | 42.28 | 43.13 | 44.05 | 44.97 | 45.77 | 46.56 |
| 10 | 41.87 | 42.73 | 43.59 | 44.52 | 45.44 | 46.26 | 47.05 |
| 11 | 42.27 | 43.14 | 44.00 | 44.95 | 45.88 | 46.70 | 47.50 |
| 12 | 42.63 | 43.51 | 44.38 | 45.33 | 46.27 | 47.10 | 47.91 |
| 13 | 42.95 | 43.84 | 44.72 | 45.68 | 46.62 | 47.46 | 48.28 |
| 14 | 43.24 | 44.13 | 45.02 | 45.99 | 46.94 | 47.78 | 48.60 |
| 15 | 43.49 | 44.39 | 45.28 | 46.25 | 47.21 | 48.06 | 48.88 |
| 16 | 43.71 | 44.61 | 45.50 | 46.48 | 47.44 | 48.29 | 49.12 |
| 17 | 43.91 | 44.81 | 45.70 | 46.68 | 47.64 | 48.49 | 49.32 |
| 18 | 44.10 | 44.99 | 45.88 | 46.86 | 47.82 | 48.67 | 49.50 |
| 19 | 44.27 | 45.17 | 46.06 | 47.03 | 47.99 | 48.83 | 49.66 |
| 20 | 44.44 | 45.33 | 46.22 | 47.19 | 48.14 | 48.99 | 49.81 |
| 21 | 44.61 | 45.49 | 46.38 | 47.34 | 48.29 | 49.13 | 49.95 |
| 22 | 44.77 | 45.65 | 46.53 | 47.49 | 48.44 | 49.27 | 50.09 |
| 23 | 44.92 | 45.80 | 46.68 | 47.63 | 48.58 | 49.41 | 50.23 |
| 24 | 45.07 | 45.95 | 46.82 | 47.78 | 48.72 | 49.55 | 50.37 |
| 25 | 45.22 | 46.10 | 46.97 | 47.92 | 48.86 | 49.70 | 50.51 |
| 26 | 45.37 | 46.24 | 47.12 | 48.07 | 49.01 | 49.84 | 50.65 |
| 27 | 45.51 | 46.39 | 47.26 | 48.21 | 49.15 | 49.99 | 50.80 |
| 28 | 45.65 | 46.53 | 47.40 | 48.36 | 49.30 | 50.14 | 50.95 |
| 29 | 45.79 | 46.67 | 47.55 | 48.51 | 49.45 | 50.29 | 51.11 |
| 30 | 45.93 | 46.81 | 47.70 | 48.66 | 49.61 | 50.45 | 51.27 |
| 31 | 46.07 | 46.96 | 47.84 | 48.82 | 49.77 | 50.62 | 51.44 |
| 32 | 46.21 | 47.10 | 48.00 | 48.97 | 49.93 | 50.78 | 51.61 |
| 33 | 46.35 | 47.25 | 48.15 | 49.13 | 50.10 | 50.95 | 51.78 |
| 34 | 46.50 | 47.40 | 48.31 | 49.29 | 50.26 | 51.12 | 51.96 |
| 35 | 46.65 | 47.56 | 48.47 | 49.46 | 50.43 | 51.30 | 52.14 |
| 36 | 46.80 | 47.72 | 48.63 | 49.63 | 50.61 | 51.48 | 52.32 |

By Wuhan University Children's Digital Health and Data Center (CDHDC)

# The Postnatal Growth Reference for Preterm Infants

## BMI(31w girls)

| Corrected age(months) | Centiles | | | | | | |
|---|---|---|---|---|---|---|---|
| | 3rd | 10rd | 25rd | 50rd | 75rd | 90rd | 97rd |
| 0 | 10.57 | 11.41 | 12.15 | 13.02 | 14.11 | 14.92 | 15.92 |
| 1 | 12.09 | 12.99 | 13.94 | 15.03 | 16.17 | 17.24 | 18.34 |
| 2 | 12.77 | 13.68 | 14.65 | 15.76 | 16.91 | 18.00 | 19.12 |
| 3 | 13.34 | 14.27 | 15.24 | 16.36 | 17.53 | 18.62 | 19.75 |
| 4 | 13.78 | 14.72 | 15.69 | 16.81 | 17.98 | 19.07 | 20.19 |
| 5 | 14.10 | 15.02 | 15.99 | 17.10 | 18.26 | 19.34 | 20.45 |
| 6 | 14.29 | 15.21 | 16.16 | 17.26 | 18.40 | 19.46 | 20.55 |
| 7 | 14.40 | 15.30 | 16.23 | 17.31 | 18.42 | 19.46 | 20.53 |
| 8 | 14.42 | 15.30 | 16.22 | 17.27 | 18.36 | 19.38 | 20.42 |
| 9 | 14.39 | 15.25 | 16.15 | 17.18 | 18.25 | 19.25 | 20.27 |
| 10 | 14.32 | 15.17 | 16.05 | 17.06 | 18.11 | 19.08 | 20.08 |
| 11 | 14.22 | 15.06 | 15.93 | 16.92 | 17.95 | 18.91 | 19.89 |
| 12 | 14.12 | 14.94 | 15.80 | 16.78 | 17.79 | 18.74 | 19.70 |
| 13 | 14.01 | 14.82 | 15.66 | 16.63 | 17.63 | 18.57 | 19.52 |
| 14 | 13.90 | 14.70 | 15.54 | 16.49 | 17.48 | 18.40 | 19.34 |
| 15 | 13.80 | 14.59 | 15.42 | 16.36 | 17.34 | 18.25 | 19.18 |
| 16 | 13.71 | 14.49 | 15.31 | 16.24 | 17.21 | 18.11 | 19.03 |
| 17 | 13.62 | 14.40 | 15.21 | 16.14 | 17.09 | 17.98 | 18.89 |
| 18 | 13.56 | 14.33 | 15.13 | 16.04 | 16.99 | 17.87 | 18.77 |
| 19 | 13.50 | 14.26 | 15.06 | 15.96 | 16.90 | 17.78 | 18.67 |
| 20 | 13.45 | 14.21 | 15.00 | 15.89 | 16.82 | 17.69 | 18.57 |
| 21 | 13.41 | 14.16 | 14.94 | 15.83 | 16.75 | 17.61 | 18.48 |
| 22 | 13.37 | 14.11 | 14.88 | 15.76 | 16.68 | 17.52 | 18.39 |
| 23 | 13.33 | 14.07 | 14.83 | 15.70 | 16.60 | 17.44 | 18.30 |
| 24 | 13.29 | 14.02 | 14.77 | 15.64 | 16.53 | 17.36 | 18.21 |
| 25 | 13.25 | 13.97 | 14.72 | 15.57 | 16.46 | 17.28 | 18.12 |
| 26 | 13.21 | 13.92 | 14.66 | 15.51 | 16.38 | 17.20 | 18.03 |
| 27 | 13.17 | 13.88 | 14.61 | 15.45 | 16.31 | 17.11 | 17.93 |
| 28 | 13.14 | 13.84 | 14.56 | 15.38 | 16.23 | 17.02 | 17.83 |
| 29 | 13.11 | 13.80 | 14.51 | 15.32 | 16.16 | 16.94 | 17.73 |
| 30 | 13.09 | 13.77 | 14.47 | 15.27 | 16.10 | 16.86 | 17.65 |
| 31 | 13.09 | 13.75 | 14.44 | 15.23 | 16.04 | 16.80 | 17.57 |
| 32 | 13.09 | 13.74 | 14.43 | 15.20 | 16.00 | 16.75 | 17.50 |
| 33 | 13.09 | 13.74 | 14.41 | 15.18 | 15.97 | 16.70 | 17.45 |
| 34 | 13.10 | 13.74 | 14.41 | 15.16 | 15.94 | 16.66 | 17.39 |
| 35 | 13.12 | 13.75 | 14.40 | 15.15 | 15.91 | 16.62 | 17.34 |
| 36 | 13.13 | 13.75 | 14.40 | 15.13 | 15.89 | 16.58 | 17.30 |

By Wuhan University Children’s Digital Health and Data Center (CDHDC)

# The Postnatal Growth Reference for Preterm Infants

WUHAN UNIVERSITY

## Length (30w girls)

| Corrected age(months) | Centiles | | | | | | |
|---|---|---|---|---|---|---|---|
| | 3rd | 10rd | 25rd | 50rd | 75rd | 90rd | 97rd |
| 0 | 44.52 | 46.84 | 48.80 | 49.80 | 51.85 | 52.40 | 54.24 |
| 1 | 48.98 | 50.53 | 52.07 | 53.74 | 55.38 | 56.82 | 58.22 |
| 2 | 52.05 | 53.64 | 55.22 | 56.94 | 58.62 | 60.11 | 61.55 |
| 3 | 54.88 | 56.50 | 58.12 | 59.88 | 61.60 | 63.12 | 64.60 |
| 4 | 57.40 | 59.05 | 60.69 | 62.48 | 64.23 | 65.78 | 67.28 |
| 5 | 59.61 | 61.28 | 62.93 | 64.74 | 66.51 | 68.08 | 69.60 |
| 6 | 61.55 | 63.22 | 64.88 | 66.70 | 68.48 | 70.06 | 71.59 |
| 7 | 63.25 | 64.92 | 66.59 | 68.41 | 70.20 | 71.78 | 73.31 |
| 8 | 64.77 | 66.44 | 68.12 | 69.94 | 71.73 | 73.32 | 74.86 |
| 9 | 66.14 | 67.83 | 69.50 | 71.34 | 73.14 | 74.73 | 76.28 |
| 10 | 67.41 | 69.11 | 70.79 | 72.64 | 74.45 | 76.05 | 77.61 |
| 11 | 68.58 | 70.29 | 71.99 | 73.85 | 75.68 | 77.30 | 78.87 |
| 12 | 69.66 | 71.39 | 73.11 | 75.00 | 76.85 | 78.49 | 80.08 |
| 13 | 70.69 | 72.45 | 74.20 | 76.11 | 77.99 | 79.65 | 81.27 |
| 14 | 71.68 | 73.47 | 75.25 | 77.20 | 79.11 | 80.81 | 82.46 |
| 15 | 72.66 | 74.49 | 76.30 | 78.29 | 80.24 | 81.96 | 83.64 |
| 16 | 73.63 | 75.49 | 77.34 | 79.36 | 81.35 | 83.11 | 84.82 |
| 17 | 74.59 | 76.48 | 78.37 | 80.43 | 82.45 | 84.25 | 85.99 |
| 18 | 75.53 | 77.46 | 79.38 | 81.47 | 83.53 | 85.35 | 87.12 |
| 19 | 76.46 | 78.42 | 80.36 | 82.48 | 84.57 | 86.41 | 88.21 |
| 20 | 77.37 | 79.35 | 81.31 | 83.46 | 85.57 | 87.44 | 89.26 |
| 21 | 78.25 | 80.25 | 82.24 | 84.42 | 86.55 | 88.44 | 90.28 |
| 22 | 79.10 | 81.13 | 83.14 | 85.34 | 87.50 | 89.41 | 91.27 |
| 23 | 79.92 | 81.97 | 84.00 | 86.23 | 88.41 | 90.35 | 92.23 |
| 24 | 80.70 | 82.77 | 84.83 | 87.08 | 89.29 | 91.25 | 93.15 |
| 25 | 81.46 | 83.55 | 85.63 | 87.90 | 90.14 | 92.12 | 94.04 |
| 26 | 82.19 | 84.30 | 86.41 | 88.70 | 90.96 | 92.95 | 94.90 |
| 27 | 82.91 | 85.04 | 87.16 | 89.47 | 91.74 | 93.75 | 95.71 |
| 28 | 83.63 | 85.76 | 87.89 | 90.21 | 92.49 | 94.52 | 96.48 |
| 29 | 84.33 | 86.47 | 88.60 | 90.93 | 93.21 | 95.24 | 97.20 |
| 30 | 85.03 | 87.17 | 89.29 | 91.61 | 93.89 | 95.91 | 97.88 |
| 31 | 85.73 | 87.85 | 89.97 | 92.28 | 94.55 | 96.56 | 98.52 |
| 32 | 86.42 | 88.53 | 90.63 | 92.92 | 95.18 | 97.18 | 99.13 |
| 33 | 87.11 | 89.20 | 91.28 | 93.56 | 95.80 | 97.79 | 99.72 |
| 34 | 87.79 | 89.87 | 91.93 | 94.19 | 96.41 | 98.38 | 100.30 |
| 35 | 88.49 | 90.54 | 92.58 | 94.82 | 97.03 | 98.98 | 100.88 |
| 36 | 89.18 | 91.21 | 93.24 | 95.45 | 97.64 | 99.57 | 101.46 |

By Wuhan University Children’s Digital Health and Data Center (CDHDC)

# The Postnatal Growth Reference for Preterm Infants

WUHAN UNIVERSITY

## Weight(30w girls)

| Corrected age(months) | Centiles | | | | | | |
|---|---|---|---|---|---|---|---|
| | 3rd | 10rd | 25rd | 50rd | 75rd | 90rd | 97rd |
| 0 | 2.35 | 2.56 | 3.00 | 3.20 | 3.50 | 3.90 | 4.60 |
| 1 | 3.10 | 3.47 | 3.85 | 4.31 | 4.80 | 5.28 | 5.81 |
| 2 | 3.78 | 4.21 | 4.67 | 5.21 | 5.79 | 6.36 | 6.98 |
| 3 | 4.39 | 4.88 | 5.40 | 6.01 | 6.66 | 7.30 | 8.00 |
| 4 | 4.91 | 5.45 | 6.01 | 6.67 | 7.38 | 8.08 | 8.83 |
| 5 | 5.36 | 5.93 | 6.53 | 7.22 | 7.97 | 8.71 | 9.50 |
| 6 | 5.74 | 6.33 | 6.95 | 7.68 | 8.45 | 9.21 | 10.04 |
| 7 | 6.07 | 6.67 | 7.31 | 8.05 | 8.85 | 9.62 | 10.46 |
| 8 | 6.35 | 6.97 | 7.62 | 8.37 | 9.18 | 9.96 | 10.81 |
| 9 | 6.60 | 7.23 | 7.89 | 8.65 | 9.46 | 10.25 | 11.11 |
| 10 | 6.82 | 7.46 | 8.13 | 8.90 | 9.72 | 10.51 | 11.37 |
| 11 | 7.02 | 7.67 | 8.34 | 9.12 | 9.94 | 10.75 | 11.62 |
| 12 | 7.20 | 7.85 | 8.53 | 9.32 | 10.16 | 10.97 | 11.84 |
| 13 | 7.36 | 8.02 | 8.71 | 9.51 | 10.35 | 11.18 | 12.06 |
| 14 | 7.52 | 8.19 | 8.89 | 9.69 | 10.55 | 11.38 | 12.27 |
| 15 | 7.67 | 8.35 | 9.05 | 9.87 | 10.73 | 11.57 | 12.47 |
| 16 | 7.83 | 8.51 | 9.22 | 10.04 | 10.91 | 11.76 | 12.66 |
| 17 | 7.98 | 8.67 | 9.39 | 10.22 | 11.09 | 11.94 | 12.85 |
| 18 | 8.14 | 8.84 | 9.56 | 10.39 | 11.27 | 12.12 | 13.03 |
| 19 | 8.30 | 9.00 | 9.73 | 10.56 | 11.45 | 12.30 | 13.22 |
| 20 | 8.46 | 9.17 | 9.90 | 10.74 | 11.63 | 12.49 | 13.41 |
| 21 | 8.62 | 9.34 | 10.07 | 10.92 | 11.82 | 12.68 | 13.62 |
| 22 | 8.78 | 9.50 | 10.25 | 11.10 | 12.01 | 12.89 | 13.83 |
| 23 | 8.93 | 9.66 | 10.42 | 11.29 | 12.20 | 13.09 | 14.04 |
| 24 | 9.08 | 9.82 | 10.58 | 11.46 | 12.40 | 13.29 | 14.26 |
| 25 | 9.22 | 9.97 | 10.75 | 11.64 | 12.59 | 13.50 | 14.48 |
| 26 | 9.36 | 10.12 | 10.91 | 11.82 | 12.78 | 13.71 | 14.71 |
| 27 | 9.50 | 10.28 | 11.08 | 12.00 | 12.98 | 13.92 | 14.93 |
| 28 | 9.64 | 10.43 | 11.24 | 12.18 | 13.17 | 14.12 | 15.15 |
| 29 | 9.78 | 10.58 | 11.41 | 12.35 | 13.36 | 14.32 | 15.36 |
| 30 | 9.93 | 10.74 | 11.57 | 12.53 | 13.54 | 14.52 | 15.57 |
| 31 | 10.09 | 10.90 | 11.74 | 12.71 | 13.73 | 14.71 | 15.77 |
| 32 | 10.25 | 11.07 | 11.91 | 12.88 | 13.91 | 14.90 | 15.97 |
| 33 | 10.41 | 11.23 | 12.09 | 13.06 | 14.10 | 15.09 | 16.16 |
| 34 | 10.57 | 11.41 | 12.26 | 13.25 | 14.29 | 15.29 | 16.36 |
| 35 | 10.74 | 11.58 | 12.44 | 13.43 | 14.48 | 15.48 | 16.56 |
| 36 | 10.91 | 11.76 | 12.62 | 13.62 | 14.67 | 15.68 | 16.76 |

# The Postnatal Growth Reference for Preterm Infants

## Head circumference(30w girls)

| Corrected age(months) | Centiles | | | | | | |
|---|---|---|---|---|---|---|---|
| | 3rd | 10rd | 25rd | 50rd | 75rd | 90rd | 97rd |
| 0 | 30.84 | 32.50 | 33.00 | 34.05 | 35.05 | 35.94 | 36.90 |
| 1 | 33.63 | 34.54 | 35.43 | 36.38 | 37.29 | 38.09 | 38.86 |
| 2 | 35.08 | 35.97 | 36.85 | 37.79 | 38.71 | 39.50 | 40.27 |
| 3 | 36.40 | 37.29 | 38.16 | 39.10 | 40.01 | 40.80 | 41.56 |
| 4 | 37.57 | 38.45 | 39.32 | 40.25 | 41.15 | 41.94 | 42.70 |
| 5 | 38.58 | 39.45 | 40.31 | 41.24 | 42.13 | 42.92 | 43.68 |
| 6 | 39.43 | 40.30 | 41.16 | 42.08 | 42.97 | 43.76 | 44.51 |
| 7 | 40.15 | 41.02 | 41.87 | 42.79 | 43.68 | 44.46 | 45.22 |
| 8 | 40.76 | 41.62 | 42.47 | 43.39 | 44.28 | 45.07 | 45.82 |
| 9 | 41.26 | 42.13 | 42.98 | 43.90 | 44.80 | 45.58 | 46.34 |
| 10 | 41.69 | 42.56 | 43.42 | 44.34 | 45.25 | 46.04 | 46.80 |
| 11 | 42.05 | 42.93 | 43.80 | 44.73 | 45.64 | 46.44 | 47.21 |
| 12 | 42.36 | 43.25 | 44.13 | 45.08 | 46.01 | 46.81 | 47.59 |
| 13 | 42.64 | 43.55 | 44.44 | 45.40 | 46.34 | 47.16 | 47.95 |
| 14 | 42.90 | 43.82 | 44.73 | 45.70 | 46.65 | 47.49 | 48.29 |
| 15 | 43.15 | 44.08 | 44.99 | 45.98 | 46.94 | 47.79 | 48.60 |
| 16 | 43.38 | 44.32 | 45.24 | 46.24 | 47.21 | 48.06 | 48.88 |
| 17 | 43.60 | 44.54 | 45.47 | 46.47 | 47.45 | 48.30 | 49.13 |
| 18 | 43.80 | 44.75 | 45.68 | 46.68 | 47.66 | 48.52 | 49.34 |
| 19 | 43.99 | 44.93 | 45.87 | 46.87 | 47.85 | 48.71 | 49.53 |
| 20 | 44.17 | 45.11 | 46.04 | 47.04 | 48.02 | 48.87 | 49.70 |
| 21 | 44.33 | 45.27 | 46.20 | 47.20 | 48.17 | 49.02 | 49.84 |
| 22 | 44.50 | 45.43 | 46.35 | 47.34 | 48.31 | 49.16 | 49.97 |
| 23 | 44.65 | 45.58 | 46.49 | 47.48 | 48.44 | 49.28 | 50.09 |
| 24 | 44.80 | 45.72 | 46.63 | 47.61 | 48.56 | 49.40 | 50.21 |
| 25 | 44.94 | 45.85 | 46.75 | 47.73 | 48.68 | 49.51 | 50.31 |
| 26 | 45.07 | 45.98 | 46.87 | 47.84 | 48.79 | 49.61 | 50.41 |
| 27 | 45.19 | 46.10 | 46.99 | 47.95 | 48.89 | 49.72 | 50.51 |
| 28 | 45.32 | 46.22 | 47.11 | 48.07 | 49.00 | 49.82 | 50.61 |
| 29 | 45.45 | 46.34 | 47.23 | 48.18 | 49.12 | 49.93 | 50.72 |
| 30 | 45.58 | 46.48 | 47.36 | 48.31 | 49.24 | 50.06 | 50.85 |
| 31 | 45.73 | 46.62 | 47.50 | 48.45 | 49.38 | 50.19 | 50.98 |
| 32 | 45.89 | 46.78 | 47.66 | 48.60 | 49.53 | 50.34 | 51.12 |
| 33 | 46.06 | 46.94 | 47.82 | 48.76 | 49.68 | 50.49 | 51.27 |
| 34 | 46.23 | 47.11 | 47.98 | 48.92 | 49.84 | 50.65 | 51.43 |
| 35 | 46.40 | 47.28 | 48.14 | 49.08 | 50.00 | 50.81 | 51.58 |
| 36 | 46.57 | 47.44 | 48.31 | 49.25 | 50.16 | 50.97 | 51.74 |

By Wuhan University Children’s Digital Health and Data Center (CDHDC)

# The Postnatal Growth Reference for Preterm Infants

WUHAN UNIVERSITY

## BMI(30w girls)

| Corrected age(months) | Centiles | | | | | | |
|---|---|---|---|---|---|---|---|
| | 3rd | 10rd | 25rd | 50rd | 75rd | 90rd | 97rd |
| 0 | 10.53 | 11.57 | 12.20 | 13.02 | 14.06 | 15.57 | 17.43 |
| 1 | 11.94 | 12.85 | 13.84 | 15.01 | 16.28 | 17.51 | 18.81 |
| 2 | 12.59 | 13.53 | 14.54 | 15.75 | 17.04 | 18.30 | 19.62 |
| 3 | 13.14 | 14.10 | 15.13 | 16.35 | 17.67 | 18.94 | 20.28 |
| 4 | 13.56 | 14.53 | 15.57 | 16.80 | 18.11 | 19.39 | 20.73 |
| 5 | 13.86 | 14.82 | 15.85 | 17.08 | 18.39 | 19.65 | 20.98 |
| 6 | 14.04 | 14.99 | 16.01 | 17.22 | 18.51 | 19.75 | 21.05 |
| 7 | 14.14 | 15.07 | 16.08 | 17.26 | 18.52 | 19.73 | 21.00 |
| 8 | 14.17 | 15.08 | 16.06 | 17.22 | 18.44 | 19.62 | 20.86 |
| 9 | 14.14 | 15.04 | 15.99 | 17.12 | 18.32 | 19.46 | 20.66 |
| 10 | 14.08 | 14.96 | 15.89 | 16.98 | 18.15 | 19.27 | 20.44 |
| 11 | 13.99 | 14.84 | 15.76 | 16.83 | 17.97 | 19.06 | 20.20 |
| 12 | 13.88 | 14.72 | 15.61 | 16.66 | 17.78 | 18.84 | 19.96 |
| 13 | 13.76 | 14.58 | 15.46 | 16.49 | 17.58 | 18.63 | 19.72 |
| 14 | 13.64 | 14.45 | 15.31 | 16.32 | 17.39 | 18.41 | 19.48 |
| 15 | 13.51 | 14.31 | 15.15 | 16.14 | 17.20 | 18.20 | 19.25 |
| 16 | 13.39 | 14.17 | 15.00 | 15.97 | 17.00 | 17.99 | 19.01 |
| 17 | 13.27 | 14.04 | 14.86 | 15.81 | 16.82 | 17.79 | 18.79 |
| 18 | 13.17 | 13.92 | 14.73 | 15.66 | 16.66 | 17.60 | 18.59 |
| 19 | 13.08 | 13.83 | 14.61 | 15.53 | 16.51 | 17.43 | 18.40 |
| 20 | 13.02 | 13.74 | 14.52 | 15.42 | 16.38 | 17.29 | 18.23 |
| 21 | 12.96 | 13.68 | 14.44 | 15.33 | 16.27 | 17.16 | 18.09 |
| 22 | 12.93 | 13.63 | 14.38 | 15.25 | 16.17 | 17.04 | 17.95 |
| 23 | 12.89 | 13.59 | 14.32 | 15.18 | 16.08 | 16.94 | 17.83 |
| 24 | 12.87 | 13.55 | 14.27 | 15.12 | 16.01 | 16.85 | 17.73 |
| 25 | 12.84 | 13.52 | 14.23 | 15.07 | 15.94 | 16.78 | 17.64 |
| 26 | 12.82 | 13.49 | 14.20 | 15.02 | 15.89 | 16.71 | 17.57 |
| 27 | 12.80 | 13.47 | 14.17 | 14.99 | 15.85 | 16.66 | 17.51 |
| 28 | 12.78 | 13.44 | 14.14 | 14.95 | 15.81 | 16.62 | 17.46 |
| 29 | 12.77 | 13.42 | 14.12 | 14.93 | 15.78 | 16.59 | 17.42 |
| 30 | 12.75 | 13.41 | 14.10 | 14.91 | 15.76 | 16.57 | 17.40 |
| 31 | 12.75 | 13.41 | 14.10 | 14.91 | 15.76 | 16.56 | 17.40 |
| 32 | 12.75 | 13.41 | 14.10 | 14.91 | 15.76 | 16.57 | 17.40 |
| 33 | 12.76 | 13.42 | 14.11 | 14.92 | 15.78 | 16.58 | 17.42 |
| 34 | 12.78 | 13.44 | 14.13 | 14.95 | 15.80 | 16.61 | 17.45 |
| 35 | 12.80 | 13.46 | 14.16 | 14.97 | 15.83 | 16.64 | 17.48 |
| 36 | 12.82 | 13.48 | 14.19 | 15.00 | 15.86 | 16.68 | 17.52 |

By Wuhan University Children's Digital Health and Data Center (CDHDC)

# The Postnatal Growth Reference for Preterm Infants

WUHAN UNIVERSITY

## Length (29w girls)

| Corrected age(months) | Centiles | | | | | | |
|---|---|---|---|---|---|---|---|
| | 3rd | 10rd | 25rd | 50rd | 75rd | 90rd | 97rd |
| 0 | 44.33 | 46.50 | 48.45 | 49.50 | 51.20 | 52.40 | 54.10 |
| 1 | 49.09 | 50.69 | 52.24 | 53.93 | 55.64 | 57.26 | 58.97 |
| 2 | 52.08 | 53.65 | 55.24 | 57.00 | 58.77 | 60.36 | 61.95 |
| 3 | 54.81 | 56.41 | 58.02 | 59.82 | 61.62 | 63.24 | 64.85 |
| 4 | 57.27 | 58.89 | 60.53 | 62.34 | 64.16 | 65.80 | 67.43 |
| 5 | 59.45 | 61.08 | 62.73 | 64.56 | 66.39 | 68.05 | 69.69 |
| 6 | 61.38 | 63.02 | 64.67 | 66.51 | 68.35 | 70.02 | 71.66 |
| 7 | 63.10 | 64.74 | 66.40 | 68.24 | 70.09 | 71.75 | 73.41 |
| 8 | 64.65 | 66.29 | 67.95 | 69.79 | 71.63 | 73.30 | 74.95 |
| 9 | 66.07 | 67.70 | 69.35 | 71.18 | 73.02 | 74.68 | 76.32 |
| 10 | 67.37 | 68.99 | 70.63 | 72.45 | 74.27 | 75.92 | 77.55 |
| 11 | 68.58 | 70.19 | 71.82 | 73.62 | 75.43 | 77.06 | 78.68 |
| 12 | 69.72 | 71.32 | 72.93 | 74.72 | 76.51 | 78.13 | 79.74 |
| 13 | 70.80 | 72.38 | 73.99 | 75.77 | 77.55 | 79.15 | 80.75 |
| 14 | 71.83 | 73.41 | 75.00 | 76.77 | 78.55 | 80.15 | 81.73 |
| 15 | 72.82 | 74.40 | 75.99 | 77.76 | 79.53 | 81.13 | 82.72 |
| 16 | 73.79 | 75.37 | 76.97 | 78.74 | 80.52 | 82.12 | 83.71 |
| 17 | 74.73 | 76.32 | 77.93 | 79.72 | 81.50 | 83.12 | 84.72 |
| 18 | 75.64 | 77.25 | 78.87 | 80.68 | 82.49 | 84.12 | 85.74 |
| 19 | 76.51 | 78.14 | 79.80 | 81.64 | 83.47 | 85.13 | 86.78 |
| 20 | 77.34 | 79.01 | 80.70 | 82.57 | 84.45 | 86.14 | 87.82 |
| 21 | 78.14 | 79.85 | 81.57 | 83.49 | 85.41 | 87.14 | 88.85 |
| 22 | 78.91 | 80.66 | 82.42 | 84.38 | 86.34 | 88.11 | 89.86 |
| 23 | 79.64 | 81.43 | 83.23 | 85.24 | 87.25 | 89.06 | 90.85 |
| 24 | 80.34 | 82.17 | 84.02 | 86.07 | 88.13 | 89.98 | 91.82 |
| 25 | 81.01 | 82.88 | 84.78 | 86.88 | 88.98 | 90.88 | 92.77 |
| 26 | 81.66 | 83.58 | 85.51 | 87.66 | 89.81 | 91.76 | 93.68 |
| 27 | 82.32 | 84.27 | 86.24 | 88.43 | 90.63 | 92.61 | 94.57 |
| 28 | 82.97 | 84.96 | 86.96 | 89.19 | 91.42 | 93.43 | 95.43 |
| 29 | 83.63 | 85.64 | 87.68 | 89.93 | 92.19 | 94.23 | 96.26 |
| 30 | 84.30 | 86.34 | 88.39 | 90.67 | 92.96 | 95.02 | 97.06 |
| 31 | 84.99 | 87.04 | 89.11 | 91.42 | 93.72 | 95.80 | 97.86 |
| 32 | 85.70 | 87.76 | 89.85 | 92.17 | 94.49 | 96.58 | 98.66 |
| 33 | 86.42 | 88.50 | 90.60 | 92.93 | 95.27 | 97.37 | 99.46 |
| 34 | 87.16 | 89.25 | 91.36 | 93.70 | 96.05 | 98.16 | 100.26 |
| 35 | 87.91 | 90.00 | 92.12 | 94.47 | 96.83 | 98.95 | 101.06 |
| 36 | 88.65 | 90.76 | 92.88 | 95.24 | 97.60 | 99.74 | 101.85 |

By Wuhan University Children's Digital Health and Data Center (CDHDC)

# The Postnatal Growth Reference for Preterm Infants

WUHAN UNIVERSITY

## Weight(29w girls)

| Corrected age(months) | Centiles | | | | | | |
|---|---|---|---|---|---|---|---|
| | 3rd | 10rd | 25rd | 50rd | 75rd | 90rd | 97rd |
| 0 | 2.24 | 2.59 | 2.84 | 3.33 | 3.74 | 4.05 | 4.44 |
| 1 | 3.13 | 3.52 | 3.93 | 4.41 | 4.92 | 5.39 | 5.88 |
| 2 | 3.80 | 4.24 | 4.71 | 5.26 | 5.84 | 6.38 | 6.93 |
| 3 | 4.41 | 4.90 | 5.41 | 6.02 | 6.65 | 7.24 | 7.84 |
| 4 | 4.93 | 5.46 | 6.01 | 6.65 | 7.33 | 7.95 | 8.60 |
| 5 | 5.37 | 5.92 | 6.51 | 7.18 | 7.88 | 8.54 | 9.21 |
| 6 | 5.75 | 6.32 | 6.92 | 7.61 | 8.34 | 9.02 | 9.71 |
| 7 | 6.06 | 6.64 | 7.26 | 7.97 | 8.72 | 9.41 | 10.11 |
| 8 | 6.33 | 6.92 | 7.55 | 8.27 | 9.03 | 9.73 | 10.45 |
| 9 | 6.55 | 7.16 | 7.79 | 8.53 | 9.29 | 10.00 | 10.72 |
| 10 | 6.76 | 7.37 | 8.00 | 8.74 | 9.51 | 10.22 | 10.95 |
| 11 | 6.94 | 7.55 | 8.20 | 8.94 | 9.70 | 10.42 | 11.15 |
| 12 | 7.12 | 7.73 | 8.37 | 9.12 | 9.88 | 10.60 | 11.32 |
| 13 | 7.29 | 7.90 | 8.55 | 9.29 | 10.06 | 10.77 | 11.50 |
| 14 | 7.45 | 8.07 | 8.71 | 9.46 | 10.23 | 10.94 | 11.67 |
| 15 | 7.62 | 8.24 | 8.89 | 9.63 | 10.40 | 11.12 | 11.85 |
| 16 | 7.78 | 8.40 | 9.06 | 9.81 | 10.59 | 11.31 | 12.05 |
| 17 | 7.94 | 8.57 | 9.24 | 10.00 | 10.79 | 11.52 | 12.26 |
| 18 | 8.10 | 8.74 | 9.42 | 10.19 | 10.99 | 11.73 | 12.48 |
| 19 | 8.26 | 8.91 | 9.60 | 10.38 | 11.19 | 11.95 | 12.71 |
| 20 | 8.42 | 9.08 | 9.78 | 10.58 | 11.40 | 12.17 | 12.95 |
| 21 | 8.57 | 9.25 | 9.96 | 10.77 | 11.61 | 12.39 | 13.19 |
| 22 | 8.72 | 9.41 | 10.13 | 10.96 | 11.82 | 12.62 | 13.42 |
| 23 | 8.86 | 9.57 | 10.30 | 11.15 | 12.02 | 12.83 | 13.66 |
| 24 | 9.00 | 9.71 | 10.46 | 11.33 | 12.22 | 13.04 | 13.88 |
| 25 | 9.13 | 9.86 | 10.62 | 11.49 | 12.40 | 13.24 | 14.09 |
| 26 | 9.25 | 9.99 | 10.76 | 11.65 | 12.57 | 13.42 | 14.29 |
| 27 | 9.37 | 10.12 | 10.90 | 11.80 | 12.73 | 13.59 | 14.47 |
| 28 | 9.49 | 10.25 | 11.04 | 11.94 | 12.88 | 13.75 | 14.64 |
| 29 | 9.61 | 10.37 | 11.17 | 12.08 | 13.03 | 13.91 | 14.80 |
| 30 | 9.73 | 10.49 | 11.30 | 12.22 | 13.18 | 14.07 | 14.97 |
| 31 | 9.84 | 10.62 | 11.43 | 12.37 | 13.33 | 14.22 | 15.13 |
| 32 | 9.97 | 10.75 | 11.57 | 12.51 | 13.49 | 14.39 | 15.30 |
| 33 | 10.10 | 10.89 | 11.71 | 12.66 | 13.65 | 14.56 | 15.48 |
| 34 | 10.23 | 11.03 | 11.86 | 12.82 | 13.81 | 14.73 | 15.66 |
| 35 | 10.37 | 11.17 | 12.02 | 12.98 | 13.98 | 14.90 | 15.84 |
| 36 | 10.51 | 11.32 | 12.17 | 13.14 | 14.14 | 15.07 | 16.02 |

By Wuhan University Children's Digital Health and Data Center (CDHDC)

# The Postnatal Growth Reference for Preterm Infants

WUHAN UNIVERSITY

## Head circumference(29w girls)

| Corrected age(months) | Centiles | | | | | | |
|---|---|---|---|---|---|---|---|
| | 3rd | 10rd | 25rd | 50rd | 75rd | 90rd | 97rd |
| 0 | 31.83 | 32.50 | 33.50 | 34.25 | 35.08 | 36.00 | 37.35 |
| 1 | 34.07 | 34.89 | 35.68 | 36.53 | 37.39 | 38.18 | 39.01 |
| 2 | 35.40 | 36.22 | 37.01 | 37.86 | 38.71 | 39.50 | 40.33 |
| 3 | 36.62 | 37.44 | 38.23 | 39.08 | 39.94 | 40.73 | 41.56 |
| 4 | 37.70 | 38.52 | 39.31 | 40.17 | 41.03 | 41.83 | 42.66 |
| 5 | 38.63 | 39.46 | 40.26 | 41.12 | 41.98 | 42.78 | 43.62 |
| 6 | 39.43 | 40.26 | 41.06 | 41.93 | 42.80 | 43.60 | 44.44 |
| 7 | 40.12 | 40.95 | 41.75 | 42.62 | 43.49 | 44.30 | 45.14 |
| 8 | 40.69 | 41.53 | 42.33 | 43.20 | 44.07 | 44.88 | 45.73 |
| 9 | 41.18 | 42.01 | 42.82 | 43.69 | 44.56 | 45.37 | 46.21 |
| 10 | 41.58 | 42.42 | 43.22 | 44.09 | 44.96 | 45.76 | 46.60 |
| 11 | 41.93 | 42.76 | 43.55 | 44.42 | 45.29 | 46.09 | 46.93 |
| 12 | 42.22 | 43.05 | 43.84 | 44.70 | 45.56 | 46.36 | 47.20 |
| 13 | 42.48 | 43.30 | 44.09 | 44.95 | 45.81 | 46.61 | 47.44 |
| 14 | 42.71 | 43.53 | 44.32 | 45.18 | 46.03 | 46.82 | 47.65 |
| 15 | 42.93 | 43.75 | 44.53 | 45.38 | 46.24 | 47.03 | 47.85 |
| 16 | 43.12 | 43.94 | 44.72 | 45.58 | 46.43 | 47.22 | 48.05 |
| 17 | 43.29 | 44.11 | 44.90 | 45.75 | 46.61 | 47.41 | 48.24 |
| 18 | 43.43 | 44.26 | 45.06 | 45.92 | 46.78 | 47.58 | 48.42 |
| 19 | 43.57 | 44.41 | 45.21 | 46.08 | 46.95 | 47.76 | 48.60 |
| 20 | 43.72 | 44.56 | 45.37 | 46.24 | 47.12 | 47.93 | 48.78 |
| 21 | 43.87 | 44.71 | 45.52 | 46.40 | 47.28 | 48.10 | 48.95 |
| 22 | 44.02 | 44.87 | 45.69 | 46.57 | 47.45 | 48.27 | 49.13 |
| 23 | 44.18 | 45.03 | 45.85 | 46.73 | 47.62 | 48.44 | 49.30 |
| 24 | 44.33 | 45.18 | 46.00 | 46.89 | 47.78 | 48.61 | 49.47 |
| 25 | 44.48 | 45.33 | 46.16 | 47.05 | 47.94 | 48.76 | 49.63 |
| 26 | 44.62 | 45.48 | 46.30 | 47.19 | 48.08 | 48.91 | 49.77 |
| 27 | 44.76 | 45.62 | 46.44 | 47.33 | 48.23 | 49.05 | 49.92 |
| 28 | 44.90 | 45.75 | 46.58 | 47.47 | 48.36 | 49.19 | 50.06 |
| 29 | 45.03 | 45.89 | 46.71 | 47.61 | 48.50 | 49.34 | 50.20 |
| 30 | 45.16 | 46.02 | 46.85 | 47.75 | 48.65 | 49.48 | 50.35 |
| 31 | 45.28 | 46.15 | 46.99 | 47.89 | 48.80 | 49.64 | 50.51 |
| 32 | 45.41 | 46.28 | 47.12 | 48.04 | 48.95 | 49.80 | 50.68 |
| 33 | 45.53 | 46.41 | 47.26 | 48.18 | 49.11 | 49.96 | 50.85 |
| 34 | 45.66 | 46.55 | 47.41 | 48.34 | 49.27 | 50.13 | 51.03 |
| 35 | 45.78 | 46.69 | 47.55 | 48.49 | 49.43 | 50.31 | 51.22 |
| 36 | 45.91 | 46.82 | 47.70 | 48.65 | 49.60 | 50.48 | 51.40 |

By Wuhan University Children's Digital Health and Data Center (CDHDC)

# The Postnatal Growth Reference for Preterm Infants

## BMI(29w girls)

| Corrected age(months) | Centiles | | | | | | |
|---|---|---|---|---|---|---|---|
| | 3rd | 10rd | 25rd | 50rd | 75rd | 90rd | 97rd |
| 0 | 10.07 | 11.25 | 12.20 | 13.25 | 14.23 | 15.07 | 16.15 |
| 1 | 12.08 | 13.04 | 14.04 | 15.21 | 16.44 | 17.60 | 18.78 |
| 2 | 12.75 | 13.70 | 14.71 | 15.88 | 17.11 | 18.26 | 19.44 |
| 3 | 13.29 | 14.25 | 15.25 | 16.42 | 17.65 | 18.79 | 19.96 |
| 4 | 13.69 | 14.64 | 15.65 | 16.81 | 18.02 | 19.16 | 20.32 |
| 5 | 13.95 | 14.89 | 15.89 | 17.04 | 18.24 | 19.36 | 20.51 |
| 6 | 14.09 | 15.02 | 16.00 | 17.14 | 18.32 | 19.42 | 20.55 |
| 7 | 14.14 | 15.06 | 16.02 | 17.14 | 18.29 | 19.37 | 20.48 |
| 8 | 14.13 | 15.02 | 15.96 | 17.05 | 18.18 | 19.23 | 20.31 |
| 9 | 14.06 | 14.93 | 15.85 | 16.91 | 18.01 | 19.03 | 20.08 |
| 10 | 13.97 | 14.82 | 15.71 | 16.74 | 17.81 | 18.80 | 19.82 |
| 11 | 13.87 | 14.69 | 15.56 | 16.56 | 17.60 | 18.57 | 19.55 |
| 12 | 13.76 | 14.56 | 15.41 | 16.39 | 17.39 | 18.33 | 19.29 |
| 13 | 13.65 | 14.44 | 15.26 | 16.23 | 17.20 | 18.12 | 19.05 |
| 14 | 13.55 | 14.32 | 15.13 | 16.08 | 17.03 | 17.92 | 18.84 |
| 15 | 13.45 | 14.21 | 15.01 | 15.94 | 16.88 | 17.76 | 18.66 |
| 16 | 13.36 | 14.12 | 14.90 | 15.83 | 16.75 | 17.62 | 18.51 |
| 17 | 13.28 | 14.03 | 14.81 | 15.72 | 16.64 | 17.51 | 18.39 |
| 18 | 13.21 | 13.95 | 14.73 | 15.63 | 16.55 | 17.41 | 18.29 |
| 19 | 13.15 | 13.89 | 14.66 | 15.56 | 16.47 | 17.32 | 18.20 |
| 20 | 13.10 | 13.83 | 14.60 | 15.49 | 16.40 | 17.25 | 18.11 |
| 21 | 13.05 | 13.79 | 14.55 | 15.42 | 16.34 | 17.18 | 18.04 |
| 22 | 13.02 | 13.75 | 14.51 | 15.36 | 16.29 | 17.13 | 17.98 |
| 23 | 12.99 | 13.71 | 14.47 | 15.31 | 16.23 | 17.07 | 17.92 |
| 24 | 12.96 | 13.67 | 14.42 | 15.26 | 16.18 | 17.01 | 17.85 |
| 25 | 12.92 | 13.63 | 14.37 | 15.22 | 16.11 | 16.93 | 17.76 |
| 26 | 12.88 | 13.58 | 14.32 | 15.19 | 16.03 | 16.84 | 17.66 |
| 27 | 12.83 | 13.53 | 14.25 | 15.13 | 15.94 | 16.74 | 17.55 |
| 28 | 12.78 | 13.47 | 14.18 | 15.07 | 15.85 | 16.64 | 17.43 |
| 29 | 12.72 | 13.40 | 14.11 | 15.01 | 15.75 | 16.53 | 17.32 |
| 30 | 12.66 | 13.33 | 14.03 | 14.94 | 15.66 | 16.43 | 17.21 |
| 31 | 12.61 | 13.27 | 13.96 | 14.88 | 15.64 | 16.34 | 17.12 |
| 32 | 12.56 | 13.21 | 13.90 | 14.83 | 15.54 | 16.26 | 17.03 |
| 33 | 12.51 | 13.17 | 13.85 | 14.81 | 15.44 | 16.19 | 16.95 |
| 34 | 12.48 | 13.13 | 13.80 | 14.81 | 15.52 | 16.12 | 16.88 |
| 35 | 12.45 | 13.09 | 13.76 | 14.82 | 15.53 | 16.07 | 16.82 |
| 36 | 12.42 | 13.06 | 13.73 | 14.84 | 15.58 | 16.01 | 16.76 |

By Wuhan University Children’s Digital Health and Data Center (CDHDC)

# The Postnatal Growth Reference for Preterm Infants

## Length (28w girls)

| Corrected age(months) | Centiles | | | | | | |
|---|---|---|---|---|---|---|---|
| | 3rd | 10rd | 25rd | 50rd | 75rd | 90rd | 97rd |
| 0 | 45.02 | 46.88 | 48.05 | 49.00 | 50.65 | 51.68 | 53.73 |
| 1 | 48.75 | 50.18 | 51.65 | 53.34 | 55.07 | 56.66 | 58.26 |
| 2 | 51.67 | 53.16 | 54.69 | 56.44 | 58.22 | 59.87 | 61.54 |
| 3 | 54.42 | 55.94 | 57.52 | 59.32 | 61.16 | 62.85 | 64.56 |
| 4 | 56.90 | 58.46 | 60.07 | 61.89 | 63.76 | 65.48 | 67.22 |
| 5 | 59.10 | 60.67 | 62.30 | 64.14 | 66.03 | 67.76 | 69.51 |
| 6 | 61.05 | 62.62 | 64.26 | 66.11 | 68.01 | 69.75 | 71.50 |
| 7 | 62.76 | 64.35 | 65.99 | 67.85 | 69.75 | 71.50 | 73.26 |
| 8 | 64.28 | 65.88 | 67.53 | 69.40 | 71.31 | 73.07 | 74.84 |
| 9 | 65.63 | 67.24 | 68.91 | 70.80 | 72.73 | 74.50 | 76.28 |
| 10 | 66.86 | 68.49 | 70.17 | 72.08 | 74.03 | 75.81 | 77.61 |
| 11 | 68.00 | 69.65 | 71.35 | 73.28 | 75.25 | 77.06 | 78.88 |
| 12 | 69.07 | 70.74 | 72.46 | 74.42 | 76.41 | 78.24 | 80.08 |
| 13 | 70.09 | 71.78 | 73.52 | 75.50 | 77.52 | 79.37 | 81.23 |
| 14 | 71.07 | 72.77 | 74.54 | 76.54 | 78.58 | 80.45 | 82.33 |
| 15 | 72.02 | 73.74 | 75.52 | 77.54 | 79.60 | 81.49 | 83.39 |
| 16 | 72.97 | 74.70 | 76.49 | 78.52 | 80.59 | 82.50 | 84.41 |
| 17 | 73.92 | 75.66 | 77.46 | 79.50 | 81.58 | 83.49 | 85.41 |
| 18 | 74.87 | 76.62 | 78.43 | 80.47 | 82.56 | 84.48 | 86.41 |
| 19 | 75.81 | 77.57 | 79.39 | 81.44 | 83.54 | 85.46 | 87.39 |
| 20 | 76.76 | 78.52 | 80.34 | 82.40 | 84.50 | 86.43 | 88.36 |
| 21 | 77.69 | 79.45 | 81.28 | 83.34 | 85.45 | 87.38 | 89.32 |
| 22 | 78.58 | 80.36 | 82.18 | 84.25 | 86.37 | 88.30 | 90.24 |
| 23 | 79.43 | 81.22 | 83.05 | 85.13 | 87.25 | 89.19 | 91.14 |
| 24 | 80.23 | 82.03 | 83.88 | 85.97 | 88.10 | 90.05 | 92.02 |
| 25 | 80.98 | 82.79 | 84.65 | 86.76 | 88.91 | 90.88 | 92.86 |
| 26 | 81.68 | 83.50 | 85.38 | 87.51 | 89.68 | 91.67 | 93.67 |
| 27 | 82.33 | 84.17 | 86.08 | 88.23 | 90.42 | 92.43 | 94.45 |
| 28 | 82.94 | 84.81 | 86.73 | 88.91 | 91.13 | 93.17 | 95.21 |
| 29 | 83.51 | 85.41 | 87.36 | 89.56 | 91.81 | 93.88 | 95.95 |
| 30 | 84.05 | 85.96 | 87.94 | 90.18 | 92.47 | 94.56 | 96.67 |
| 31 | 84.54 | 86.49 | 88.50 | 90.78 | 93.10 | 95.23 | 97.37 |
| 32 | 85.02 | 87.00 | 89.05 | 91.36 | 93.73 | 95.90 | 98.08 |
| 33 | 85.47 | 87.49 | 89.58 | 91.93 | 94.34 | 96.55 | 98.77 |
| 34 | 85.92 | 87.97 | 90.10 | 92.50 | 94.95 | 97.21 | 99.47 |
| 35 | 86.35 | 88.45 | 90.61 | 93.06 | 95.56 | 97.86 | 100.17 |
| 36 | 86.79 | 88.92 | 91.12 | 93.62 | 96.18 | 98.52 | 100.88 |

# The Postnatal Growth Reference for Preterm Infants

WUHAN UNIVERSITY

## Weight(28w girls)

| Corrected age(months) | Centiles | | | | | | |
|---|---|---|---|---|---|---|---|
| | 3rd | 10rd | 25rd | 50rd | 75rd | 90rd | 97rd |
| 0 | 2.38 | 2.65 | 2.90 | 3.16 | 3.43 | 3.82 | 4.15 |
| 1 | 3.06 | 3.42 | 3.81 | 4.26 | 4.74 | 5.19 | 5.65 |
| 2 | 3.67 | 4.11 | 4.57 | 5.11 | 5.68 | 6.22 | 6.76 |
| 3 | 4.23 | 4.73 | 5.26 | 5.87 | 6.52 | 7.13 | 7.75 |
| 4 | 4.71 | 5.26 | 5.84 | 6.52 | 7.23 | 7.90 | 8.58 |
| 5 | 5.13 | 5.71 | 6.33 | 7.06 | 7.82 | 8.53 | 9.26 |
| 6 | 5.48 | 6.10 | 6.75 | 7.50 | 8.29 | 9.04 | 9.80 |
| 7 | 5.80 | 6.43 | 7.10 | 7.88 | 8.69 | 9.45 | 10.23 |
| 8 | 6.07 | 6.72 | 7.40 | 8.19 | 9.02 | 9.79 | 10.59 |
| 9 | 6.32 | 6.98 | 7.67 | 8.47 | 9.30 | 10.08 | 10.88 |
| 10 | 6.55 | 7.21 | 7.90 | 8.71 | 9.55 | 10.33 | 11.13 |
| 11 | 6.75 | 7.41 | 8.11 | 8.92 | 9.76 | 10.55 | 11.36 |
| 12 | 6.92 | 7.59 | 8.30 | 9.11 | 9.96 | 10.75 | 11.56 |
| 13 | 7.08 | 7.75 | 8.46 | 9.29 | 10.14 | 10.94 | 11.75 |
| 14 | 7.22 | 7.90 | 8.62 | 9.45 | 10.31 | 11.11 | 11.93 |
| 15 | 7.36 | 8.04 | 8.76 | 9.60 | 10.46 | 11.27 | 12.10 |
| 16 | 7.49 | 8.18 | 8.90 | 9.74 | 10.62 | 11.43 | 12.26 |
| 17 | 7.62 | 8.32 | 9.05 | 9.89 | 10.77 | 11.58 | 12.42 |
| 18 | 7.77 | 8.46 | 9.20 | 10.04 | 10.92 | 11.74 | 12.57 |
| 19 | 7.92 | 8.61 | 9.35 | 10.19 | 11.07 | 11.89 | 12.72 |
| 20 | 8.07 | 8.77 | 9.50 | 10.35 | 11.23 | 12.04 | 12.87 |
| 21 | 8.23 | 8.93 | 9.66 | 10.51 | 11.39 | 12.20 | 13.03 |
| 22 | 8.39 | 9.09 | 9.82 | 10.67 | 11.55 | 12.36 | 13.19 |
| 23 | 8.54 | 9.24 | 9.98 | 10.83 | 11.71 | 12.53 | 13.37 |
| 24 | 8.68 | 9.39 | 10.13 | 10.99 | 11.88 | 12.71 | 13.55 |
| 25 | 8.81 | 9.53 | 10.28 | 11.15 | 12.05 | 12.89 | 13.74 |
| 26 | 8.93 | 9.66 | 10.43 | 11.31 | 12.23 | 13.08 | 13.94 |
| 27 | 9.05 | 9.79 | 10.57 | 11.47 | 12.41 | 13.27 | 14.15 |
| 28 | 9.16 | 9.92 | 10.72 | 11.64 | 12.59 | 13.47 | 14.37 |
| 29 | 9.27 | 10.05 | 10.86 | 11.80 | 12.77 | 13.67 | 14.58 |
| 30 | 9.39 | 10.17 | 11.00 | 11.96 | 12.95 | 13.87 | 14.80 |
| 31 | 9.50 | 10.30 | 11.15 | 12.12 | 13.13 | 14.07 | 15.02 |
| 32 | 9.62 | 10.44 | 11.30 | 12.29 | 13.32 | 14.27 | 15.25 |
| 33 | 9.73 | 10.57 | 11.44 | 12.45 | 13.50 | 14.48 | 15.47 |
| 34 | 9.85 | 10.70 | 11.59 | 12.62 | 13.69 | 14.68 | 15.69 |
| 35 | 9.97 | 10.83 | 11.74 | 12.78 | 13.87 | 14.88 | 15.90 |
| 36 | 10.09 | 10.96 | 11.89 | 12.95 | 14.05 | 15.08 | 16.12 |

By Wuhan University Children's Digital Health and Data Center (CDHDC)

# The Postnatal Growth Reference for Preterm Infants

WUHAN UNIVERSITY

## Head circumference(28w girls)

| Corrected age(months) | Centiles | | | | | | |
|---|---|---|---|---|---|---|---|
| | 3rd | 10rd | 25rd | 50rd | 75rd | 90rd | 97rd |
| 0 | 31.20 | 31.68 | 32.75 | 33.50 | 34.30 | 34.66 | 35.26 |
| 1 | 33.49 | 34.29 | 35.10 | 36.03 | 37.06 | 38.12 | 39.37 |
| 2 | 34.93 | 35.73 | 36.53 | 37.45 | 38.46 | 39.49 | 40.71 |
| 3 | 36.28 | 37.07 | 37.86 | 38.77 | 39.76 | 40.77 | 41.96 |
| 4 | 37.48 | 38.26 | 39.05 | 39.94 | 40.91 | 41.90 | 43.05 |
| 5 | 38.51 | 39.29 | 40.07 | 40.95 | 41.90 | 42.86 | 43.99 |
| 6 | 39.39 | 40.16 | 40.93 | 41.80 | 42.73 | 43.68 | 44.78 |
| 7 | 40.13 | 40.89 | 41.65 | 42.51 | 43.43 | 44.36 | 45.44 |
| 8 | 40.75 | 41.51 | 42.26 | 43.11 | 44.02 | 44.93 | 45.99 |
| 9 | 41.28 | 42.03 | 42.78 | 43.62 | 44.52 | 45.43 | 46.47 |
| 10 | 41.73 | 42.49 | 43.23 | 44.06 | 44.96 | 45.86 | 46.90 |
| 11 | 42.12 | 42.87 | 43.62 | 44.45 | 45.34 | 46.24 | 47.27 |
| 12 | 42.46 | 43.21 | 43.95 | 44.79 | 45.68 | 46.58 | 47.61 |
| 13 | 42.76 | 43.51 | 44.26 | 45.09 | 45.99 | 46.89 | 47.92 |
| 14 | 43.02 | 43.78 | 44.52 | 45.37 | 46.26 | 47.17 | 48.21 |
| 15 | 43.25 | 44.01 | 44.76 | 45.61 | 46.51 | 47.42 | 48.46 |
| 16 | 43.46 | 44.22 | 44.97 | 45.82 | 46.73 | 47.64 | 48.69 |
| 17 | 43.65 | 44.41 | 45.17 | 46.02 | 46.93 | 47.85 | 48.90 |
| 18 | 43.83 | 44.60 | 45.35 | 46.21 | 47.12 | 48.03 | 49.08 |
| 19 | 44.01 | 44.78 | 45.54 | 46.39 | 47.30 | 48.21 | 49.26 |
| 20 | 44.20 | 44.96 | 45.72 | 46.57 | 47.47 | 48.38 | 49.42 |
| 21 | 44.39 | 45.15 | 45.90 | 46.74 | 47.64 | 48.55 | 49.58 |
| 22 | 44.58 | 45.33 | 46.08 | 46.92 | 47.82 | 48.72 | 49.75 |
| 23 | 44.76 | 45.51 | 46.26 | 47.10 | 47.99 | 48.89 | 49.92 |
| 24 | 44.93 | 45.69 | 46.44 | 47.28 | 48.17 | 49.07 | 50.09 |
| 25 | 45.10 | 45.86 | 46.61 | 47.45 | 48.34 | 49.24 | 50.27 |
| 26 | 45.25 | 46.01 | 46.76 | 47.61 | 48.50 | 49.40 | 50.43 |
| 27 | 45.38 | 46.15 | 46.90 | 47.75 | 48.65 | 49.55 | 50.59 |
| 28 | 45.49 | 46.26 | 47.02 | 47.87 | 48.77 | 49.68 | 50.72 |
| 29 | 45.59 | 46.36 | 47.12 | 47.97 | 48.88 | 49.79 | 50.83 |
| 30 | 45.67 | 46.44 | 47.20 | 48.05 | 48.96 | 49.87 | 50.92 |
| 31 | 45.74 | 46.51 | 47.27 | 48.13 | 49.04 | 49.95 | 50.99 |
| 32 | 45.81 | 46.58 | 47.34 | 48.20 | 49.11 | 50.02 | 51.06 |
| 33 | 45.88 | 46.65 | 47.41 | 48.26 | 49.17 | 50.08 | 51.12 |
| 34 | 45.95 | 46.72 | 47.47 | 48.32 | 49.23 | 50.13 | 51.17 |
| 35 | 46.02 | 46.78 | 47.54 | 48.39 | 49.29 | 50.19 | 51.22 |
| 36 | 46.09 | 46.85 | 47.61 | 48.45 | 49.35 | 50.25 | 51.27 |

By Wuhan University Children's Digital Health and Data Center (CDHDC)

# The Postnatal Growth Reference for Preterm Infants

WUHAN UNIVERSITY

## BMI(28w girls)

| Corrected age(months) | Centiles | | | | | | |
|---|---|---|---|---|---|---|---|
| | 3rd | 10rd | 25rd | 50rd | 75rd | 90rd | 97rd |
| 0 | 11.45 | 12.33 | 13.29 | 13.18 | 15.64 | 16.81 | 18.04 |
| 1 | 12.03 | 12.94 | 13.92 | 15.09 | 16.34 | 17.53 | 18.79 |
| 2 | 12.57 | 13.51 | 14.51 | 15.71 | 16.98 | 18.20 | 19.48 |
| 3 | 13.03 | 13.99 | 15.01 | 16.22 | 17.52 | 18.76 | 20.06 |
| 4 | 13.39 | 14.35 | 15.38 | 16.61 | 17.91 | 19.17 | 20.47 |
| 5 | 13.62 | 14.59 | 15.63 | 16.86 | 18.17 | 19.42 | 20.73 |
| 6 | 13.77 | 14.73 | 15.77 | 16.99 | 18.29 | 19.54 | 20.84 |
| 7 | 13.84 | 14.80 | 15.82 | 17.03 | 18.31 | 19.54 | 20.82 |
| 8 | 13.87 | 14.81 | 15.81 | 16.99 | 18.25 | 19.45 | 20.70 |
| 9 | 13.86 | 14.78 | 15.76 | 16.91 | 18.13 | 19.30 | 20.51 |
| 10 | 13.82 | 14.71 | 15.66 | 16.78 | 17.97 | 19.10 | 20.28 |
| 11 | 13.75 | 14.61 | 15.54 | 16.63 | 17.78 | 18.88 | 20.02 |
| 12 | 13.65 | 14.50 | 15.40 | 16.46 | 17.58 | 18.64 | 19.75 |
| 13 | 13.54 | 14.36 | 15.24 | 16.28 | 17.37 | 18.41 | 19.48 |
| 14 | 13.41 | 14.22 | 15.08 | 16.09 | 17.16 | 18.17 | 19.22 |
| 15 | 13.29 | 14.08 | 14.93 | 15.91 | 16.96 | 17.95 | 18.97 |
| 16 | 13.17 | 13.95 | 14.77 | 15.74 | 16.76 | 17.73 | 18.73 |
| 17 | 13.07 | 13.83 | 14.63 | 15.58 | 16.58 | 17.52 | 18.50 |
| 18 | 12.98 | 13.72 | 14.51 | 15.43 | 16.40 | 17.32 | 18.27 |
| 19 | 12.90 | 13.63 | 14.40 | 15.30 | 16.24 | 17.13 | 18.06 |
| 20 | 12.85 | 13.55 | 14.30 | 15.17 | 16.09 | 16.96 | 17.85 |
| 21 | 12.80 | 13.49 | 14.22 | 15.07 | 15.96 | 16.80 | 17.67 |
| 22 | 12.76 | 13.43 | 14.14 | 14.97 | 15.84 | 16.66 | 17.51 |
| 23 | 12.72 | 13.38 | 14.08 | 14.89 | 15.74 | 16.55 | 17.38 |
| 24 | 12.67 | 13.33 | 14.02 | 14.82 | 15.66 | 16.45 | 17.27 |
| 25 | 12.63 | 13.28 | 13.96 | 14.76 | 15.59 | 16.38 | 17.19 |
| 26 | 12.58 | 13.23 | 13.91 | 14.71 | 15.54 | 16.33 | 17.13 |
| 27 | 12.54 | 13.19 | 13.87 | 14.67 | 15.50 | 16.29 | 17.10 |
| 28 | 12.50 | 13.15 | 13.84 | 14.64 | 15.48 | 16.27 | 17.09 |
| 29 | 12.46 | 13.11 | 13.81 | 14.62 | 15.47 | 16.27 | 17.10 |
| 30 | 12.42 | 13.09 | 13.79 | 14.61 | 15.48 | 16.29 | 17.13 |
| 31 | 12.40 | 13.07 | 13.79 | 14.62 | 15.50 | 16.33 | 17.18 |
| 32 | 12.38 | 13.06 | 13.79 | 14.64 | 15.53 | 16.38 | 17.25 |
| 33 | 12.35 | 13.05 | 13.79 | 14.66 | 15.57 | 16.44 | 17.33 |
| 34 | 12.33 | 13.04 | 13.80 | 14.68 | 15.61 | 16.50 | 17.41 |
| 35 | 12.30 | 13.02 | 13.80 | 14.70 | 15.66 | 16.56 | 17.50 |
| 36 | 12.26 | 13.00 | 13.79 | 14.72 | 15.70 | 16.63 | 17.59 |

By Wuhan University Children's Digital Health and Data Center (CDHDC)

# The Postnatal Growth Reference for Preterm Infants

WUHAN UNIVERSITY

## Length (<28w girls))

| Corrected age(months) | Centiles | | | | | | |
|---|---|---|---|---|---|---|---|
| | 3rd | 10rd | 25rd | 50rd | 75rd | 90rd | 97rd |
| 0 | 43.00 | 46.00 | 47.50 | 48.50 | 49.60 | 50.20 | 53.28 |
| 1 | 47.93 | 49.67 | 51.38 | 53.22 | 55.02 | 56.59 | 58.11 |
| 2 | 50.98 | 52.76 | 54.51 | 56.40 | 58.24 | 59.85 | 61.41 |
| 3 | 53.83 | 55.63 | 57.41 | 59.33 | 61.20 | 62.85 | 64.44 |
| 4 | 56.40 | 58.21 | 60.00 | 61.94 | 63.83 | 65.50 | 67.10 |
| 5 | 58.65 | 60.48 | 62.28 | 64.23 | 66.14 | 67.81 | 69.43 |
| 6 | 60.62 | 62.46 | 64.27 | 66.24 | 68.15 | 69.84 | 71.48 |
| 7 | 62.37 | 64.21 | 66.04 | 68.02 | 69.95 | 71.66 | 73.31 |
| 8 | 63.94 | 65.79 | 67.63 | 69.63 | 71.58 | 73.29 | 74.96 |
| 9 | 65.37 | 67.24 | 69.09 | 71.09 | 73.05 | 74.78 | 76.46 |
| 10 | 66.70 | 68.57 | 70.43 | 72.44 | 74.42 | 76.15 | 77.84 |
| 11 | 67.94 | 69.82 | 71.68 | 73.70 | 75.68 | 77.42 | 79.11 |
| 12 | 69.10 | 70.98 | 72.85 | 74.88 | 76.86 | 78.61 | 80.30 |
| 13 | 70.20 | 72.09 | 73.96 | 75.99 | 77.98 | 79.73 | 81.43 |
| 14 | 71.26 | 73.15 | 75.03 | 77.07 | 79.06 | 80.82 | 82.53 |
| 15 | 72.27 | 74.18 | 76.06 | 78.12 | 80.12 | 81.90 | 83.62 |
| 16 | 73.27 | 75.19 | 77.09 | 79.16 | 81.19 | 82.98 | 84.72 |
| 17 | 74.23 | 76.17 | 78.10 | 80.20 | 82.25 | 84.06 | 85.82 |
| 18 | 75.14 | 77.11 | 79.07 | 81.20 | 83.29 | 85.12 | 86.91 |
| 19 | 76.01 | 78.01 | 80.00 | 82.16 | 84.27 | 86.14 | 87.95 |
| 20 | 76.83 | 78.86 | 80.88 | 83.07 | 85.22 | 87.11 | 88.95 |
| 21 | 77.62 | 79.68 | 81.72 | 83.94 | 86.12 | 88.04 | 89.90 |
| 22 | 78.37 | 80.46 | 82.52 | 84.77 | 86.97 | 88.91 | 90.79 |
| 23 | 79.08 | 81.18 | 83.27 | 85.54 | 87.77 | 89.73 | 91.63 |
| 24 | 79.74 | 81.87 | 83.98 | 86.28 | 88.52 | 90.50 | 92.43 |
| 25 | 80.36 | 82.51 | 84.65 | 86.98 | 89.25 | 91.26 | 93.20 |
| 26 | 80.94 | 83.13 | 85.30 | 87.65 | 89.96 | 91.99 | 93.96 |
| 27 | 81.52 | 83.73 | 85.93 | 88.32 | 90.66 | 92.72 | 94.71 |
| 28 | 82.08 | 84.33 | 86.56 | 88.98 | 91.35 | 93.43 | 95.46 |
| 29 | 82.65 | 84.93 | 87.18 | 89.64 | 92.03 | 94.15 | 96.20 |
| 30 | 83.23 | 85.53 | 87.82 | 90.30 | 92.72 | 94.86 | 96.93 |
| 31 | 83.82 | 86.15 | 88.46 | 90.96 | 93.42 | 95.58 | 97.67 |
| 32 | 84.43 | 86.78 | 89.11 | 91.64 | 94.12 | 96.30 | 98.42 |
| 33 | 85.05 | 87.42 | 89.78 | 92.33 | 94.83 | 97.03 | 99.17 |
| 34 | 85.67 | 88.07 | 90.45 | 93.03 | 95.55 | 97.77 | 99.93 |
| 35 | 86.30 | 88.73 | 91.13 | 93.73 | 96.28 | 98.52 | 100.70 |
| 36 | 86.93 | 89.38 | 91.80 | 94.44 | 97.01 | 99.28 | 101.47 |

By Wuhan University Children’s Digital Health and Data Center (CDHDC)

# The Postnatal Growth Reference for Preterm Infants

WUHAN UNIVERSITY

## Weight(<28w girls))

| Corrected age(months) | Centiles | | | | | | |
|---|---|---|---|---|---|---|---|
| | 3rd | 10rd | 25rd | 50rd | 75rd | 90rd | 97rd |
| 0 | 2.00 | 2.32 | 2.56 | 3.10 | 3.34 | 3.60 | 3.88 |
| 1 | 2.70 | 3.18 | 3.65 | 4.17 | 4.68 | 5.13 | 5.57 |
| 2 | 3.34 | 3.88 | 4.41 | 4.99 | 5.56 | 6.07 | 6.57 |
| 3 | 3.95 | 4.53 | 5.10 | 5.73 | 6.34 | 6.89 | 7.43 |
| 4 | 4.52 | 5.12 | 5.72 | 6.38 | 7.03 | 7.60 | 8.17 |
| 5 | 5.03 | 5.65 | 6.27 | 6.95 | 7.62 | 8.21 | 8.80 |
| 6 | 5.48 | 6.11 | 6.75 | 7.44 | 8.12 | 8.73 | 9.33 |
| 7 | 5.86 | 6.51 | 7.15 | 7.86 | 8.56 | 9.18 | 9.79 |
| 8 | 6.19 | 6.84 | 7.50 | 8.22 | 8.93 | 9.56 | 10.18 |
| 9 | 6.45 | 7.12 | 7.79 | 8.52 | 9.24 | 9.89 | 10.52 |
| 10 | 6.67 | 7.35 | 8.03 | 8.78 | 9.51 | 10.17 | 10.82 |
| 11 | 6.85 | 7.54 | 8.23 | 8.99 | 9.75 | 10.42 | 11.08 |
| 12 | 6.99 | 7.70 | 8.40 | 9.18 | 9.95 | 10.63 | 11.30 |
| 13 | 7.11 | 7.83 | 8.55 | 9.34 | 10.12 | 10.82 | 11.50 |
| 14 | 7.22 | 7.95 | 8.68 | 9.48 | 10.27 | 10.98 | 11.67 |
| 15 | 7.32 | 8.06 | 8.80 | 9.61 | 10.42 | 11.13 | 11.83 |
| 16 | 7.42 | 8.17 | 8.92 | 9.74 | 10.56 | 11.28 | 11.99 |
| 17 | 7.52 | 8.28 | 9.04 | 9.87 | 10.70 | 11.43 | 12.15 |
| 18 | 7.64 | 8.40 | 9.17 | 10.01 | 10.85 | 11.59 | 12.32 |
| 19 | 7.76 | 8.54 | 9.31 | 10.17 | 11.01 | 11.76 | 12.50 |
| 20 | 7.89 | 8.68 | 9.46 | 10.33 | 11.18 | 11.94 | 12.68 |
| 21 | 8.03 | 8.83 | 9.62 | 10.49 | 11.35 | 12.12 | 12.87 |
| 22 | 8.17 | 8.98 | 9.78 | 10.66 | 11.53 | 12.30 | 13.06 |
| 23 | 8.31 | 9.12 | 9.93 | 10.82 | 11.70 | 12.49 | 13.25 |
| 24 | 8.44 | 9.26 | 10.08 | 10.98 | 11.87 | 12.67 | 13.44 |
| 25 | 8.57 | 9.40 | 10.23 | 11.14 | 12.04 | 12.84 | 13.63 |
| 26 | 8.68 | 9.52 | 10.37 | 11.29 | 12.20 | 13.02 | 13.82 |
| 27 | 8.80 | 9.65 | 10.50 | 11.44 | 12.36 | 13.19 | 14.00 |
| 28 | 8.92 | 9.78 | 10.64 | 11.58 | 12.52 | 13.36 | 14.17 |
| 29 | 9.03 | 9.90 | 10.77 | 11.73 | 12.68 | 13.52 | 14.35 |
| 30 | 9.14 | 10.03 | 10.91 | 11.88 | 12.84 | 13.70 | 14.54 |
| 31 | 9.26 | 10.15 | 11.05 | 12.04 | 13.01 | 13.88 | 14.73 |
| 32 | 9.38 | 10.29 | 11.20 | 12.20 | 13.19 | 14.07 | 14.93 |
| 33 | 9.50 | 10.42 | 11.35 | 12.36 | 13.37 | 14.27 | 15.14 |
| 34 | 9.62 | 10.56 | 11.50 | 12.53 | 13.55 | 14.46 | 15.36 |
| 35 | 9.74 | 10.69 | 11.65 | 12.70 | 13.74 | 14.66 | 15.57 |
| 36 | 9.86 | 10.83 | 11.80 | 12.87 | 13.92 | 14.86 | 15.78 |

By Wuhan University Children’s Digital Health and Data Center (CDHDC)

# The Postnatal Growth Reference for Preterm Infants

WUHAN UNIVERSITY

## Head circumference(<28w girls))

| Corrected age(months) | Centiles | | | | | | |
|---|---|---|---|---|---|---|---|
| | 3rd | 10rd | 25rd | 50rd | 75rd | 90rd | 97rd |
| 0 | 31.00 | 31.60 | 32.18 | 33.15 | 34.58 | 35.60 | 36.58 |
| 1 | 32.79 | 33.60 | 34.44 | 35.41 | 36.42 | 37.36 | 38.32 |
| 2 | 34.21 | 35.02 | 35.86 | 36.82 | 37.83 | 38.77 | 39.72 |
| 3 | 35.52 | 36.33 | 37.17 | 38.13 | 39.14 | 40.07 | 41.02 |
| 4 | 36.68 | 37.49 | 38.33 | 39.30 | 40.30 | 41.24 | 42.19 |
| 5 | 37.69 | 38.50 | 39.35 | 40.32 | 41.33 | 42.26 | 43.22 |
| 6 | 38.55 | 39.37 | 40.23 | 41.21 | 42.22 | 43.16 | 44.12 |
| 7 | 39.30 | 40.13 | 40.99 | 41.97 | 42.99 | 43.94 | 44.90 |
| 8 | 39.97 | 40.79 | 41.65 | 42.64 | 43.66 | 44.60 | 45.57 |
| 9 | 40.56 | 41.38 | 42.24 | 43.22 | 44.24 | 45.18 | 46.14 |
| 10 | 41.09 | 41.91 | 42.76 | 43.74 | 44.75 | 45.68 | 46.63 |
| 11 | 41.58 | 42.39 | 43.23 | 44.20 | 45.19 | 46.12 | 47.05 |
| 12 | 42.02 | 42.82 | 43.65 | 44.60 | 45.58 | 46.49 | 47.42 |
| 13 | 42.42 | 43.20 | 44.03 | 44.96 | 45.93 | 46.82 | 47.73 |
| 14 | 42.77 | 43.55 | 44.36 | 45.29 | 46.24 | 47.12 | 48.01 |
| 15 | 43.10 | 43.87 | 44.67 | 45.58 | 46.51 | 47.38 | 48.26 |
| 16 | 43.39 | 44.15 | 44.94 | 45.84 | 46.77 | 47.62 | 48.49 |
| 17 | 43.64 | 44.40 | 45.18 | 46.07 | 46.99 | 47.84 | 48.69 |
| 18 | 43.86 | 44.61 | 45.39 | 46.27 | 47.18 | 48.03 | 48.88 |
| 19 | 44.05 | 44.80 | 45.57 | 46.46 | 47.36 | 48.20 | 49.05 |
| 20 | 44.23 | 44.97 | 45.75 | 46.63 | 47.54 | 48.37 | 49.22 |
| 21 | 44.40 | 45.15 | 45.92 | 46.80 | 47.71 | 48.55 | 49.39 |
| 22 | 44.56 | 45.31 | 46.09 | 46.97 | 47.88 | 48.72 | 49.56 |
| 23 | 44.72 | 45.47 | 46.25 | 47.13 | 48.04 | 48.88 | 49.73 |
| 24 | 44.85 | 45.61 | 46.39 | 47.28 | 48.19 | 49.04 | 49.89 |
| 25 | 44.97 | 45.73 | 46.52 | 47.41 | 48.33 | 49.18 | 50.04 |
| 26 | 45.07 | 45.84 | 46.63 | 47.53 | 48.46 | 49.32 | 50.18 |
| 27 | 45.16 | 45.93 | 46.73 | 47.64 | 48.58 | 49.44 | 50.32 |
| 28 | 45.25 | 46.02 | 46.83 | 47.75 | 48.69 | 49.56 | 50.45 |
| 29 | 45.33 | 46.11 | 46.93 | 47.85 | 48.81 | 49.69 | 50.58 |
| 30 | 45.41 | 46.21 | 47.03 | 47.97 | 48.93 | 49.82 | 50.72 |
| 31 | 45.50 | 46.30 | 47.13 | 48.08 | 49.06 | 49.96 | 50.87 |
| 32 | 45.60 | 46.41 | 47.25 | 48.20 | 49.19 | 50.10 | 51.03 |
| 33 | 45.70 | 46.51 | 47.36 | 48.33 | 49.33 | 50.25 | 51.19 |
| 34 | 45.80 | 46.63 | 47.49 | 48.47 | 49.48 | 50.41 | 51.36 |
| 35 | 45.91 | 46.75 | 47.61 | 48.61 | 49.63 | 50.57 | 51.53 |
| 36 | 46.02 | 46.87 | 47.74 | 48.74 | 49.78 | 50.73 | 51.70 |

By Wuhan University Children’s Digital Health and Data Center (CDHDC)

# The Postnatal Growth Reference for Preterm Infants

## BMI(<28w girls))

| Corrected age(months) | Centiles | | | | | | |
|---|---|---|---|---|---|---|---|
| | 3rd | 10rd | 25rd | 50rd | 75rd | 90rd | 97rd |
| 0 | 9.88 | 11.24 | 12.57 | 13.01 | 15.59 | 17.05 | 18.62 |
| 1 | 10.58 | 11.94 | 13.28 | 14.76 | 16.29 | 17.75 | 19.32 |
| 2 | 11.23 | 12.58 | 13.92 | 15.40 | 16.92 | 18.37 | 19.93 |
| 3 | 11.80 | 13.15 | 14.47 | 15.94 | 17.44 | 18.87 | 20.40 |
| 4 | 12.31 | 13.63 | 14.93 | 16.36 | 17.83 | 19.23 | 20.73 |
| 5 | 12.73 | 14.02 | 15.28 | 16.68 | 18.11 | 19.46 | 20.92 |
| 6 | 13.03 | 14.29 | 15.53 | 16.89 | 18.28 | 19.60 | 21.02 |
| 7 | 13.19 | 14.44 | 15.66 | 16.99 | 18.37 | 19.67 | 21.06 |
| 8 | 13.23 | 14.47 | 15.68 | 17.01 | 18.37 | 19.67 | 21.05 |
| 9 | 13.17 | 14.41 | 15.62 | 16.95 | 18.32 | 19.61 | 20.99 |
| 10 | 13.03 | 14.28 | 15.49 | 16.84 | 18.21 | 19.51 | 20.90 |
| 11 | 12.86 | 14.10 | 15.33 | 16.67 | 18.05 | 19.36 | 20.76 |
| 12 | 12.66 | 13.91 | 15.13 | 16.48 | 17.85 | 19.16 | 20.57 |
| 13 | 12.45 | 13.70 | 14.92 | 16.26 | 17.63 | 18.94 | 20.34 |
| 14 | 12.26 | 13.49 | 14.70 | 16.04 | 17.40 | 18.70 | 20.09 |
| 15 | 12.08 | 13.30 | 14.49 | 15.81 | 17.16 | 18.45 | 19.82 |
| 16 | 11.93 | 13.13 | 14.31 | 15.60 | 16.93 | 18.20 | 19.55 |
| 17 | 11.82 | 13.00 | 14.15 | 15.41 | 16.71 | 17.94 | 19.26 |
| 18 | 11.77 | 12.91 | 14.02 | 15.25 | 16.51 | 17.70 | 18.98 |
| 19 | 11.75 | 12.85 | 13.93 | 15.12 | 16.34 | 17.50 | 18.73 |
| 20 | 11.74 | 12.82 | 13.87 | 15.03 | 16.21 | 17.33 | 18.53 |
| 21 | 11.74 | 12.80 | 13.82 | 14.95 | 16.11 | 17.20 | 18.37 |
| 22 | 11.74 | 12.77 | 13.79 | 14.90 | 16.04 | 17.12 | 18.27 |
| 23 | 11.72 | 12.75 | 13.75 | 14.86 | 15.99 | 17.06 | 18.20 |
| 24 | 11.69 | 12.72 | 13.73 | 14.83 | 15.96 | 17.03 | 18.18 |
| 25 | 11.65 | 12.69 | 13.70 | 14.81 | 15.95 | 17.02 | 18.17 |
| 26 | 11.60 | 12.64 | 13.66 | 14.78 | 15.93 | 17.02 | 18.18 |
| 27 | 11.54 | 12.59 | 13.62 | 14.75 | 15.91 | 17.01 | 18.18 |
| 28 | 11.47 | 12.53 | 13.57 | 14.72 | 15.89 | 17.00 | 18.18 |
| 29 | 11.40 | 12.48 | 13.52 | 14.68 | 15.86 | 16.98 | 18.18 |
| 30 | 11.34 | 12.42 | 13.48 | 14.64 | 15.84 | 16.97 | 18.18 |
| 31 | 11.28 | 12.37 | 13.43 | 14.61 | 15.81 | 16.95 | 18.17 |
| 32 | 11.23 | 12.32 | 13.39 | 14.58 | 15.79 | 16.94 | 18.17 |
| 33 | 11.18 | 12.28 | 13.36 | 14.55 | 15.77 | 16.92 | 18.16 |
| 34 | 11.13 | 12.24 | 13.32 | 14.52 | 15.74 | 16.91 | 18.15 |
| 35 | 11.08 | 12.19 | 13.28 | 14.49 | 15.72 | 16.89 | 18.14 |
| 36 | 11.02 | 12.14 | 13.24 | 14.45 | 15.69 | 16.87 | 18.13 |

By Wuhan University Children's Digital Health and Data Center (CDHDC)
